# Vitamin C for ≥ 5 days is associated with decreased hospital mortality in sepsis subgroups: a nationwide cohort study

**DOI:** 10.1186/s13054-021-03872-3

**Published:** 2022-01-05

**Authors:** Sun-Young Jung, Min-Taek Lee, Moon Seong Baek, Won-Young Kim

**Affiliations:** 1 College of Pharmacy, Chung-Ang University, Seoul, Republic of Korea; 2 Department of Global Innovative Drugs, The Graduate School of Chung-Ang University, Chung-Ang University, Seoul, Republic of Korea; 3 Division of Pulmonary and Critical Care Medicine, Department of Internal Medicine, Chung-Ang University Hospital, Chung-Ang University College of Medicine, Seoul, Republic of Korea

**Keywords:** Ascorbic acid, Mortality, Sepsis, Septic shock, Steroids, Thiamine

## Abstract

**Background:**

Previous randomized trials of vitamin C, hydrocortisone, and thiamine on sepsis were limited by short-term vitamin C administration, heterogeneous populations, and the failure to evaluate each component’s effect. The purpose of this study was to determine whether vitamin C alone for ≥ 5 days or in combination with corticosteroids and/or thiamine was associated with decreased mortality across the sepsis population and subpopulation.

**Methods:**

Nationwide population-based study conducted using the Korean National Health Insurance Service database. A total of 384,282 adult patients with sepsis who were admitted to the intensive care unit were enrolled from January 2017 to December 2019. The primary outcome was hospital mortality, while the key secondary outcome was 90-day mortality.

**Results:**

The mean [standard deviation] age was 69.0 [15.4] years; 57% were male; and 36,327 (9%) and 347,955 did and did not receive vitamin C, respectively. After propensity score matching, each group involved 36,327 patients. The hospital mortality was lower by − 0.9% in the treatment group (17.1% vs 18.0%; 95% confidence interval, − 1.3 to − 0.5%; *p* < 0.001), a significant but extremely small difference. However, mortality decreased greater in patients who received vitamin C for ≥ 5 days (vs 1–2 or 3–4 days) (15.8% vs 18.8% vs 18.3%; *p* < 0.001). Further, vitamin C was associated with a lower hospital mortality in patients with older age, multiple comorbidities, pneumonia, genitourinary infection, septic shock, and mechanical ventilation. Consistent findings were found for 90-day mortality. Moreover, vitamin C alone or in combination with thiamine was significantly associated with decreased hospital mortality.

**Conclusions:**

Intravenous vitamin C of ≥ 5 days was significantly associated with decreased hospital and 90-day mortality in sepsis patients. Vitamin C combined with corticosteroids and/or thiamine in specific sepsis subgroups warrants further study.

**Supplementary Information:**

The online version contains supplementary material available at 10.1186/s13054-021-03872-3.

## Background

Sepsis is a life-threating organ dysfunction caused by a dysregulated host response to infection [1]. Importantly, sepsis results in more than 5 million deaths globally each year, thus making it a major public health concern [2]. Accordingly, novel therapeutic interventions for sepsis have been explored over the last several decades. However, these have demonstrated inconsistent benefits [3].

Vitamin C downregulates proinflammatory cytokines; maintains the endothelial barrier; and facilitates the production of catecholamines, vasopressin, and cortisol [4, 5]. A recent retrospective study showed that the combination of vitamin C, hydrocortisone, and thiamine has a substantial survival benefit in patients with sepsis [6]. As such, numerous randomized trials of vitamin C alone or in combination with hydrocortisone and thiamine for sepsis have been conducted, although neither treatment was associated with significantly improved outcomes [7–10]. However, these trials had insufficient power for detecting differences in mortality and limited the use of vitamin C to a maximum of 4 days. Low-dose corticosteroids have been shown to reverse septic shock [11, 12], although the use of hydrocortisone was not controlled in most of these trials [7, 9, 10]. A recent network meta-analysis evaluated larger samples to estimate the effect of vitamin C, corticosteroids, and thiamine alone or in combination on mortality and other outcomes [13]. However, this analysis did not assess the treatment duration and may be limited by heterogeneity of the published studies.

To address current knowledge gaps, this study aimed to investigate whether vitamin C for ≥ 5 days was associated with improved hospital mortality across populations with sepsis. Toward this goal, data using the Korean National Health Insurance Service (NHIS) database were analyzed. Various subgroup analyses were also performed to analyze mortality differences according to sepsis subpopulation and combination with corticosteroids and/or thiamine.

## Methods

### Data source and study population

This retrospective cohort study collected data from the NHIS database, which consists of reimbursement claims from all types of healthcare facilities among over 97% of the population in Korea (51.1 million in 2018) [14]. The database contains inpatient information including demographics; primary and secondary diagnoses; admission history; hospital length of stay; discharge status; date of death (if applicable); diagnoses; and procedure and prescription charges. The reimbursement plan is based on fee-for-service and does not incentivize upcoding as reimbursement is not related to a specific diagnosis. Diagnoses were coded based on the International Classification of Diseases, 10th Revision (ICD-10), exclusively by attending physicians. There are no governmental mandates for sepsis care and reporting in Korea.

In this study, adult (age ≥ 18 years) patients with sepsis admitted to the intensive care unit (ICU) between January 2017 and December 2019 were evaluated. Sepsis was defined following the Sepsis-3 definition [1] as infection and organ dysfunction before hospital discharge, and the patients were identified using the ICD-10 codes for infection (Additional file 1: Appendix 1) and organ dysfunction (Additional file 2: Table S1). The presence of septic shock was determined according to the relevant ICD-10 code (R57.2) or the administration of vasopressors such as norepinephrine, epinephrine, vasopressin, and dopamine. According to the Sepsis-3 criteria [1], vasopressor use equates to a score greater than or equal to 2 in the Sequential Organ Failure Assessment (SOFA) cardiovascular subscore and was used as a reasonable surrogate of sepsis severity. The exclusion criteria were age < 18 years; pregnancy or related condition; palliative care; cardiac arrest; and multiple ICU admissions. In the NHIS database, it is not feasible to temporally associate diagnoses (sepsis) with prescription charges (vitamin C) during hospitalization due to lack of time stamps, and immortal time bias may be a significant concern. To minimize bias, two analytical approaches were employed, namely, (1) an analysis excluding patients who died or were discharged within 2 days of ICU admission to prevent patients who died early in their hospital course from introducing bias and (2) an analysis assessing outcomes with stratification according to the follow-up period given the study intervention is not temporally defined [15].

The need for ethical approval was waived by the Institutional Review Board of Chung-Ang University owing to the de-identified patient data (1041078-202001-HR-012-01). This study complied with the Strengthening the Reporting of Observational Studies in Epidemiology guidelines.

### Vitamin C, corticosteroids, and thiamine

Patients who did and did not receive at least one charge for high-dose intravenous (IV) vitamin C during the index hospitalization were categorized into the treatment and control groups, respectively. Corticosteroid or thiamine use was defined as at least one charge for IV corticosteroids or IV thiamine during the index hospitalization, respectively. The daily dose of each drug was not calculated because drug prescriptions do not necessarily reflect actual treatment. However, the dosing of vitamin C may have been quite consistent around 6 g/day based on previous studies conducted in Korea [16, 17]. Total duration of use was determined for all three drugs. The detailed codes for IV vitamin C, corticosteroids, and thiamine are shown in Additional file 2: Table S2.

### Data collection, definitions, and outcomes

Baseline patient characteristics included age; sex; Charlson comorbidities [18] defined using claim records within 1 year prior to admission; immunosuppression (malignancy, human immunodeficiency virus infection, organ transplant, or immunosuppressive therapy); previous steroid use (oral or IV for ≥ 30 days during the preceding year); emergency department presentation; and admitting department. The sites of infection were identified using ICD-10 codes (Additional file 2: Table S3), and hospital capacity was measured by the number of beds. Procedure codes were used to extract clinical care variables such as conventional oxygen therapy, high-flow nasal cannula, mechanical ventilation, and renal replacement therapy.

The primary outcome was hospital mortality. The secondary outcomes were 90-day mortality, vasopressor days, ventilator days, ICU and hospital lengths of stay, and hospital costs.

### Statistical analysis

Continuous data were reported as the mean (standard deviation, SD) and were compared using Student’s *t*-test or Kruskal–Wallis test, as appropriate. Categorical data were reported as the number (percentage) and were compared using chi-square test or Fisher’s exact test, as appropriate. Outcome data were presented as the mean of all paired differences with 95% confidence intervals (CIs). Propensity score (PS) analysis was performed to create a matched cohort of patients who had differences in the administration of vitamin C but were similar in other measured variables [19]. The conditional probability of patients to receive vitamin C given the individual covariates is unclear. Thus, PS in each patient was estimated using logistic regression model that included all available baseline covariates (Table 1), except for thiamine use. Patients in the treatment and control groups were matched 1:1 without replacement, using nearest neighbor matching based on a greedy matching algorithm [20]. To ensure good matches, a caliper of 0.2 of the SD of the logit of the PS was defined. Standardized mean differences were calculated among covariates before and after matching, and a difference of ≤ 0.10 was considered well-balanced. Survival to 90 days was plotted using Kaplan–Meier curves and compared between the PS-matched treatment and control groups using log-rank tests. Logistic regression analysis was performed to identify the risk factors for hospital mortality.

**Table 1:** Baseline patient characteristics in the matched cohort

Characteristics	Vitamin C (*n* = 36,327)	Control (*n* = 36,327)	SMD
Age, mean (SD), years	70.7 (15.2)	70.7 (15.5)	0.03
Sex, No. (%)			− 0.02
Male	20,093 (55)	19,919 (55)	
Female	16,234 (45)	16,408 (45)	
Comorbidities, No. (%)			
Diabetes	15,369 (42)	15,024 (41)	0.02
Hypertension	24,236 (67)	23,996 (66)	0.01
Myocardial infarction	1224 (3)	1109 (3)	0.02
Congestive heart failure	6154 (17)	5795 (16)	0.03
Cerebrovascular disease	9683 (27)	9483 (26)	0.01
Chronic pulmonary disease	16,133 (44)	15,852 (44)	0.02
Chronic liver disease	11,268 (31)	11,132 (31)	0.008
Chronic kidney disease	3929 (11)	3651 (10)	0.03
Malignancy	6965 (19)	6679 (18)	0.02
Charlson Comorbidity Index, mean (SD)	3.0 (1.9)	2.9 (1.9)	0.05
Immunosuppression, No. (%)^a^	10,313 (28)	9860 (27)	0.03
Previous steroid use, No. (%)^b^	4461 (12)	4216 (12)	0.02
Site of infection, No. (%)			
Lung	11,088 (31)	10,919 (30)	0.01
Gastrointestinal tract	4801 (13)	4570 (13)	0.02
Genitourinary tract	4644 (13)	4518 (12)	0.01
Hospital capacity, No. (%)			0.04
< 500 beds	18,373 (51)	18,477 (51)	
500–1000 beds	14,259 (39)	14,546 (40)	
≥ 1000 beds	3695 (10)	3304 (9)	
Presented to emergency department, No. (%)	28,337 (78)	28,602 (79)	− 0.02
Medical department, No. (%)	25,723 (71)	25,455 (70)	0.02
Septic shock, No. (%)	18,077 (50)	17,569 (48)	0.03
Organ dysfunction, No. (%)			
Cardiovascular	18,325 (50)	17,793 (49)	0.03
Respiratory	33,080 (91)	33,133 (91)	− 0.005
Neurologic	2008 (6)	1848 (5)	0.02
Hematologic	1032 (3)	958 (3)	0.01
Hepatic	621 (2)	634 (2)	− 0.003
Renal	4801 (13)	4453 (12)	0.03
Metabolic	470 (1)	481 (1)	− 0.003
No. of organ dysfunctions, No. (%)			0.05
1	14,955 (41)	15,697 (43)	
2	14,870 (41)	14,602 (40)	
3	4290 (12)	3940 (11)	
≥ 4	706 (2)	702 (2)	
Corticosteroids, No. (%)	13,968 (38)	13,759 (38)	0.01
Thiamine, No. (%)	7745 (21)	3517 (10)	0.33
Conventional oxygen therapy, No. (%)	31,670 (87)	31,858 (88)	− 0.02
High-flow nasal cannula, No. (%)	4059 (11)	3862 (11)	0.02
Mechanical ventilation, No. (%)	10,793 (30)	10,404 (29)	0.02
Renal replacement therapy, No. (%)	2628 (7)	2494 (7)	0.01

*SMD* standardized mean difference

^a^Immunosuppression included malignancy, human immunodeficiency virus infection, organ transplant, or immunosuppressive therapy

^b^Defined by oral or intravenous for ≥ 30 days during the preceding year

The duration–response relationship between vitamin C and clinical outcomes was assessed by comparing vitamin C for 1–2 days, 3–4 days, and ≥ 5 days. The cut-off values were based on a recent meta-analysis [17]. The duration of ≥ 5 days was selected based on a hypothesis that limiting the use of vitamin C to 4 days, which was assessed in most previous studies, may not translate to a survival benefit. Subgroup analyses for the primary and secondary outcomes were performed on subpopulations determined a priori from baseline covariates associated with hospital mortality. These included age ≥ 70 or < 70 years; male or female; Charlson Comorbidity Index (CCI) ≥ 3 or < 3; pneumonia; gastrointestinal infection; genitourinary infection; septic shock; mechanical ventilation; and renal replacement therapy. Pneumonia was selected on the basis that vitamin C depletion and elevated oxidative stress were observed in patients with pneumonia sepsis [21], and vitamin C administration may decrease oxidative stress and improve survival. Moreover, vitamin C may reduce genitourinary infection by production of reactive nitrogen species and inhibition of biofilm formation on the urinary catheters [22, 23]. Two sensitivity analyses were performed, namely, (1) an analysis including only patients who survived to hospital discharge and (2) an analysis between patients who received vitamin C for ≥ 5 days and matched controls. Subgroup analyses including vitamin C only versus various combinations with corticosteroids and/or thiamine with stratification according to the duration of use (< 5 days vs ≥ 5 days) were also performed. The PS-matched odds ratios (ORs) and 95% CIs for hospital mortality were calculated and shown using forest plots.

There were no missing values in the dataset. No adjustment was made for multiple comparisons, and the findings of secondary analyses should be considered hypothesis generating. All analyses were performed using SAS software (version 9.4; SAS Institute, Cary, NC, USA). A two-tailed *p* value of < 0.05 was considered statistically significant.

## Results

### Patient characteristics

**Fig. 1:**
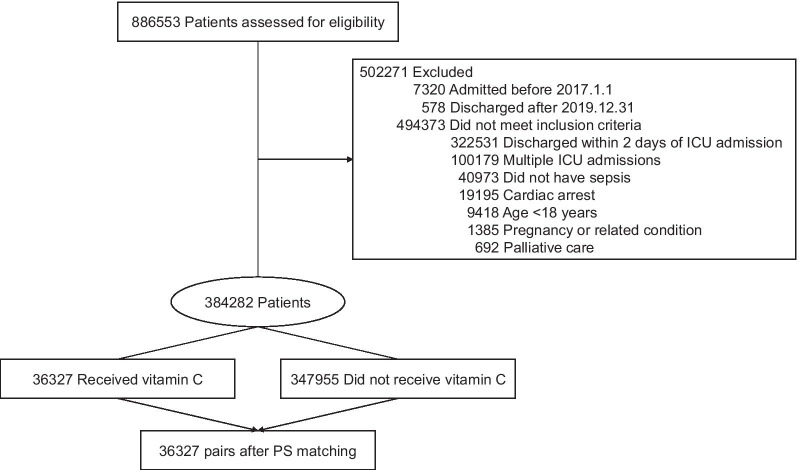
Flow of patients in the nationwide cohort study for vitamin C in sepsis. *ICU* intensive care unit, *PS* propensity score

Among the 886,553 eligible patients, 502,271 were excluded (Fig. 1). Thus, 384,282 patients were included in the analysis. Of them, 36,327 (9%) and 347,955 patients were categorized to the treatment and control groups, respectively. The baseline characteristics of the unmatched patients are shown in Additional file 2: Table S4. The mean (SD) age was 69.0 (15.4) years, and 57% were male. The treatment group were more likely to be older, have a higher CCI, have pneumonia and genitourinary infection, and receive thiamine. Meanwhile, the control group were more likely to be admitted in a higher volume hospital, have septic shock, and require mechanical ventilation.

### Propensity score analysis

After matching, each group involved 36,327 patients. The baseline characteristics were generally well-matched between the two groups (Table 1). The major sites of infection were the lungs, gastrointestinal tract, and genitourinary tract, and the most common type of organ dysfunction was respiratory failure. Almost half of the patients had septic shock. There was no significant between-group difference in the use of corticosteroids. Meanwhile, there were significant differences in hospital mortality (17.1% vs 18.0%; difference, − 0.9%; 95% CI − 1.3 to − 0.5%; *p* < 0.001) and 90-day mortality (25.4% vs 27.0%; difference, − 3.2%; 95% CI − 3.8 to − 2.6%; *p* < 0.001) (Table 2). These observations were not influenced when the patients were stratified according to the length of ICU stay (≤ 5 days vs > 5 days) (Additional file 2: Table S5).

**Table 2:** Primary and secondary outcomes

Outcomes	Vitamin C (*n* = 36,327)	Control (*n* = 36,327)	Difference (95% CI)	*p* value
Primary outcome				
Hospital mortality, No. (%)	6209 (17.1)	6538 (18.0)	− 0.9 (− 1.3 to − 0.5)^a^	< 0.001
Secondary outcomes				
90-day mortality, No. (%)	9226 (25.4)	9820 (27.0)	− 3.2 (− 3.8 to − 2.6)	< 0.001
Vasopressor days, mean (SD)	2.9 (2.3) [*n* = 17923]	2.7 (2.0) [*n* = 17441]	0.12 (0.1 to 0.2)	0.002
Ventilator days, mean (SD)	9.5 (18.8) [*n* = 10793]	8.2 (15.7) [*n* = 10404]	1.4 (1.3 to 1.5)	< 0.001
Length of stay, mean (SD), days				
ICU	10.6 (17.5)	8.6 (14.2)	2.02 (2.0 to 2.1)	< 0.001
Hospital	27.1 (41.6)	21.0 (22.1)	6.0 (5.9 to 6.1)	< 0.001
Hospital costs, mean (SD), U.S. $1000	13.3 (19.0)	12.1 (25.2)	1.19 (1.18 to 1.2)	< 0.001

*ICU* intensive care unit

^a^The mean of all paired differences in the treatment group minus the control group

**Fig. 2:**
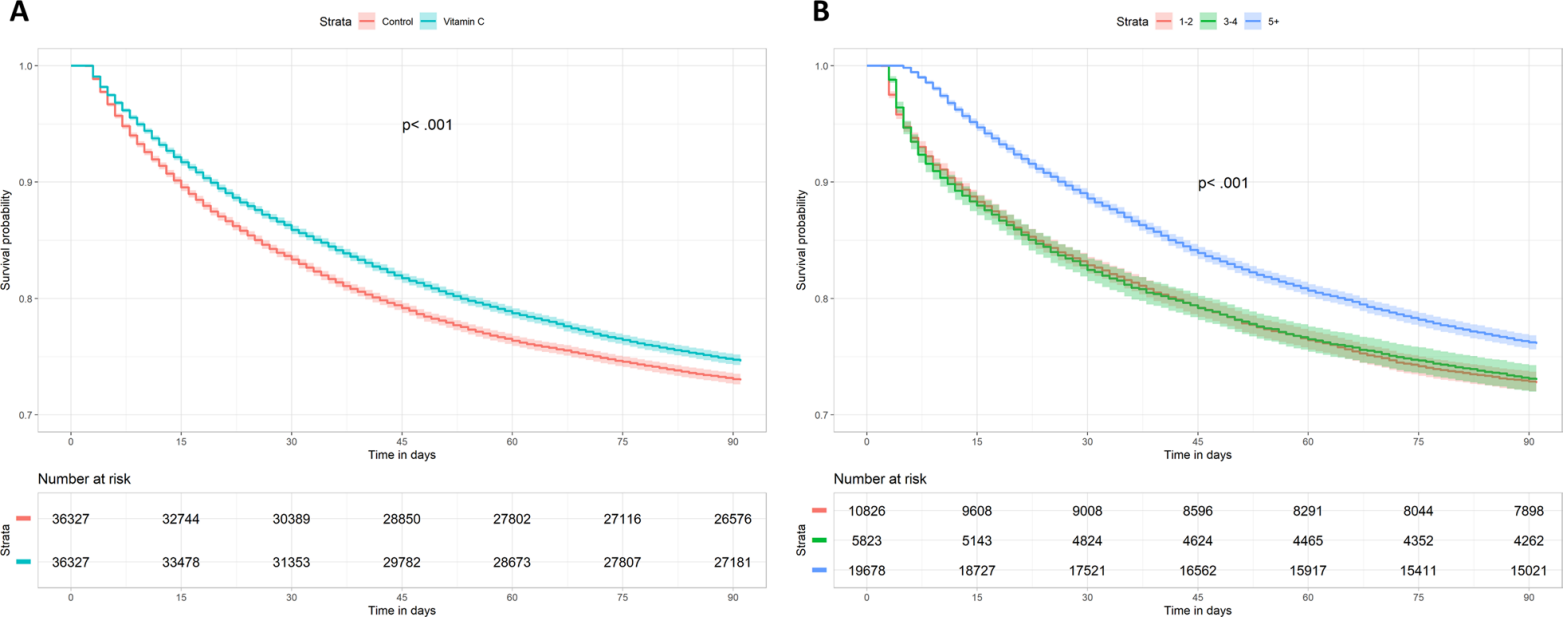
Survival from hospital admission to day 90. **A** By study group. The median (interquartile range) time to death was 25 (11–48) days in the treatment group and 21 (9–42) days in the control group. **B** By duration of vitamin C use

The survival curves were also significantly different between the two groups on log-rank test (*p* < 0.001) (Fig. 2a). Vasopressor days, ventilator days, ICU and hospital lengths of stay, and hospital costs were significantly higher in the treatment group (Table 2). Sensitivity analysis of patients who survived to hospital discharge demonstrated similar findings (Additional file 2: Table S6). Vitamin C use was associated with a significantly lower risk of hospital mortality (PS-matched OR, 0.94; 95% CI 0.90–0.97) (Additional file 2: Table S7). Conversely, the use of corticosteroids was associated with a significantly higher risk of mortality (PS-matched OR, 1.94; 95% CI 1.82–2.07).

### Vitamin C duration and mortality

Of the 36,327 patients in the treatment group, 10,826 (30%) patients received vitamin C for 1–2 days; 5823 (16%) patients, 3–4 days; and 19,678 (54%) patients, ≥ 5 days. Among patients who received vitamin C for ≥ 5 days, the median (interquartile range) duration of use was 11 (7 to 17) days. Compared with patients who received vitamin C for 1–2 or 3–4 days, those treated for ≥ 5 days showed significantly lower hospital (18.8% vs 18.3% vs 15.8%; *p* < 0.001) and 90-day mortality (27.3% vs 27.0% vs 23.9%; *p* < 0.001) rates (Additional file 2: Table S8 and Fig. 2b). Patients who received vitamin C for ≥ 5 days had significantly lower hospital and 90-day mortality rates compared to PS-matched controls (Additional file 2: Table S9). These findings were not influenced when the patients were stratified according to the length of ICU stay (≤ 5 days vs > 5 days) (Additional file 2: Table S10). Vasopressor days, ventilator days, and ICU and hospital lengths of stay were also significantly higher in these patients (Additional file 2: Table S8).

### Subgroup analyses

The primary and secondary outcomes among different subpopulations with sepsis are shown in Additional file 2: Table S11. Vitamin C use was associated with significantly lower risks of hospital and 90-day mortalities in patients with older age (≥ 70 years), a higher number of comorbidities (CCI ≥ 3), pneumonia, genitourinary infection, septic shock, and mechanical ventilation. Conversely, mortality did not differ according to gastrointestinal infection and renal replacement therapy between the treatment and control groups. There were also no sex differences in mortality, but it was significantly lower for patients who received vitamin C. In general, patients who received vitamin C had significantly higher vasopressor days, ventilator days, ICU and hospital lengths of stay, and hospital costs across all subgroups. The results of sensitivity analyses of patients who received vitamin C for ≥ 5 days were similar to the primary analyses, although mortality among patients with renal replacement therapy was significantly lower when vitamin C was administered (Additional file 2: Table S12). The differential survival probabilities by subgroups are demonstrated in Additional file 3: Figs. S1 and S2.

### Vitamin C only vs combination therapy

**Fig. 3:**
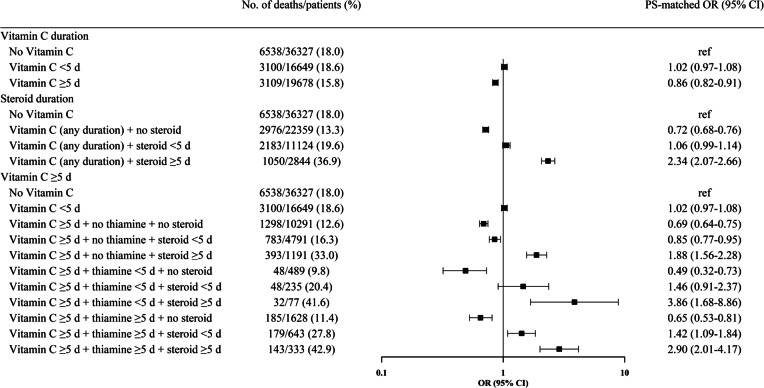
Association between vitamin C monotherapy and hospital mortality compared with in combination with corticosteroids and/or thiamine. The numbers and percentages of patients who died according to each drug combination are shown. The odds ratios (ORs) and 95% confidence intervals (CIs) are calculated in the propensity score (PS)-matched treatment and control groups

Figure 3 shows the results of subgroup analyses regarding the association between vitamin C only and hospital mortality compared with a combined therapy with corticosteroids and/or thiamine. Forest plots for combinations demonstrated that vitamin C alone (any duration) or the combination of vitamin C (any duration) plus thiamine (any duration) was significantly associated with decreased mortality. However, any combination of vitamin C and corticosteroids, regardless of thiamine use, was significantly associated with increased mortality. The results of the primary analyses were generally consistent across all subpopulations (Additional file 3: Fig. S3), although the combination of vitamin C (any duration) plus corticosteroids < 5 days was significantly associated with decreased mortality among patients with septic shock and mechanical ventilation.

## Discussion

This nationwide cohort study with PS analysis found a significantly lower hospital mortality among patients with sepsis treated with IV vitamin C of ≥ 5 days. These findings were consistent for patients with older age, multiple comorbidities, pneumonia, septic shock, and mechanical ventilation. In addition, vitamin C alone or in combination with thiamine, but not with corticosteroids, was associated with improved mortality.

The hospital mortality rates of patients with sepsis and septic shock were 17.1% and 28.7%, respectively. These are in line with the 10% mortality rate in the Sepsis-3 criteria [1] and the 24.8–28.2% mortality rates of septic shock in a recent study using a US healthcare database [24]. To date, several randomized trials have evaluated the efficacy of vitamin C as an adjunctive therapy for sepsis, but the results have been conflicting [7–10]. For the primary outcomes, these trials used SOFA scores and ventilator- and vasopressor-free days instead of mortality owing to lack of power. In addition, a considerable proportion of patients received corticosteroids, further diluting the effect size. The main strength of this study is the use of the largest national data in existence to assess the association between vitamin C and mortality in sepsis patients while controlling for the use of corticosteroids. Another strength of the study was that there were no missing values in the database. The results revealed a decrease of both hospital and long-term mortality in patients who received vitamin C even after PS matching. However, 0.9–1.6% differences in mortality may not be considered to be clinically important compared with a large effect size demonstrated in previous observational studies [6].

Sepsis could downregulate the cellular sodium-dependent vitamin C transporters [25]. Thus, whether vitamin C saturation in the plasma reflects comparable saturation of septic tissues is unclear. The present results demonstrated that patients who received vitamin C for ≥ 5 days had a significantly lower mortality. This finding may be important and clinically relevant, given that most previous studies, which failed to show survival benefit, limited the use of vitamin C to 4 days. Despite high-dose administration of vitamin C, patients may experience hypovitaminosis C within several hours of discontinuation [26]. In the CITRIS-ALI trial, the survival curve of the vitamin C group became parallel to that of the placebo group after cessation of treatment [7], suggesting that the favorable effects of vitamin C may decrease over time. The lack of consistent benefits in previous trials of vitamin C in sepsis may also be due to insufficient dosage. For instance, the ACTS trial, which showed negative results, evaluated patients who received only 1 dose of study drug [9]. Conversely, the CITRIS-ALI trial tested a higher dose of vitamin C (200 mg/kg/day) and found a significant decrease in mortality among patients with sepsis and acute respiratory distress syndrome [7]. Interestingly, a recent meta-analysis revealed the associations between high-dose (≥ 6 to < 12 g/day) and very high-dose (≥ 12 g/day) vitamin C and decreased mortality [13].

The outcomes in previous trials may have been influenced by patient heterogeneity. Moreover, there is a possibility that vitamin C may also be beneficial in certain subgroups. In many cases, clinical trials of interventions for sepsis excluded very elderly (≥ 70 years) patients with multiple comorbidities due to a high risk of death and low treatment response. However, these patients accounted for a large proportion (59%) of the present population who showed lower mortalities when vitamin C was administered. Notably, there was no mortality decrease with vitamin C in patients with younger age (< 70 years) and less comorbidities, suggesting that treating specific subgroups may have resulted in improved overall mortality. The association between vitamin C and improved mortality was also greater for patients with pneumonia and mechanical ventilation. Vitamin C administration to peripheral blood monocytes from pneumonia patients decreased proinflammatory cytokines [27]. Furthermore, several studies have shown that vitamin C decreased the duration of mechanical ventilation and mortality in patients with severe pneumonia [16, 28]. In the current study, vitamin C was significantly associated with a lower hospital mortality in patients with septic shock, but not in patients with renal replacement therapy. A possible explanation for this may be vitamin C loss by filtration, dialysis, or non-reabsorption [29]. Long-term vitamin C might be useful, as the present data revealed that patients in this subgroup who received vitamin C for ≥ 5 days had a significantly lower mortality.

Previous trials did not evaluate the individual effects of vitamin C and thiamine. In this study, vitamin C was associated with increased mortality in sepsis patients in the presence of corticosteroids. In addition, corticosteroid use was associated with a higher risk of mortality after adjusting for confounding due to baseline severity. The increased risk of neuromuscular weakness from corticosteroids among relatively older patients in the present study might explain these findings [30]. Meanwhile, mortality was significantly lower for patients with septic shock and mechanical ventilation who received short-term corticosteroids. These are in line with recent guidelines that advocate for the use of corticosteroids in septic shock [31, 32]. However, it may be noted that several meta-analyses showed that vitamin C monotherapy was more beneficial than when administered in combination with corticosteroids [13, 33]. Interestingly, the combination of vitamin C and thiamine was associated with a further lower mortality in certain subgroups, while other studies have not shown this finding [13, 33]. Additional studies are required to determine potential optimized drug combinations.

This study has several limitations. First, its non-randomized design precludes any inference of causality on association between vitamin C use and mortality. Moreover, a retrospective design is vulnerable to unmeasured confounders, although the groups were balanced with respect to measured confounders using PS analysis. Second, vital signs and laboratory data were lacking in the database, but diagnoses, prescriptions, and procedures were used as surrogates of disease severity. Third, sepsis definitions which are based on administrative codes can be affected by coding practices unrelated to the physiologic data [34, 35]. Moreover, sepsis was defined as infection and organ dysfunction within the same admission, but a time difference may exist. Fourth, analytical methods to address immortal time bias may be insufficient due to inability to temporally associate diagnoses with treatments. Data on timing of ICU admission, vasopressor initiation, or vitamin C administration were unavailable due to lack of time stamps. Thus, whether early or delayed vitamin C treatment is associated with outcomes among sepsis patients could not be assessed in the present study. Fifth, total vasopressor days, ventilator days, and ICU and hospital lengths of stay were calculated because the sequence of events within each admission was not available. In addition, the hospital costs were all-cause estimates and not the attributable costs of sepsis. These might explain the findings that vitamin C was associated with a lower hospital mortality but not with improvements in secondary outcomes. Sixth, this study did not assess the outcome of different doses of vitamin C. Seventh, vitamin C levels were not available, and patients in this study were not screened based on their vitamin C status.

## Conclusions

In this nationwide cohort study of patients with sepsis, intravenous vitamin C is associated with a lower hospital mortality. However, the present results should be interpreted with caution because the difference of mortality was extremely small. The finding that vitamin C of ≥ 5 days was associated with an even greater decrease in mortality is consistent with previous meta-analysis suggesting that there may be a dose–response relationship between vitamin C and mortality [13]. Additional randomized trials evaluating very high-dose, long-term vitamin C for sepsis are warranted. Further studies are also needed to evaluate the efficacy of vitamin C in specific subpopulations with sepsis that may benefit from treatment, using optimized combination with corticosteroids and/or thiamine.

## Supplementary Information

**Additional file 1: Appendix 1.** ICD-10 codes used for identification of infectious condition.

**Additional file 2: Table S1**. ICD-10-based classification of organ dysfunction. **Table S2**. Anatomical Therapeutic Chemical codes and charge codes for intravenous vitamin C, corticosteroids, and thiamine. **Table S3**. ICD-10 codes for different sites of infection. **Table S4**. Baseline patient characteristics in the unmatched cohort. **Table S5**. Primary and secondary outcomes according to ICU length of stay. **Table S6**. Secondary outcomes of patients who survived to hospital discharge. **Table S7**. Risk factors for hospital mortality. **Table S8**. Primary and secondary outcomes according to vitamin C duration. **Table S9**. Primary and secondary outcomes in patients who received vitamin C for ≥ 5 days and in matched controls. **Table S10**. Primary and secondary outcomes according to ICU length of stay in patients who received vitamin C for ≥ 5 days and in matched controls. **Table S11**. Primary and secondary outcomes by subpopulations. **Table S12**. Primary and secondary outcomes by subpopulation in patients who received vitamin C for ≥ 5 days and in matched controls.

**Additional file 3: Fig. S1.** Survival from hospital admission to day 90 by subpopulations. **Fig. S2**. Survival from hospital admission to day 90 by sepsis subpopulations among patients who received vitamin C for ≥5 days and matched controls. **Fig. S3**. Association between vitamin C monotherapy and hospital mortality compared with in combination with corticosteroids and/or thiamine in the sepsis subpopulations.

## Data Availability

All data generated or analyzed during this study are included in this published article and its supplementary information files.

## Abbreviations

CCI: Charlson Comorbidity Index
CI: Confidence interval
ICD: International Classification of Diseases
ICU: Intensive care unit
IV: Intravenous
NHIS: National Health Insurance Service
OR: Odds ratio
PS: Propensity score
SD: Standard deviation
SOFA: Sequential Organ Failure Assessment
