# Supplementary material for: Vitamin C for ≥ 5 days is associated with decreased hospital mortality in sepsis subgroups: a nationwide cohort study
Source: Crit Care. 2022 Jan 5;26:3. doi: 10.1186/s13054-021-03872-3 (PMC8728994; doi:10.1186/s13054-021-03872-3)
Supplement: Supplementary file 1 — Additional file 1: Appendix 1. ICD-10 codes used for identification of infectious condition. [file 13054_2021_3872_MOESM1_ESM.docx]

**Appendix 1** ICD-10 codes used for identification of infectious condition

A00, Cholera; A01, Typhoid and paratyphoid fevers; A02, Other salmonella infections; A03, Shigellosis; A04, Other bacterial intestinal infections; A05, Other bacterial foodborne intoxications, not elsewhere classified (NEC); A09, Other gastroenteritis and colitis of infectious and unspecified origin; A20, Plague; A21, Tularaemia; A23, Brucellosis; A24, Glanders and Melioidosis; A25, Rat-bite fevers; A26, Erysipeloid; A27, Leptospirosis; A28, Other zoonotic bacterial diseases, NEC; A31, Infection due to other mycobacteria; A32, Listeriosis; A35, Other tetanus; A36, Diphtheria; A37, Whooping cough; A38, Scarlet fever; A39, Meningococcal infection; A40, Streptococcal sepsis; A41, Other sepsis; A42, Actinomycosis; A43, Nocardiosis; A44, Bartonellosis; A46, Erysipelas; A48, Other bacterial diseases, NEC; A49, Bacterial infection of unspecified site; A54, Gonococcal infection; A55, Chlamydial lymphogranuloma (venereum); A56, Other sexually transmitted chlamydial diseases; A57, Chancroid; A58, Granuloma inguinale; A63, Other predominantly sexually transmitted diseases, NEC; A64, Unspecified sexually transmitted disease; A68, Relapsing fevers; A69, Other spirochaetal infections; A70, Chlamydia psittaci infection; A74, Other diseases caused by chlamydiae; A75, Typhus fever; A77, Spotted fever (tick-borne rickettsioses); A78, Q fever; A79, Other rickettsioses; B37, Candidiasis; B38, Coccidioidomycosis; B39, Histoplasmosis; B40, Blastomycosis; B41, Paracoccidioidomycosis; B42, Sporotrichosis; B43, Chromomycosis and pheomycotic abscess; B44, Aspergillosis; B45, Cryptococcosis; B46, Zygomycosis; B48, Other mycoses, NEC; B49, Unspecified mycosis; B99, Other and unspecified infectious diseases; D71, Progressive septic granulomatosis; D733, Abscess of spleen; D738, Perisplenitis; E060, Abscess of thyroid; E321, Abscess of thymus; G00, Bacterial meningitis, NEC; G01, Meningitis in bacterial diseases classified elsewhere; G02, Meningitis in other infectious and parasitic diseases classified elsewhere; G03, Meningitis due to other and unspecified causes; G04, Encephalitis, myelitis and encephalomyelitis; G05, Encephalitis, myelitis and encephalomyelitis in diseases classified elsewhere; G06, Intracranial and intraspinal abscess and granuloma; G07, Intracranial and intraspinal abscess and granuloma in diseases classified elsewhere; G08, Intracranial and intraspinal phlebitis and thrombophlebitis; H050, Acute inflammation of orbit; H440, Purulent endophthalmitis; H441, Other endophthalmitis; H600, Abscess of external ear; H601, Cellulitis of external ear; H602, Malignant otitis externa; H603, Other infective otitis externa; H608, Other otitis externa; H609, Otitis externa, unspecified; H610, Perichondritis of external ear; H620, Otitis externa in bacterial diseases classified elsewhere; H622, Otitis externa in mycoses; H623, Otitis externa in other infectious and parasitic diseases classified elsewhere; H624, Otitis externa in other diseases classified elsewhere; H66, Suppurative and unspecified otitis media; H67, Otitis media in diseases classified elsewhere; H70, Mastoiditis and related conditions; H750, Mastoiditis in infectious and parasitic diseases classified elsewhere; I30, Acute pericarditis; I32, Pericarditis in diseases classified elsewhere; I33, Acute and subacute endocarditis; I38, Endocarditis, valve unspecified; I39, Endocarditis and heart valve disorders in disease classified elsewhere; I518, Carditis (acute, chronic); I80, Phlebitis and thrombophlebitis; I831, Varicose veins of lower extremities with inflammation; I832, Varicose veins of lower extremities with both ulcer and inflammation; I88, Nonspecific lymphadenitis; I891, Lymphangitis; J01, Acute sinusitis; J02, Acute pharyngitis; J03, Acute tonsillitis; J04, Acute laryngitis and tracheitis; J05, Acute obstructive laryngitis (croup) and epiglottitis; J13, Pneumonia due to *Streptococcus pneumoniae*; J14, Pneumonia due to *Hemophilus influenzae*; J15, Bacterial pneumonia, NEC; J16, Pneumonia due to other infectious organisms, NEC; J17, Pneumonia in diseases classified elsewhere; J18, Pneumonia, organism unspecified; J20, Acute bronchitis; J21, Acute bronchiolitis; J22, Unspecified acute lower respiratory infection; J340, Abscess, furuncle and carbuncle of nose; J36, Peritonsillar abscess; J383, Abscess of vocal cord(s); J387, Abscess of larynx; J390, Retropharyngeal and parapharyngeal abscess; J391, Other abscess of pharynx; J440, Chronic obstructive pulmonary disease with acute lower respiratory infection; J441, Chronic obstructive pulmonary disease with acute exacerbation, unspecified; J690, Pneumonitis due to food and vomit; J85, Abscess of lung and mediastinum; J86, Pyothorax; J90, Pleurisy with effusion; J91, Pleural effusion in conditions classified elsewhere; J950, Sepsis of tracheostomy stoma; J985, Mediastinitis; J986, Diaphragmatitis; K044, Acute apical periodontitis of pulpal origin; K046, Periapical abscess with sinus; K047, Periapical abscess without sinus; K050, Acute gingivitis; K052, Acute periodontitis; K102, Inflammatory conditions of jaws; K103, Alveolitis of jaws; K112, Sialoadenitis; K113, Abscess of salivary gland; K122, Cellulitis and abscess of mouth; K140, Abscess of tongue; K221, Fungal ulcer of oesophagus; K223, Perforation of oesophagus; K251, Acute gastric ulcer with perforation; K252, Acute gastric ulcer with both hemorrhage and perforation; K255, Chronic or unspecified gastric ulcer with perforation; K256, Chronic or unspecified gastric ulcer with both hemorrhage and perforation; K261, Acute duodenal ulcer with perforation; K262, Acute duodenal ulcer with both hemorrhage and perforation; K265, Chronic or unspecified duodenal ulcer with perforation; K266, Chronic or unspecified duodenal ulcer with both hemorrhage and perforation; K271, Acute peptic ulcer, site unspecified with perforation; K272, Acute peptic ulcer, site unspecified with both hemorrhage and perforation; K275, Chronic or unspecified peptic ulcer, site unspecified with perforation; K276, Chronic or unspecified peptic ulcer, site unspecified with both hemorrhage and perforation; K281, Acute gastrojejunal ulcer with perforation; K282, Acute gastrojejunal ulcer with both hemorrhage and perforation; K285, Chronic or unspecified gastrojejunal ulcer with perforation; K286, Chronic or unspecified gastrojejunal ulcer with both hemorrhage and perforation; K35, Acute appendicitis; K36, Other appendicitis; K37, Unspecified appendicitis; K383, Fistula of appendix; K388, Intussusception of appendix; K401, Bilateral inguinal hernia, with gangrene; K404, Unilateral or unspecified inguinal hernia, with gangrene; K411, Bilateral femoral hernia, with gangrene; K414, Unilateral or unspecified femoral hernia, with gangrene; K421, Umbilical hernia with gangrene; K431, Ventral hernia with gangrene; K441, Diaphragmatic hernia with gangrene; K451, Other specified abdominal hernia with gangrene; K461, Unspecified abdominal hernia with gangrene; K550, Acute vascular disorders of intestine; K551, Chronic vascular disorders of intestine; K559, Vascular disorders of intestine, unspecified; K561, intussusception; K562, Volvulus; K563, Gallstone ileus; K564, Other impaction of intestine; K565, Intestine adhesions (bands) with obstruction; K566, Other and unspecified intestinal obstruction; K567, Ileus, unspecified; K57, Diverticulitis of intestine (small, large); K60, Fissure and fistula of anal and rectal regions; K61, Abscess of anal and rectal regions; K630, Abscess of intestine; K631, Perforation of intestine (nontraumatic); K632, Fistula of intestine; K65, Peritonitis; K67, Disorders of peritoneum in infectious diseases classified elsewhere; K75, Other inflammatory liver diseases; K770, Liver disorders in infectious and parasitic diseases classified elsewhere; K800, Calculus of gallbladder with acute cholecystitis; K801, Calculus of gallbladder with other cholecystitis; K803, Calculus of bile duct with cholangitis; K804, Calculus of bile duct with cholecystitis; K81, Cholecystitis; K820, Obstruction of gallbladder; K822, Perforation of gallbladder; K823, Fistula of gallbladder; K830, Cholangitis; K831, Obstruction of bile duct; K832, Perforation of bile duct; K833, Fistula of bile duct; K85, Acute pancreatitis; K868, Pancreatic necrosis not otherwise specified (NOS); K931, Megacolon in Chagas’ disease (B57.3+); L00, Staphylococcal scalded skin syndrome; L01, Impetigo; L02, Cutaneous abscess, furuncle and carbuncle; L03, Cellulitis; L04, Acute lymphadenitis; L050, Pilonidal cyst with abscess; L08, Other local infections of skin and subcutaneous tissue; M00, Pyogenic arthritis; M01, Direct infections of joint in infectious and parasitic diseases classified elsewhere; M462, Osteomyelitis of vertebra; M463, Infection of intervertebral disc (pyogenic); M464, Discitis, unspecified; M465, Other infective spondylopathies; M491, Brucella spondylitis (A23.-+); M492, Enterobacterial spondylitis (A01-A04+); M493, Spondylopathy in other infectious and parasitic diseases classified elsewhere; M600, Infective myositis; M630, Myositis in bacterial diseases classified elsewhere; M632, Myositis in other infectious diseases classified elsewhere; M650, Abscess of tendon sheath; M651, Other infective (teno) synovitis; M680, Synovitis and tenosynovitis in bacterial diseases classified elsewhere; M710, Abscess of bursa; M711, Other infective bursitis; M715, Other bursitis NEC; M719, Bursitis NOS; M726, Necrotising fasciitis; M728, Abscess of fascia; M730, Gonococcal bursitis (A54.4+); M86, Osteomyelitis; M901, Periostitis in other infectious diseases classified elsewhere; M902, Osteopathy in other infectious diseases classified elsewhere; N10, Acute pyelonephritis; N12, Pyelonephritis NOS; N136, Pyonephrosis; N151, Renal and perinephric abscess; N159, Infection of kidney, NOS; N20, Calculous pyelonephritis; N390, Urinary tract infection, site not specified; N41, Inflammatory diseases of prostate; N431, Infected hydrocele; N45, Orchitis and epididymitis; N482, Other inflammatory disorders of penis; N49, Inflammatory disorders of male genital organs, NEC; N510, Gonococcal prostatitis (A54.2+); N511, Chlamydial epididymitis (A56.1+); N512, Balanitis in diseases classified elsewhere; N61, Inflammatory disorders of breast; N70, Salpingitis and oophoritis; N71, Inflammatory disease of uterus, except cervix; N72, Inflammatory disease of cervix uteri; N73, Other female pelvic inflammatory diseases; N74, Female pelvic inflammatory disorders in diseases classified elsewhere; N751, Abscess of Bartholin’s gland; N758, Bartholinitis; N76, Other inflammation of vagina and vulva; N77, Vulvovaginal ulceration and inflammation in diseases classified elsewhere; N82, Fistulae involving female genital tract; N980, Infection associated with artificial insemination; R02, Gangrene, NEC; R091, Pleurisy; R50, Fever of other and unknown origin; R572, Septic shock; R578, Endotoxic shock; R65, Systemic Inflammatory Response Syndrome; T793, Post-traumatic wound infection, NEC; T801, Phlebitis following infusion, transfusion and therapeutic injection; T802, Infections following infusion, transfusion and therapeutic injection; T814, Infection following a procedure, NEC; T826, Infection and inflammatory reaction due to cardiac valve prosthesis; T827, Infection and inflammatory reaction due to other cardiac and vascular devices, implants and grafts; T835, Infection and inflammatory reaction due to prosthetic device, implant and graft in urinary system; T836, Infection and inflammatory reaction due to prosthetic device, implant and graft in genital tract; T845, Infection and inflammatory reaction due to internal joint prosthesis; T846, Infection and inflammatory reaction due to internal fixation device (any site); T847, Infection and inflammatory reaction due to other internal orthopedic prosthetic devices, implants and grafts; T857, Infection and inflammatory reaction due to other internal prosthetic devices, implants and grafts; T874, Infection of amputation stump; T880, Infection following immunization; U80, Agent resistant to penicillin and related antibiotics; U81, Agent resistant to vancomycin and related antibiotics; U88, Agent resistant to multiple antibiotics; U89, Agent resistant to other and unspecified antibiotic
