# Supplementary material for: Vitamin C for ≥ 5 days is associated with decreased hospital mortality in sepsis subgroups: a nationwide cohort study
Source: Crit Care. 2022 Jan 5;26:3. doi: 10.1186/s13054-021-03872-3 (PMC8728994; doi:10.1186/s13054-021-03872-3)
Supplement: Supplementary file 2 — Additional file 2: Table S1. ICD-10-based classification of organ dysfunction. Table S2. Anatomical Therapeutic Chemical codes and charge codes for intravenous vitamin C, corticosteroids, and thiamine. Table S3. ICD-10 codes for different sites of infection. Table S4. Baseline patient characteristics in the unmatched cohort. Table S5. Primary and secondary outcomes according to ICU length of stay. Table S6. Secondary outcomes of patients who survived to hospital discharge. Table S7. Risk factors for hospital mortality. Table S8. Primary and secondary outcomes according to vitamin C duration. Table S9. Primary and secondary outcomes in patients who received vitamin C for ≥ 5 days and in matched controls. Table S10. Primary and secondary outcomes according to ICU length of stay in patients who received vitamin C for ≥ 5 days and in matched controls. Table S11. Primary and secondary outcomes by subpopulations. Table S12. Primary and secondary outcomes by subpopulation in patients who received vitamin C for ≥ 5 days and in matched controls. [file 13054_2021_3872_MOESM2_ESM.docx]

**Table S1** ICD-10-based classification of organ dysfunction

| Variable | Codes |
| --- | --- |
| **Cardiovascular** |  |
| Septic shock | R572 |
| Hypotension | I95 |
| Other hypotension | I958 |
| Hypotension, unspecified | I959 |
| Shock, NEC | R57 |
| Other shock | R578 |
| Shock, unspecified | R579 |
| Shock (endotoxic, hypovolemic) during or following a procedure | T811 |
| Use of vasopressor (norepinephrine, epinephrine, vasopressin, dopamine) |  |
| **Respiratory** |  |
| Adult respiratory distress syndrome | J80 |
| Pulmonary edema | J81 |
| Respiratory failure, NEC | J96 |
| Acute respiratory failure | J960 |
| Respiratory failure, unspecified | J969 |
| Hypoxemia | R0902 |
| Cyanosis | R230 |
| Dependence on respirator | Z991 |
| Conventional oxygen therapy, high-flow nasal cannula, mechanical ventilation |  |
| **Neurologic** |  |
| Delirium, not induced by alcohol and other psychoactive substances | F05 |
| Other mental disorders due to brain damage and dysfunction and to physical disease | F06 |
| Organic psychosis NOS | F09 |
| Anoxic brain damage, NEC | G931 |
| Encephalopathy, unspecified | G934 |
| Metabolic encephalopathy | G9380 |
| Somnolence, stupor and coma | R40 |
| Somnolence | R400 |
| Stupor | R401 |
| Disorientation, unspecified | R410 |
| **Hematologic** |  |
| Disseminated intravascular coagulation (defibrination syndrome) | D65 |
| Other coagulation defects | D68 |
| Other specified coagulation defects | D688 |
| Coagulation defect, unspecified | D689 |
| Purpura and other hemorrhagic conditions | D69 |
| Secondary thrombocytopenia | D695 |
| Thrombocytopenia, unspecified | D696 |
| Spontaneous ecchymoses | R233 |
| Abnormal coagulation lab | R791 |
| **Hepatic** |  |
| Hepatic failure, NEC | K72 |
| Central hemorrhagic necrosis of liver | K762 |
| Infarction of liver | K763 |
| Unspecified jaundice | R17 |
| **Renal** |  |
| Acute renal failure | N17 |
| Unspecified renal failure | N19 |
| Postprocedural renal failure | N990 |
| Anuria and oliguria | R34 |
| Abnormal results of kidney function studies | R944 |
| Dependence on renal dialysis | Z992 |
| Variable | Codes |
| Renal replacement therapy |  |
| **Metabolic** |  |
| Acidosis | E872 |

*ICD* International Classification of Diseases, *NEC* not elsewhere classified, *NOS* not otherwise specified

**Table S2** Anatomical Therapeutic Chemical codes and charge codes for intravenous vitamin C, corticosteroids, and thiamine

| Variable | ATC codes | Charge codes |
| --- | --- | --- |
| **Vitamin C** |  |  |
| Ascorbic acid 100 mg | A11GA01 | 110430BIJ |
| Ascorbic acid 500 mg | A11GA01 | 110431BIJ |
| Ascorbic acid 5 g | A11GA01 | 110432BIJ |
| Ascorbic acid 10 g | A11GA01 | 110433BIJ |
| Ascorbic acid 25 g | A11GA01 | 110435BIJ |
| **Betamethasone** |  |  |
| Betamethasone 4 mg | H02AB01 | 116530BIJ |
| **Dexamethasone** |  |  |
| Dexamethasone palmitate 4 mg | H02AB02 | 142030BIJ |
| Dexamethasone disodium phosphate 4.37 mg | H02AB02 | 142230BIJ |
| Dexamethasone disodium phosphate 5 mg | H02AB02 | 142232BIJ |
| Dexamethasone disodium phosphate 20 mg | H02AB02 | 142233BIJ |
| **Methylprednisolone** |  |  |
| Methylprednisolone acetate 40 mg | H02AB04 | 193530BIJ |
| Methylprednisolone acetate 200 mg | H02AB04 | 193531BIJ |
| Methylprednisolone sodium succinate 125 mg | H02AB04 | 193601BIJ |
| Methylprednisolone sodium succinate 250 mg | H02AB04 | 193602BIJ |
| Methylprednisolone sodium succinate 40 mg | H02AB04 | 193603BIJ |
| Methylprednisolone sodium succinate 500 mg | H02AB04 | 193604BIJ |
| **Prednisolone** |  |  |
| Prednisolone 250 mg | H02AB06 | 217302BIJ |
| **Triamcinolone** |  |  |
| Triamcinolone 40 mg | H02AB08 | 243335BIJ |
| Triamcinolone 50 mg | H02AB08 | 243336BIJ |
| Triamcinolone 200 mg | H02AB08 | 243337BIJ |
| **Hydrocortisone** |  |  |
| Hydrocortisone 100 mg | H02AB09 | 171201BIJ |
| Hydrocortisone 250 mg | H02AB09 | 171202BIJ |
| **Thiamine** |  |  |
| Thiamine 50 mg | A11DA01 | 237330BIJ |

*ATC* Anatomical Therapeutic Chemical

**Table S3** ICD-10 codes for different sites of infection

| Variable | Codes |
| --- | --- |
| Lung | A065, A202, A212, J108, J09, J101, J111, J118, J210, J218, J219, J22, J440, A221, A310, A420, A430, A481, B012, B052, B250, B371, B380, B382, B392, B402, B410, B420, B440, B441, B450, B460, J100, J110, J120, J121, J122, J128, J129, J13, J14, J150, J151, J152, J153, J154, J155, J156, J157, J158, J159, J160, J168, J180, J181, J182, J188, J189, J850, J851, J984 |
| Gastrointestinal tract | A000, A001, A009, A010, A011, A012, A013, A014, A020, A030, A031, A032, A033, A038, A039, A040, A041, A042, A043, A044, A045, A046, A047, A048, A049, A050, A051, A052, A053, A054, A058, A059, A060, A062, A063, A064, A070, A071, A072, A073, A078, A079, A080, A081, A082, A083, A084, A085, A090, A099, A213, A222, A421, B054, B150, B159, B160, B161, B162, B169, B170, B171, B172, B178, B179, B190, B199, B251, B258, B462, K35, K36, K37, K570, K572, K574, K578, K610, K611, K612, K613, K614, K630, K650, K659, K750, K810, K818, K819, K521, K529, K571, K573, K628, K638, K650, K658, K830 |
| Genitourinary tract | N10, N12, N136, N151, N300, N303, N340, N341, N342, N343, N390, N159, N288, N308, N309, B260, B373, B374, N410, N412, N413, N418, N419, N431, N450, N459, N481, N482, N490, N491, N492, N498, N499, N700, N709, N710, N719, N72, N730, N732, N733, N735, N738, N739, N751, N760, N762, N764, N480, N758, N768, N323 |
| Musculoskeletal, skin and soft tissue | B330, M000, M001, M002, M008, M009, M462, M463, M465, M600, M650, M651, M710, M711, M860, M861, M862, M868, M869, M468, M728, M948, A067, A201, A220, A260, A268, A269, A311, A320, A363, A431, A441, A46, A480, B000, B001, B029, B07, B088, B09, B350, B351, B352, B353, B354, B355, B356, B358, B359, B360, B361, B362, B363, B368, B369, B372, B383, B403, B421, B430, B432, B452, B463, B480, L00, L010, L011, L020, L021, L022, L023, L024, L028, L029, L030, L031, L032, L033, L038, L039, L040, L041, L042, L043, L048, L049, L050, L059, L080, L081, L088, L089, L303, L702, R02, L401, L403, L700, L732, L84, L88, L980 |
| Device related | T793, T802, T814, T826, T827, T835, T836, T845, T846, T847, T874, T880, T868, T871, T870, T857 |
| Central nervous system | A066, A203, A321, A390, A83, A858, A86, A870, A871, A872, A878, A879, A888, A89, B003, B004, B010, B011, B020, B021, B022, B050, B051, B060, B261, B262, B375, B384, B431, B451, B461, G000, G001, G002, G003, G008, G009, G040, G042, G060, G061, G062, G937, F059, G039, G048, G629 |
| Endocarditis | B376, I011, I33, I38, I39 |

*ICD* International Classification of Diseases

**Table S4** Baseline patient characteristics in the unmatched cohort

| Characteristics | Vitamin C  (n = 36327) | Control  (n = 347955) | SMD |
| --- | --- | --- | --- |
| Age, mean (SD), y | 70.7 (15.2) | 68.8 (15.4) | 0.12 |
| Sex, No. (%) |  |  | –0.06 |
| Male | 20093 (55) | 197306 (57) |  |
| Female | 16234 (45) | 150649 (43) |  |
| Comorbidities, No. (%) |  |  |  |
| Diabetes | 15369 (42) | 150060 (43) | –0.02 |
| Hypertension | 24236 (67) | 233517 (67) | –0.008 |
| Myocardial infarction | 1224 (3) | 12043 (4) | –0.005 |
| Congestive heart failure | 6154 (17) | 56476 (16) | 0.02 |
| Cerebrovascular disease | 9683 (27) | 82158 (24) | 0.07 |
| Chronic pulmonary disease | 16133 (44) | 143071 (41) | 0.07 |
| Chronic liver disease | 11268 (31) | 99730 (29) | 0.05 |
| Chronic kidney disease | 3929 (11) | 40975 (12) | –0.03 |
| Malignancy | 6965 (19) | 69245 (20) | –0.02 |
| Charlson Comorbidity Index, mean (SD) | 3.0 (1.9) | 2.9 (1.9) | 0.11 |
| Immunosuppression, No. (%)^a^ | 10313 (28) | 100961 (29) | –0.01 |
| Previous steroid use, No. (%)^b^ | 4461 (12) | 44183 (13) | –0.01 |
| Site of infection, No. (%) |  |  |  |
| Lung | 11088 (31) | 81517 (23) | 0.16 |
| Gastrointestinal tract | 4801 (13) | 35295 (10) | 0.10 |
| Genitourinary tract | 4644 (13) | 31449 (9) | 0.12 |
| Hospital capacity, No. (%) |  |  | 0.53 |
| <500 beds | 18373 (51) | 90586 (26) |  |
| 500–1000 beds | 14259 (39) | 197250 (57) |  |
| ≥1000 beds | 3695 (10) | 60119 (17) |  |
| Presented to emergency department, No. (%) | 28337 (78) | 282531 (81) | –0.08 |
| Medical department, No. (%) | 25723 (71) | 240123 (69) | 0.04 |
| Septic shock, No. (%) | 18077 (50) | 191761 (55) | –0.11 |
| Organ dysfunction, No. (%) |  |  |  |
| Cardiovascular | 18325 (50) | 193419 (56) | –0.103 |
| Respiratory | 33080 (91) | 320585 (92) | –0.04 |
| Neurologic | 2008 (6) | 13226 (4) | 0.08 |
| Hematologic | 1032 (3) | 11424 (3) | –0.03 |
| Hepatic | 621 (2) | 4486 (1) | 0.04 |
| Renal | 4801 (13) | 47552 (14) | –0.01 |
| Metabolic | 470 (1) | 4686 (1) | –0.005 |
| No. of organ dysfunctions, No. (%) |  |  | 0.05 |
| 1 | 14955 (41) | 137931 (40) |  |
| 2 | 14870 (41) | 150335 (43) |  |
| 3 | 4290 (12) | 42688 (12) |  |
| ≥4 | 706 (2) | 7572 (2) |  |
| Corticosteroids, No. (%) | 13968 (38) | 121066 (35) | 0.08 |
| Thiamine, No. (%) | 7745 (21) | 34591 (10) | 0.32 |
| Conventional oxygen therapy, No. (%) | 31670 (87) | 297888 (86) | 0.05 |
| High-flow nasal cannula, No. (%) | 4059 (11) | 50101 (14) | –0.10 |
| Mechanical ventilation, No. (%) | 10793 (30) | 123863 (36) | –0.13 |
| Renal replacement therapy, No. (%) | 2628 (7) | 29502 (9) | –0.05 |

*SMD* standardized mean difference

^a^ Immunosuppression included malignancy, human immunodeficiency virus infection, organ transplant, or immunosuppressive therapy

^b^ Defined by oral or intravenous for ≥30 days during the preceding year

**Table S5** Primary and secondary outcomes according to ICU length of stay

| Outcomes | Vitamin C | Control | Difference (95% CI) | *P* value |
| --- | --- | --- | --- | --- |
| **Length of stay ≤5 days**  **(n = 18620/21257)** |  |  |  |  |
| Primary outcome |  |  |  |  |
| Hospital mortality, No. (%) | 2064 (11.1) | 2624 (12.3) | –1.3 (–1.9 to –0.6)^a^ | <0.001 |
| Secondary outcomes |  |  |  |  |
| 90-d mortality, No. (%) | 3346 (18.0) | 4415 (19.4) | –1.4 (–2.2 to –0.6) | <0.001 |
| Vasopressor days, mean (SD) | 2.3 (1.1)  [n = 7871] | 2.3 (1.0)  [n = 8856] | 0.01 (–0.02 to 0.04) | 0.93 |
| Ventilator days, mean (SD) | 2.4 (3.8)  [n = 3618] | 2.1 (2.8)  [n = 3797] | 0.26 (0.2 to 0.3) | <0.001 |
| Length of stay, mean (SD),  days |  |  |  |  |
| ICU | 3.1 (1.1) | 3.0 (1.1) | 0.06 (0.04 to 0.08) | <0.001 |
| Hospital | 17.3 (22.5) | 14.9 (11.9) | 2.5 (2.4 to 2.6) | <0.001 |
| Hospital costs, mean (SD),  U.S. $1000 | 8.3 (9.6) | 8.0 (8.2) | 0.296 (0.29 to 0.3) | 0.001 |
| **Length of stay >5 days**  **(n = 17707/15070)** |  |  |  |  |
| Primary outcome |  |  |  |  |
| Hospital mortality, No. (%) | 4145 (23.4) | 3914 (26.0) | –2.6 (–3.5 to –1.6) | <0.001 |
| Secondary outcomes |  |  |  |  |
| 90-d mortality, No. (%) | 5880 (33.2) | 5705 (37.9) | –4.7 (–5.7 to –3.6) | <0.001 |
| Vasopressor days, mean (SD) | 3.3 (2.9)  [n = 10052] | 3.2 (2.6)  [n = 8585] | 0.1 (0.05 to 0.15) | 0.01 |
| Ventilator days, mean (SD) | 13.1 (22.1)  [n = 7175] | 11.6 (18.8)  [n = 6607] | 1.5 (1.3 to 1.6) | <0.001 |
| Length of stay, mean (SD),  days |  |  |  |  |
| ICU | 18.5 (22.5) | 16.4 (19.5) | 2.1 (2.0 to 2.2) | <0.001 |
| Hospital | 37.3 (53.1) | 29.7 (29.1) | 7.5 (7.4 to 7.7) | <0.001 |
| Hospital costs, mean (SD),  U.S. $1000 | 17.8 (23.6) | 17.1 (36.1) | 0.7 (0.69 to 0.71) | 0.04 |

*ICU* intensive care unit

^a^ The mean of all paired differences in the treatment group minus the control group

**Table S6** Secondary outcomes of patients who survived to hospital discharge

| Outcomes | Vitamin C  (n = 30118) | Control  (n = 29789) | Difference (95% CI) | *P* value |
| --- | --- | --- | --- | --- |
| Vasopressor days, mean (SD) | 2.7 (2.1)  [n = 12863] | 2.6 (1.8)  [n = 12316] | 0.1 (0.07 to 0.14)^a^ | <0.001 |
| Ventilator days, mean (SD) | 8.0 (15.2)  [n = 7155] | 6.9 (13.7)  [n = 6747] | 1.0 (0.9 to 1.2) | <0.001 |
| Length of stay, mean (SD), days |  |  |  |  |
| ICU | 9.3 (14.8) | 7.7 (12.3) | 1.62 (1.6 to 1.7) | <0.001 |
| Hospital | 25.9 (38.8) | 20.7 (20.2) | 5.2 (5.1 to 5.3) | <0.001 |
| Hospital costs, mean (SD),  U.S. $1000 | 11.9 (16.2) | 11.2 (13.8) | 0.72 (0.71 to 0.73) | <0.001 |

*ICU* intensive care unit

^a^ The mean of all paired differences in the treatment group minus the control group

**Table S7** Risk factors for hospital mortality^a^

| Variable | No. of deaths/patients (%) | PS-matched OR (95% CI) |
| --- | --- | --- |
| Age, y |  |  |
| 18–65 | 2706/23527 (11.5) | 1 (reference) |
| 66–79 | 4231/24056 (17.6) | 1.45 (1.34 to 1.57) |
| ≥80 | 5810/25071 (23.2) | 1.99 (1.84 to 2.15) |
| Sex |  |  |
| Male | 7422/40012 (18.6) | 1 (reference) |
| Female | 5325/32642 (16.3) | 0.82 (0.77 to 0.87) |
| Immunosuppression^b^ | 4906/20173 (24.3) | 1.61 (1.51 to 1.71) |
| Charlson Comorbidity Index |  |  |
| 0–1 | 2041/16629 (12.3) | 1 (reference) |
| 2–3 | 4953/29587 (16.7) | 1.20 (1.10 to 1.32) |
| ≥4 | 5753/26438 (21.8) | 1.60 (1.46 to 1.76) |
| Previous steroid use^c^ | 2163/8677 (24.9) | 1.41 (1.30 to 1.52) |
| Site of infection |  |  |
| Lung | 5910/22007 (26.9) | 2.31 (2.15 to 2.47) |
| Gastrointestinal tract | 1559/9371 (16.6) | 0.85 (0.78 to 0.93) |
| Genitourinary tract | 1381/9162 (15.1) | 0.72 (0.66 to 0.79) |
| Hospital capacity |  |  |
| <500 beds | 6909/36850 (18.8) | 1 (reference) |
| 500–1000 beds | 4479/28805 (15.6) | 0.57 (0.48 to 0.67) |
| ≥1000 beds | 1359/6999 (19.4) | 0.56 (0.46 to 0.68) |
| Presented to emergency department | 9912/56939 (17.4) | 1.05 (0.98 to 1.12) |
| Medical department | 9760/51178 (19.1) | 1.30 (1.22 to 1.40) |
| Septic shock | 10226/35646 (28.7) | 5.73 (5.28 to 6.23) |
| No. of organ dysfunctions |  |  |
| 1 | 2002/30753 (6.5) | 1 (reference) |
| 2 | 6201/29386 (21.1) | 3.84 (3.50 to 4.21) |
| 3 | 3663/8166 (44.9) | 10.69 (9.51 to 12.02) |
| ≥4 | 860/1425 (60.4) | 22.87 (18.02 to 29.02) |
| Corticosteroids | 6508/27727 (23.5) | 1.94 (1.82 to 2.07) |
| Thiamine | 2229/11262 (19.8) | 1.19 (0.10 to 1.28) |
| Conventional oxygen therapy | 10823/63528 (17.0) | 0.89 (0.82 to 0.97) |
| High-flow nasal cannula | 2755/7921 (34.8) | 2.57 (2.36 to 2.81) |
| Mechanical ventilation | 7295/21197 (34.4) | 5.03 (4.65 to 5.45) |
| Renal replacement therapy | 2938/5122 (57.4) | 7.94 (7.00 to 9.01) |
| Vitamin C | 6209/36327 (17.1) | 0.94 (0.90 to 0.97) |

^a^ The numbers and percentages of patients who died according to each risk factor are shown. The odds ratios (ORs) and 95% confidence intervals (CIs) are calculated in the propensity score (PS)-matched treatment and control groups

^b^ Immunosuppression included malignancy, human immunodeficiency virus infection, organ transplant, or immunosuppressive therapy

^c^ Defined by oral or intravenous for ≥30 days during the preceding year

**Table S8** Primary and secondary outcomes according to vitamin C duration

| Outcomes | 1–2 days  (n = 10826) | 3–4 days  (n = 5823) | ≥5 days  (n = 19678) | *P* value |
| --- | --- | --- | --- | --- |
| **Primary outcome** |  |  |  |  |
| Hospital mortality, No. (%) | 2032 (18.8) | 1068 (18.3) | 3109 (15.8) | <0.001 |
| **Secondary outcomes** |  |  |  |  |
| 90-d mortality, No. (%) | 2952 (27.3) | 1571 (27.0) | 4703 (23.9) | <0.001 |
| Vasopressor days, mean (SD) | 2.7 (2.0)  [n = 6136] | 2.8 (2.1)  [n = 2827] | 3.0 (2.6)  [n = 8960] | <0.001 |
| Ventilator days, mean (SD) | 7.0 (12.3)  [n = 3919] | 9.2 (19.8)  [n = 1668] | 11.5 (22.1)  [n = 5206] | <0.001 |
| Length of stay, mean (SD), days |  |  |  |  |
| ICU | 7.8 (12.2) | 7.9 (14.2) | 12.9 (20.3) | <0.001 |
| Hospital | 21.7 (28.1) | 19.8 (28.9) | 32.1 (49.6) | <0.001 |
| Hospital costs, mean (SD),  U.S. $1000 | 13.8 (17.6) | 11.7 (17.0) | 13.5 (20.3) | 0.67 |

*ICU* intensive care unit

**Table S9** Primary and secondary outcomes in patients who received vitamin C for ≥5 days and in matched controls

| Outcomes | Vitamin C  (n = 19678) | Control  (n = 19678) | Difference (95% CI) | *P* value |
| --- | --- | --- | --- | --- |
| **Primary outcome** |  |  |  |  |
| Hospital mortality, No. (%) | 3109 (15.8) | 3491 (17.7) | –1.9 (–2.5 to –1.4)^a^ | <0.001 |
| **Secondary outcomes** |  |  |  |  |
| 90-d mortality, No. (%) | 4703 (23.9) | 5338 (27.1) | –3.2 (–3.8 to –2.6) | <0.001 |
| Vasopressor days,  mean (SD) | 3.0 (2.6)  [n = 8960] | 2.8 (2.1)  [n = 9015] | 0.24 (0.2 to 0.3) | <0.001 |
| Ventilator days, mean (SD) | 11.5 (22.1)  [n = 5206] | 8.3 (13.6)  [n = 5234] | 3.2 (3.0 to 3.4) | <0.001 |
| Length of stay, mean (SD),  days |  |  |  |  |
| ICU | 12.9 (20.3) | 8.7 (13.3) | 4.2 (4.1 to 4.3) | <0.001 |
| Hospital | 32.1 (49.6) | 21.2 (21.4) | 10.9 (10.8 to 11.0) | <0.001 |
| Hospital costs, mean (SD),  U.S. $1000 | 13.5 (20.3) | 11.5 (14.6) | 2.0 (1.99 to 2.01) | <0.001 |

*ICU* intensive care unit

^a^ The mean of all paired differences in the treatment group minus the control group

**Table S10** Primary and secondary outcomes according to ICU length of stay in patients who received vitamin C for ≥5 days and in matched controls

| Outcomes | Vitamin C | Control | Difference (95% CI) | *P* value |
| --- | --- | --- | --- | --- |
| **Length of stay ≤5 days**  **(n = 7899/11319)** |  |  |  |  |
| Primary outcome |  |  |  |  |
| Hospital mortality, No. (%) | 541 (6.8) | 1378 (12.2) | –5.3 (–6.1 to –4.5)^a^ | <0.001 |
| Secondary outcomes |  |  |  |  |
| 90-d mortality, No. (%) | 1046 (13.2) | 2226 (19.7) | –6.4 (–7.5 to –5.4) | <0.001 |
| Vasopressor days, mean (SD) | 2.4 (1.2)  [n = 2782] | 2.3 (1.1)  [n = 4424] | 0.02 (–0.03 to 0.1) | 0.38 |
| Ventilator days, mean (SD) | 3.0 (5.1)  [n = 1033] | 2.1 (2.8)  [n = 1835] | 0.9 (0.7 to 1.1) | <0.001 |
| Length of stay, mean (SD),  days |  |  |  |  |
| ICU | 3.2 (1.1) | 3.0 (1.1) | 0.2 (0.18 to 0.24) | <0.001 |
| Hospital | 21.1 (27.6) | 15.1 (11.8) | 6.0 (5.9 to 6.1) | <0.001 |
| Hospital costs, mean (SD),  U.S. $1000 | 7.9 (9.2) | 7.6 (7.5) | 0.33 (0.32 to 0.34) | 0.009 |
| **Length of stay >5 days**  **(n = 11779/8359)** |  |  |  |  |
| Primary outcome |  |  |  |  |
| Hospital mortality, No. (%) | 2568 (21.8) | 2113 (25.3) | –3.5 (–4.7 to –2.3) | <0.001 |
| Secondary outcomes |  |  |  |  |
| 90-d mortality, No. (%) | 3657 (31.0) | 3112 (37.2) | –6.2 (–7.5 to –4.9) | <0.001 |
| Vasopressor days, mean (SD) | 3.3 (2.9)  [n = 6178] | 3.1 (2.7)  [n = 4591] | 0.13 (0.1 to 0.2) | 0.02 |
| Ventilator days, mean (SD) | 13.6 (24.0)  [n = 4173] | 11.7 (17.1)  [n = 3399] | 2.0 (1.7 to 2.2) | <0.001 |
| Length of stay, mean (SD),  days |  |  |  |  |
| ICU | 19.4 (24.2) | 16.4 (17.7) | 3.0 (2.9 to 3.1) | <0.001 |
| Hospital | 39.5 (58.9) | 29.5 (27.7) | 10.0 (9.8 to 10.2) | <0.001 |
| Hospital costs, mean (SD),  U.S. $1000 | 16.5 (23.6) | 15.9 (19.2) | 0.3 (–1.2 to 0.2) | 0.06 |

*ICU* intensive care unit

^a^ The mean of all paired differences in the treatment group minus the control group

**Table S11** Primary and secondary outcomes by subpopulations

| Outcomes | Vitamin C | Control | Difference (95% CI) | *P* value |
| --- | --- | --- | --- | --- |
| **Age ≥70 years**  **(n = 21565/21440)** |  |  |  |  |
| Primary outcome |  |  |  |  |
| Hospital mortality, No. (%) | 4357 (20.2) | 4713 (22.0) | –1.8 (–2.3 to –1.2)^a^ | <0.001 |
| Secondary outcomes |  |  |  |  |
| 90-d mortality, No. (%) | 6777 (31.4) | 7430 (34.7) | –3.2 (–4.0 to –2.6) | <0.001 |
| Vasopressor days, mean (SD) | 2.9 (2.3)  [n = 10272] | 2.8 (2.1)  [n = 9976] | 0.12 (0.1 to 0.2) | <0.001 |
| Ventilator days, mean (SD) | 9.8 (20.3)  [n = 5691] | 8.6 (14.2)  [n = 5445] | 1.2 (1.1 to 1.4) | <0.001 |
| Length of stay, mean (SD),  days |  |  |  |  |
| ICU | 11.1 (17.8) | 8.8 (13.1) | 2.2 (2.1 to 2.3) | <0.001 |
| Hospital | 27.5 (43.1) | 20.8 (21.0) | 6.6 (6.5 to 6.7) | <0.001 |
| Hospital costs, mean (SD),  U.S. $1000 | 13.1 (17.4) | 11.9 (14.0) | 1.0 (0.99 to 1.01) | <0.001 |
| **Age <70 years**  **(n = 14762/14887)** |  |  |  |  |
| Primary outcome |  |  |  |  |
| Hospital mortality, No. (%) | 1852 (12.6) | 1825 (12.3) | 0.3 (–0.3 to 0.8) | 0.45 |
| Secondary outcomes |  |  |  |  |
| 90-d mortality, No. (%) | 2449 (16.6) | 2390 (16.1) | 0.5 (–0.1 to 1.1) | 0.21 |
| Vasopressor days, mean (SD) | 2.8 (2.3)  [n = 7651] | 2.7 (1.9)  [n = 7465] | 0.11 (0.1 to 0.2) | <0.001 |
| Ventilator days, mean (SD) | 9.2 (17.0)  [n = 5102] | 7.7 (17.2)  [n = 4959] | 1.5 (1.3 to 1.7) | 0.44 |
| Length of stay, mean (SD),  days |  |  |  |  |
| ICU | 9.9 (17.2) | 8.2 (15.7) | 1.74 (1.7 to 1.8) | <0.001 |
| Hospital | 26.5 (39.4) | 21.3 (23.6) | 5.1 (5.0 to 5.3) | <0.001 |
| Hospital costs, mean (SD),  U.S. $1000 | 18.8 (27.0) | 17.0 (42.0) | 1.5 (1.49 to 1.51) | <0.001 |
| **Male (n = 20093/19919)** |  |  |  |  |
| Primary outcome |  |  |  |  |
| Hospital mortality, No. (%) | 3632 (18.1) | 3790 (19.0) | –1.0 (–1.5 to –0.4) | 0.01 |
| Secondary outcomes |  |  |  |  |
| 90-d mortality, No. (%) | 5226 (26.0) | 5570 (28.0) | –2.0 (–2.6 to –1.4) | <0.001 |
| Vasopressor days, mean (SD) | 2.9 (2.4)  [n = 10243] | 2.8 (2.1)  [n = 9930] | 0.12 (0.1 to 0.2) | <0.001 |
| Ventilator days, mean (SD) | 9.9 (19.6)  [n = 6545] | 8.3 (15.5)  [n = 6369] | 1.6 (1.5 to 1.8) | <0.001 |
| Length of stay, mean (SD),  days |  |  |  |  |
| ICU | 11.3 (19.5) | 9.0 (14.7) | 2.3 (2.2 to 2.4) | <0.001 |
| Hospital | 27.7 (42.1) | 21.5 (22.9) | 6.2 (6.1 to 6.3) | <0.001 |
| Hospital costs, mean (SD),  U.S. $1000 | 16.5 (23.3) | 15.1 (36.5) | 1.26 (1.25 to 1.3) | <0.001 |
| **Female (n = 16234/16408)** |  |  |  |  |
| Primary outcome |  |  |  |  |
| Hospital mortality, No. (%) | 2577 (15.9) | 2748 (16.8) | –0.9 (–1.4 to –0.3) | 0.03 |
| Secondary outcomes |  |  |  |  |
| 90-d mortality, No. (%) | 4000 (24.6) | 4250 (25.9) | –1.3 (–1.9 to –0.6) | 0.009 |
| Vasopressor days, mean (SD) | 2.8 (2.2)  [n = 7680] | 2.7 (1.9)  [n = 7511] | 0.12 (0.1 to 0.2) | <0.001 |
| Outcomes | Vitamin C | Control | Difference (95% CI) | *P* value |
| Ventilator days, mean (SD) | 8.9 (17.6)  [n = 4248] | 7.9 (16.1)  [n = 4035] | 1.0 (0.8 to 1.2) | 0.008 |
| Length of stay, mean (SD),  days |  |  |  |  |
| ICU | 9.8 (14.7) | 8.1 (13.6) | 1.64 (1.6 to 1.7) | <0.001 |
| Hospital | 26.2 (41.0) | 20.4 (21.1) | 5.8 (5.7 to 5.9) | <0.001 |
| Hospital costs, mean (SD),  U.S. $1000 | 13.9 (20.2) | 12.7 (16.2) | 1.08 (1.07 to 1.1) | <0.001 |
| **CCI ≥3 (n = 20798/20271)** |  |  |  |  |
| Primary outcome |  |  |  |  |
| Hospital mortality, No. (%) | 4084 (19.6) | 4287 (21.2) | –1.5 (–2.1 to –1.0) | <0.001 |
| Secondary outcomes |  |  |  |  |
| 90-d mortality, No. (%) | 6137 (29.5) | 6512 (32.1) | –2.6 (–3.2 to –2.0) | <0.001 |
| Vasopressor days, mean (SD) | 2.9 (2.4)  [n = 10552] | 2.7 (2.0)  [n = 10113] | 0.15 (0.1 to 0.2) | <0.001 |
| Ventilator days, mean (SD) | 9.6 (18.8)  [n = 6162] | 8.3 (16.2)  [n = 5812] | 1.3 (1.1 to 1.4) | <0.001 |
| Length of stay, mean (SD),  days |  |  |  |  |
| ICU | 10.7 (16.9) | 8.6 (14.4) | 2.1 (2.0 to 2.2) | <0.001 |
| Hospital | 27.8 (42.8) | 21.4 (22.8) | 6.5 (6.3 to 6.6) | <0.001 |
| Hospital costs, mean (SD),  U.S. $1000 | 15.6 (22.4) | 14.0 (18.3) | 1.35 (1.34 to 1.4) | <0.001 |
| **CCI <3 (n = 15529/16056)** |  |  |  |  |
| Primary outcome |  |  |  |  |
| Hospital mortality, No. (%) | 2125 (13.7) | 2251 (14.0) | –0.3 (–0.9 to 0.2) | 0.39 |
| Secondary outcomes |  |  |  |  |
| 90-d mortality, No. (%) | 3089 (19.9) | 3308 (20.6) | –0.7 (–1.3 to –0.1) | 0.12 |
| Vasopressor days, mean (SD) | 2.8 (2.2)  [n = 7371] | 2.7 (2.0)  [n = 7328] | 0.07 (0.02 to 0.1) | 0.04 |
| Ventilator days, mean (SD) | 9.5 (18.9)  [n = 4631] | 8.0 (15.1)  [n = 4529] | 1.5 (1.3 to 1.6) | <0.001 |
| Length of stay, mean (SD),  days |  |  |  |  |
| ICU | 10.5 (18.3) | 8.6 (14.0) | 1.9 (1.8 to 2.0) | <0.001 |
| Hospital | 26.0 (40.0) | 20.6 (21.2) | 5.4 (5.3 to 5.5) | <0.001 |
| Hospital costs, mean (SD),  U.S. $1000 | 15.1 (21.4) | 14.0 (38.7) | 0.97 (0.96 to 1.0) | <0.001 |
| **Pneumonia (n = 11088/10919)** |  |  |  |  |
| Primary outcome |  |  |  |  |
| Hospital mortality, No. (%) | 2815 (25.4) | 3095 (28.4) | –3.0 (–3.8 to –2.1) | <0.001 |
| Secondary outcomes |  |  |  |  |
| 90-d mortality, No. (%) | 4132 (37.3) | 4343 (39.8) | –2.5 (–3.4 to –1.6) | <0.001 |
| Vasopressor days, mean (SD) | 3.2 (2.8)  [n = 5521] | 3.0 (2.5)  [n = 5526] | 0.17 (0.1 to 0.2) | <0.001 |
| Ventilator days, mean (SD) | 13.5 (25.0)  [n = 3787] | 11.3 (21.1)  [n = 3774] | 2.2 (2.0 to 2.4) | <0.001 |
| Length of stay, mean (SD),  days |  |  |  |  |
| ICU | 15.5 (24.8) | 12.1 (19.2) | 3.4 (3.3 to 3.6) | <0.001 |
| Hospital | 34.4 (58.0) | 24.3 (27.2) | 10.1 (9.9 to 10.2) | <0.001 |
| Hospital costs, mean (SD),  U.S. $1000 | 13.8 (22.4) | 12.1 (16.9) | 1.6 (1.1 to 2.1) | <0.001 |
| **Gastrointestinal**  **(n = 4801/4570)** |  |  |  |  |
| Outcomes | Vitamin C | Control | Difference (95% CI) | *P* value |
| Primary outcome |  |  |  |  |
| Hospital mortality, No. (%) | 805 (16.8) | 754 (16.5) | 0.3 (–0.8 to 1.3) | 0.73 |
| Secondary outcomes |  |  |  |  |
| 90-d mortality, No. (%) | 1181 (24.6) | 1193 (26.1) | –1.5 (–2.7 to –0.3) | 0.09 |
| Vasopressor days, mean (SD) | 2.9 (2.4)  [n = 2434] | 2.7 (2.0)  [n = 2286] | 0.2 (0.1 to 0.3) | 0.002 |
| Ventilator days, mean (SD) | 11.0 (22.7)  [n = 1163] | 8.9 (14.9)  [n = 1031] | 2.1 (1.8 to 2.5) | 0.009 |
| Length of stay, mean (SD),  days |  |  |  |  |
| ICU | 12.1 (23.0) | 8.9 (13.6) | 3.2 (3.0 to 3.4) | <0.001 |
| Hospital | 36.5 (69.7) | 23.0 (23.2) | 13.5 (13.2 to 13.9) | <0.001 |
| Hospital costs, mean (SD),  U.S. $1000 | 12.9 (17.8) | 11.7 (16.0) | 1.3 (0.6 to 2.0) | <0.001 |
| **Genitourinary (n = 4644/4518)** |  |  |  |  |
| Primary outcome |  |  |  |  |
| Hospital mortality, No. (%) | 653 (14.1) | 728 (16.1) | –2.1 (–3.1 to –1.1) | 0.006 |
| Secondary outcomes |  |  |  |  |
| 90-d mortality, No. (%) | 1119 (24.1) | 1268 (28.1) | –4.0 (–5.2 to –2.7) | <0.001 |
| Vasopressor days, mean (SD) | 3.0 (2.5)  [n = 2080] | 2.7 (1.8)  [n = 2077] | 0.2 (0.1 to 0.3) | <0.001 |
| Ventilator days, mean (SD) | 11.6 (25.4)  [n = 751] | 11.9 (29.1)  [n = 728] | –0.4 (–0.9 to 0.2) | 0.80 |
| Length of stay, mean (SD),  days |  |  |  |  |
| ICU | 12.8 (22.7) | 10.1 (16.8) | 2.7 (2.5 to 2.8) | <0.001 |
| Hospital | 36.7 (71.2) | 23.4 (26.4) | 13.3 (13.0 to 13.5) | <0.001 |
| Hospital costs, mean (SD),  U.S. $1000 | 9.8 (12.5) | 9.4 (12.3) | 0.4 (–0.1 to 0.9) | 0.15 |
| **Septic shock (n = 18077/17569)** |  |  |  |  |
| Primary outcome |  |  |  |  |
| Hospital mortality, No. (%) | 5082 (28.1) | 5144 (29.3) | –1.2 (–1.8 to –0.5) | 0.02 |
| Secondary outcomes |  |  |  |  |
| 90-d mortality, No. (%) | 6247 (34.6) | 6504 (37.0) | –2.5 (–3.2 to –1.8) | <0.001 |
| Vasopressor days, mean (SD) | 2.9 (2.3) | 2.7 (2.0) | 0.12 (0.1 to 0.2) | <0.001 |
| Ventilator days, mean (SD) | 10.2 (19.8)  [n = 9233] | 8.8 (16.8)  [n = 8603] | 1.4 (1.3 to 1.5) | <0.001 |
| Length of stay, mean (SD),  days |  |  |  |  |
| ICU | 13.4 (21.5) | 10.8 (18.5) | 2.6 (2.5 to 2.7) | <0.001 |
| Hospital | 31.7 (45.1) | 24.8 (27.7) | 6.9 (6.8 to 7.0) | <0.001 |
| Hospital costs, mean (SD),  U.S. $1000 | 18.3 (24.6) | 17.3 (34.9) | 2.3 (1.7 to 2.9) | <0.001 |
| **Mechanical ventilation**  **(n = 10793/10404)** |  |  |  |  |
| Primary outcome |  |  |  |  |
| Hospital mortality, No. (%) | 3638 (33.7) | 3657 (35.2) | –1.4 (–2.3 to –0.6) | 0.03 |
| Secondary outcomes |  |  |  |  |
| 90-d mortality, No. (%) | 4277 (39.6) | 4426 (42.5) | –2.9 (–3.8 to –2.0) | <0.001 |
| Vasopressor days, mean (SD) | 3.0 (2.7)  [n = 9219] | 2.9 (2.3)  [n = 8589] | 0.2 (0.03 to 0.5) | <0.001 |
| Ventilator days, mean (SD) | 9.5 (18.8) | 8.2 (15.7) | 1.31 (1.3 to 1.4) | <0.001 |
| Length of stay, mean (SD),  days |  |  |  |  |
| Outcomes | Vitamin C | Control | Difference (95% CI) | *P* value |
| ICU | 17.1 (26.4) | 14.6 (22.6) | 2.6 (2.4 to 2.7) | <0.001 |
| Hospital | 34.6 (44.8) | 29.1 (32.6) | 5.5 (5.4 to 5.7) | <0.001 |
| Hospital costs, mean (SD),  U.S. $1000 | 26.3 (29.2) | 23.1 (43.9) | 3.2 (2.2 to 4.2) | <0.001 |
| **Renal replacement therapy**  **(n = 2628/2494)** |  |  |  |  |
| Primary outcome |  |  |  |  |
| Hospital mortality, No. (%) | 1501 (57.1) | 1437 (57.6) | –0.5 (–2.4 to 1.4) | 0.72 |
| Secondary outcomes |  |  |  |  |
| 90-d mortality, No. (%) | 1575 (59.9) | 1567 (62.8) | –2.9 (–4.8 to –1.0) | 0.03 |
| Vasopressor days, mean (SD) | 3.3 (3.0)  [n = 2427] | 3.1 (2.6)  [n = 2198] | 0.2 (0.1 to 0.3) | 0.04 |
| Ventilator days, mean (SD) | 13.4 (21.2)  [n = 2037] | 11.0 (16.0)  [n = 1704] | 2.4 (2.1 to 2.7) | <0.001 |
| Length of stay, mean (SD),  days |  |  |  |  |
| ICU | 17.8 (24.0) | 14.1 (22.7) | 3.6 (3.4 to 3.9) | <0.001 |
| Hospital | 36.3 (46.0) | 29.4 (37.6) | 6.9 (6.5 to 7.2) | <0.001 |
| Hospital costs, mean (SD),  U.S. $1000 | 38.6 (43.2) | 29.4 (34.0) | 9.1 (7.0 to 11.2) | <0.001 |

*CCI* Charlson Comorbidity Index, *ICU* intensive care unit

^a^ The mean of all paired differences in the treatment group minus the control group

**Table S12** Primary and secondary outcomes by subpopulation in patients who received vitamin C for ≥5 days and in matched controls

| Outcomes | Vitamin C | Control | Difference (95% CI) | *P* value |
| --- | --- | --- | --- | --- |
| **Age ≥70 years**  **(n = 12088/11877)** |  |  |  |  |
| Primary outcome |  |  |  |  |
| Hospital mortality, No. (%) | 2247 (18.6) | 2596 (21.9) | –3.3 (–4.0 to –2.6)^a^ | <0.001 |
| Secondary outcomes |  |  |  |  |
| 90-d mortality, No. (%) | 3579 (29.6) | 4145 (34.9) | –5.3 (–6.1 to –4.5) | <0.001 |
| Vasopressor days,  mean (SD) | 3.0 (2.6)  [n = 5313] | 2.8 (2.1)  [n = 5254] | 0.24 (0.2 to 0.3) | <0.001 |
| Ventilator days, mean (SD) | 11.7 (23.2)  [n = 2714] | 8.9 (15.7)  [n = 2783] | 2.8 (2.6 to 3.1) | <0.001 |
| Length of stay, mean (SD),  days |  |  |  |  |
| ICU | 13.2 (19.9) | 9.1 (14.1) | 4.1 (4.0 to 4.2) | <0.001 |
| Hospital | 32.5 (51.5) | 21.1 (21.6) | 11.4 (11.3 to 11.6) | <0.001 |
| Hospital costs, mean (SD),  U.S. $1000 | 13.0 (17.3) | 11.4 (13.7) | 1.39 (1.38 to 1.4) | <0.001 |
| **Age <70 years**  **(n = 7590/7801)** |  |  |  |  |
| Primary outcome |  |  |  |  |
| Hospital mortality, No. (%) | 862 (11.4) | 895 (11.5) | –0.1 (–0.8 to 0.6) | 0.82 |
| Secondary outcomes |  |  |  |  |
| 90-d mortality, No. (%) | 1124 (14.8) | 1193 (15.3) | –0.5 (–1.3 to 0.3) | 0.40 |
| Vasopressor days,  mean (SD) | 2.9 (2.6)  [n = 3647] | 2.7 (2.0)  [n = 3761] | 0.23 (0.2 to 0.3) | <0.001 |
| Ventilator days, mean (SD) | 11.3 (20.7)  [n = 2492] | 7.7 (13.4)  [n = 2451] | 3.6 (3.4 to 3.9) | <0.001 |
| Length of stay, mean (SD),  days |  |  |  |  |
| ICU | 12.5 (21.0) | 8.2 (12.1) | 4.3 (4.2 to 4.5) | <0.001 |
| Hospital | 31.5 (46.4) | 21.5 (21.0) | 10.1 (9.9 to 10.2) | <0.001 |
| Hospital costs, mean (SD),  U.S. $1000 | 19.7 (30.3) | 16.1 (20.6) | 3.09 (3.08 to 3.1) | <0.001 |
| **Male (n = 10897/10716)** |  |  |  |  |
| Primary outcome |  |  |  |  |
| Hospital mortality, No. (%) | 1853 (17.0) | 1970 (18.4) | –1.4 (–2.1 to –0.7) | 0.008 |
| Secondary outcomes |  |  |  |  |
| 90-d mortality, No. (%) | 2672 (24.5) | 2952 (27.5) | –4.2 (–5.1 to –3.4) | <0.001 |
| Vasopressor days,  mean (SD) | 3.0 (2.7)  [n = 5123] | 2.8 (2.0)  [n = 5074] | 0.27 (0.2 to 0.3) | <0.001 |
| Ventilator days, mean (SD) | 12.1 (23.2)  [n = 3199] | 8.5 (15.2)  [n = 3181] | 3.6 (3.4 to 3.8) | <0.001 |
| Length of stay, mean (SD),  days |  |  |  |  |
| ICU | 13.9 (23.2) | 9.1 (14.2) | 4.8 (4.6 to 4.9) | <0.001 |
| Hospital | 33.0 (50.1) | 21.7 (22.3) | 11.2 (11.1 to 11.4) | <0.001 |
| Hospital costs, mean (SD),  U.S. $1000 | 16.8 (24.8) | 14.2 (18.1) | 2.27 (2.26 to 2.28) | <0.001 |
| **Female (n = 8781/8962)** |  |  |  |  |
| Primary outcome |  |  |  |  |
| Hospital mortality, No. (%) | 1256 (14.3) | 1521 (17.0) | –2.7 (–3.4 to –1.9) | <0.001 |
| Secondary outcomes |  |  |  |  |
| 90-d mortality, No. (%) | 2031 (23.1) | 2386 (26.6) | –2.0 (–2.8 to –1.1) | <0.001 |
| Outcomes | Vitamin C | Control | Difference (95% CI) | *P* value |
| Vasopressor days,  mean (SD) | 2.9 (2.5)  [n = 3837] | 2.8 (2.1)  [n = 3941] | 0.2 (0.1 to 0.3) | <0.001 |
| Ventilator days, mean (SD) | 10.6 (20.0)  [n = 2007] | 8.1 (13.7)  [n = 2053] | 2.6 (2.3 to 2.8) | <0.001 |
| Length of stay, mean (SD),  days |  |  |  |  |
| ICU | 11.7 (15.9) | 8.2 (12.2) | 3.5 (3.4 to 3.6) | <0.001 |
| Hospital | 31.1 (49.0) | 20.7 (20.2) | 10.4 (10.3 to 10.6) | <0.001 |
| Hospital costs, mean (SD),  U.S. $1000 | 14.0 (21.5) | 12.1 (15.3) | 1.63 (1.62 to 1.64) | <0.001 |
| **CCI ≥3 (n = 10964/10989)** |  |  |  |  |
| Primary outcome |  |  |  |  |
| Hospital mortality, No. (%) | 2078 (19.0) | 2331 (21.2) | –2.3 (–3.0 to –1.5) | <0.001 |
| Secondary outcomes |  |  |  |  |
| 90-d mortality, No. (%) | 3119 (28.4) | 3589 (32.7) | –3.0 (–3.8 to –2.2) | <0.001 |
| Vasopressor days,  mean (SD) | 3.1 (2.7)  [n = 5114] | 2.8 (2.1)  [n = 5215] | 0.28 (0.2 to 0.3) | <0.001 |
| Ventilator days, mean (SD) | 11.7 (23.4)  [n = 2820] | 8.6 (16.0)  [n = 2902] | 3.1 (2.8 to 3.3) | <0.001 |
| Length of stay, mean (SD),  days |  |  |  |  |
| ICU | 13.2 (19.9) | 8.8 (14.1) | 4.4 (4.3 to 4.5) | <0.001 |
| Hospital | 33.4 (52.2) | 21.5 (22.0) | 12.0 (11.8 to 12.1) | <0.001 |
| Hospital costs, mean (SD),  U.S. $1000 | 15.5 (23.8) | 13.2 (17.0) | 2.03 (2.02 to 2.04) | <0.001 |
| **CCI <3 (n = 8714/8689)** |  |  |  |  |
| Primary outcome |  |  |  |  |
| Hospital mortality, No. (%) | 1031 (11.8) | 1160 (13.4) | –1.5 (–2.2 to –0.8) | 0.003 |
| Secondary outcomes |  |  |  |  |
| 90-d mortality, No. (%) | 1584 (18.2) | 1749 (20.1) | –3.5 (–4.4 to –2.6) | 0.001 |
| Vasopressor days,  mean (SD) | 2.9 (2.4)  [n = 3846] | 2.7 (2.0)  [n = 3800] | 0.2 (0.1 to 0.3) | <0.001 |
| Ventilator days, mean (SD) | 11.3 (20.4)  [n = 2386] | 8.0 (12.8)  [n = 2332] | 3.4 (3.1 to 3.6) | <0.001 |
| Length of stay,  mean (SD), days |  |  |  |  |
| ICU | 12.6 (20.9) | 8.6 (12.3) | 4.0 (3.9 to 4.1) | <0.001 |
| Hospital | 30.5 (46.2) | 20.9 (20.6) | 9.6 (9.4 to 9.7) | <0.001 |
| Hospital costs, mean (SD),  U.S. $1000 | 15.6 (23.0) | 13.4 (16.8) | 1.97 (1.96 to 1.98) | <0.001 |
| **Pneumonia (n = 6503/6259)** |  |  |  |  |
| Primary outcome |  |  |  |  |
| Hospital mortality, No. (%) | 1537 (23.6) | 1706 (27.3) | –3.6 (–4.7 to –2.6) | <0.001 |
| Secondary outcomes |  |  |  |  |
| 90-d mortality, No. (%) | 2267 (34.9) | 2622 (41.9) | –7.0 (–8.2 to –5.9) | <0.001 |
| Vasopressor days,  mean (SD) | 3.4 (3.1)  [n = 3010] | 3.1 (2.6)  [n = 2998] | 0.34 (0.3 to 0.4) | <0.001 |
| Ventilator days, mean (SD) | 15.3 (29.3)  [n = 2015] | 11.2 (18.8)  [n = 1975] | 4.1 (3.8 to 4.4) | <0.001 |
| Length of stay, mean (SD),  days |  |  |  |  |
| ICU | 18.3 (28.5) | 12.1 (17.2) | 6.3 (6.1 to 6.4) | <0.001 |
| Hospital | 40.5 (68.4) | 24.1 (25.7) | 16.4 (16.2 to 16.6) | <0.001 |
| Hospital costs, mean (SD),  U.S. $1000 | 14.2 (24.8) | 11.3 (15.7) | 2.9 (2.2 to 3.6) | <0.001 |
| Outcomes | Vitamin C | Control | Difference (95% CI) | *P* value |
| **Gastrointestinal**  **(n = 2592/2568)** |  |  |  |  |
| Primary outcome |  |  |  |  |
| Hospital mortality, No. (%) | 436 (16.8) | 423 (16.5) | 0.3 (–1.7 to 2.3) | 0.74 |
| Secondary outcomes |  |  |  |  |
| 90-d mortality, No. (%) | 575 (22.2) | 675 (26.3) | –4.1 (–5.7 to –2.5) | 0.001 |
| Vasopressor days,  mean (SD) | 3.2 (2.8)  [n = 1209] | 2.8 (2.4)  [n = 1217] | 0.4 (0.2 to 0.6) | 0.002 |
| Ventilator days, mean (SD) | 13.4 (24.1)  [n = 535] | 9.4 (14.4)  [n = 536] | 4.0 (1.6 to 6.4) | <0.001 |
| Length of stay, mean (SD),  days |  |  |  |  |
| ICU | 15.2 (26.7) | 9.4 (14.4) | 5.8 (4.6 to 7.0) | <0.001 |
| Hospital | 47.1 (86.7) | 23.8 (24.6) | 23.3 (19.8 to 26.8) | <0.001 |
| Hospital costs, mean (SD),  U.S. $1000 | 13.2 (18.3) | 11.3 (16.6) | 1.9 (1.0 to 2.8) | <0.001 |
| **Genitourinary (n = 2792/2636)** |  |  |  |  |
| Primary outcome |  |  |  |  |
| Hospital mortality, No. (%) | 378 (13.5) | 421 (16.0) | –2.4 (–3.7 to –1.2) | 0.01 |
| Secondary outcomes |  |  |  |  |
| 90-d mortality, No. (%) | 633 (22.7) | 736 (27.9) | –5.3 (–6.8 to –3.7) | <0.001 |
| Vasopressor days,  mean (SD) | 3.1 (2.9)  [n = 1154] | 2.7 (1.7)  [n = 1167] | 0.4 (0.3 to 0.5) | <0.001 |
| Ventilator days, mean (SD) | 12.2 (23.1)  [n = 404] | 12.8 (29.9)  [n = 381] | –0.5 (–1.3 to 0.2) | 0.78 |
| Length of stay, mean (SD),  days |  |  |  |  |
| ICU | 15.2 (25.5) | 10.2 (17.1) | 5.0 (4.8 to 5.3) | <0.001 |
| Hospital | 44.3 (83.7) | 23.4 (27.7) | 21.0 (20.6 to 21.4) | <0.001 |
| Hospital costs, mean (SD),  U.S. $1000 | 10.1 (12.0) | 9.1 (12.4) | 1.0 (0.4 to 1.6) | 0.003 |
| **Septic shock (n = 9034/9084)** |  |  |  |  |
| Primary outcome |  |  |  |  |
| Hospital mortality, No. (%) | 2436 (27.0) | 2655 (29.2) | –2.3 (–3.2 to –1.3) | 0.001 |
| Secondary outcomes |  |  |  |  |
| 90-d mortality, No. (%) | 2925 (32.4) | 3358 (37.0) | –4.6 (–5.6 to –3.6) | <0.001 |
| Vasopressor days,  mean (SD) | 3.0 (2.6) | 2.8 (2.1) | 0.24 (0.2 to 0.3) | <0.001 |
| Ventilator days, mean (SD) | 12.8 (23.7)  [n = 4242] | 9.0 (15.4)  [n = 4281] | 3.8 (3.6 to 4.0) | <0.001 |
| Length of stay, mean (SD),  days |  |  |  |  |
| ICU | 17.1 (25.4) | 11.1 (17.1) | 5.9 (5.8 to 6.1) | <0.001 |
| Hospital | 38.5 (53.7) | 25.2 (26.9) | 13.4 (13.2 to 13.6) | <0.001 |
| Hospital costs, mean (SD),  U.S. $1000 | 20.4 (27.4) | 16.5 (19.2) | 3.8 (3.1 to 4.5) | <0.001 |
| **Mechanical ventilation**  **(n = 5206/5234)** |  |  |  |  |
| Primary outcome |  |  |  |  |
| Hospital mortality, No. (%) | 1638 (31.5) | 1838 (35.1) | –3.7 (–4.9 to –2.4) | <0.001 |
| Secondary outcomes |  |  |  |  |
| 90-d mortality, No. (%) | 1902 (36.5) | 2216 (42.3) | –5.8 (–7.1 to –4.5) | <0.001 |
| Vasopressor days,  mean (SD) | 3.2 (3.0)  [n = 4238] | 2.9 (2.3)  [n = 4274] | 0.34 (0.3 to 0.4) | <0.001 |
| Ventilator days, mean (SD) | 11.5 (22.1) | 8.3 (14.6) | 3.2 (3.0 to 3.4) | <0.001 |
| Outcomes | Vitamin C | Control | Difference (95% CI) | *P* value |
| Length of stay, mean (SD),  days |  |  |  |  |
| ICU | 22.0 (31.7) | 15.0 (20.7) | 6.9 (6.7 to 7.1) | <0.001 |
| Hospital | 41.5 (51.7) | 29.5 (31.2) | 12.0 (11.7 to 12.2) | <0.001 |
| Hospital costs, mean (SD),  U.S. $1000 | 27.9 (33.5) | 22.3 (22.7) | 5.6 (4.5 to 6.7) | <0.001 |
| **Renal replacement therapy**  **(n = 1047/1244)** |  |  |  |  |
| Primary outcome |  |  |  |  |
| Hospital mortality, No. (%) | 558 (53.3) | 723 (58.1) | –4.8 (–7.9 to –1.8) | 0.02 |
| Secondary outcomes |  |  |  |  |
| 90-d mortality, No. (%) | 568 (54.3) | 783 (62.9) | –8.7 (–11.8 to –5.6) | <0.001 |
| Vasopressor days,  mean (SD) | 3.6 (3.4)  [n = 951] | 3.1 (2.6)  [n = 1094] | 0.5 (0.4 to 0.7) | <0.001 |
| Ventilator days, mean (SD) | 17.8 (27.1)  [n = 781] | 11.0 (14.7)  [n = 836] | 6.7 (6.3 to 7.2) | <0.001 |
| Length of stay, mean (SD),  days |  |  |  |  |
| ICU | 23.1 (29.5) | 14.2 (19.6) | 8.9 (8.5 to 9.3) | <0.001 |
| Hospital | 45.4 (52.6) | 29.1 (32.9) | 16.2 (15.7 to 16.8) | <0.001 |
| Hospital costs, mean (SD),  U.S. $1000 | 45.5 (53.4) | 29.2 (34.5) | 16.2 (12.6 to 19.8) | <0.001 |

*CCI* Charlson Comorbidity Index, *ICU* intensive care unit

^a^ The mean of all paired differences in the treatment group minus the control group
