# Supplementary material for: Vitamin C for ≥ 5 days is associated with decreased hospital mortality in sepsis subgroups: a nationwide cohort study
Source: Crit Care. 2022 Jan 5;26:3. doi: 10.1186/s13054-021-03872-3 (PMC8728994; doi:10.1186/s13054-021-03872-3)
Supplement: Supplementary file 3 — Additional file 3: Fig. S1. Survival from hospital admission to day 90 by subpopulations. Fig. S2. Survival from hospital admission to day 90 by sepsis subpopulations among patients who received vitamin C for ≥5 days and matched controls. Fig. S3. Association between vitamin C monotherapy and hospital mortality compared with in combination with corticosteroids and/or thiamine in the sepsis subpopulations. [file 13054_2021_3872_MOESM3_ESM.docx]

**Fig. S1** Survival from hospital admission to day 90 by subpopulations

**a** Age ≥70 years


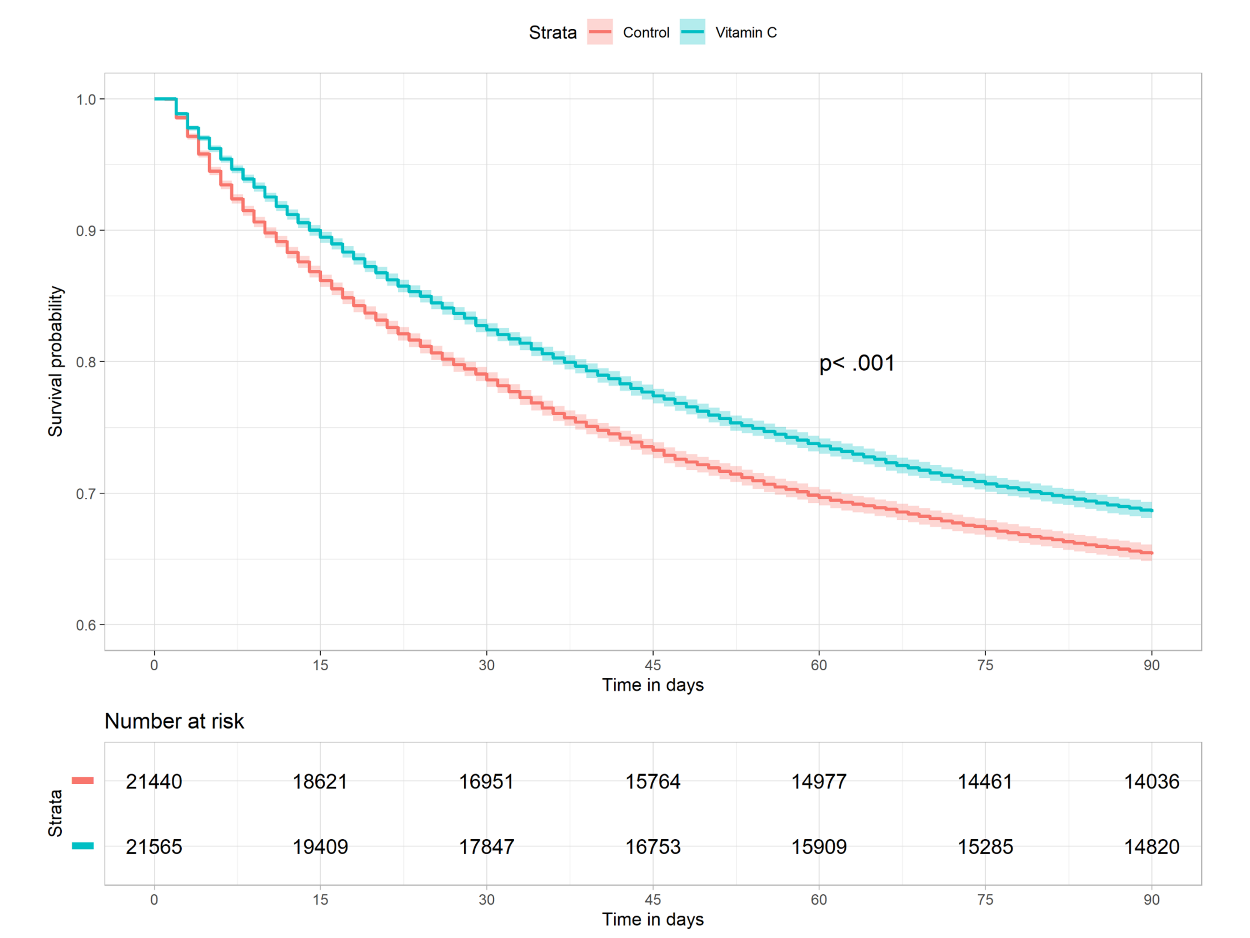


**b** Age <70 years


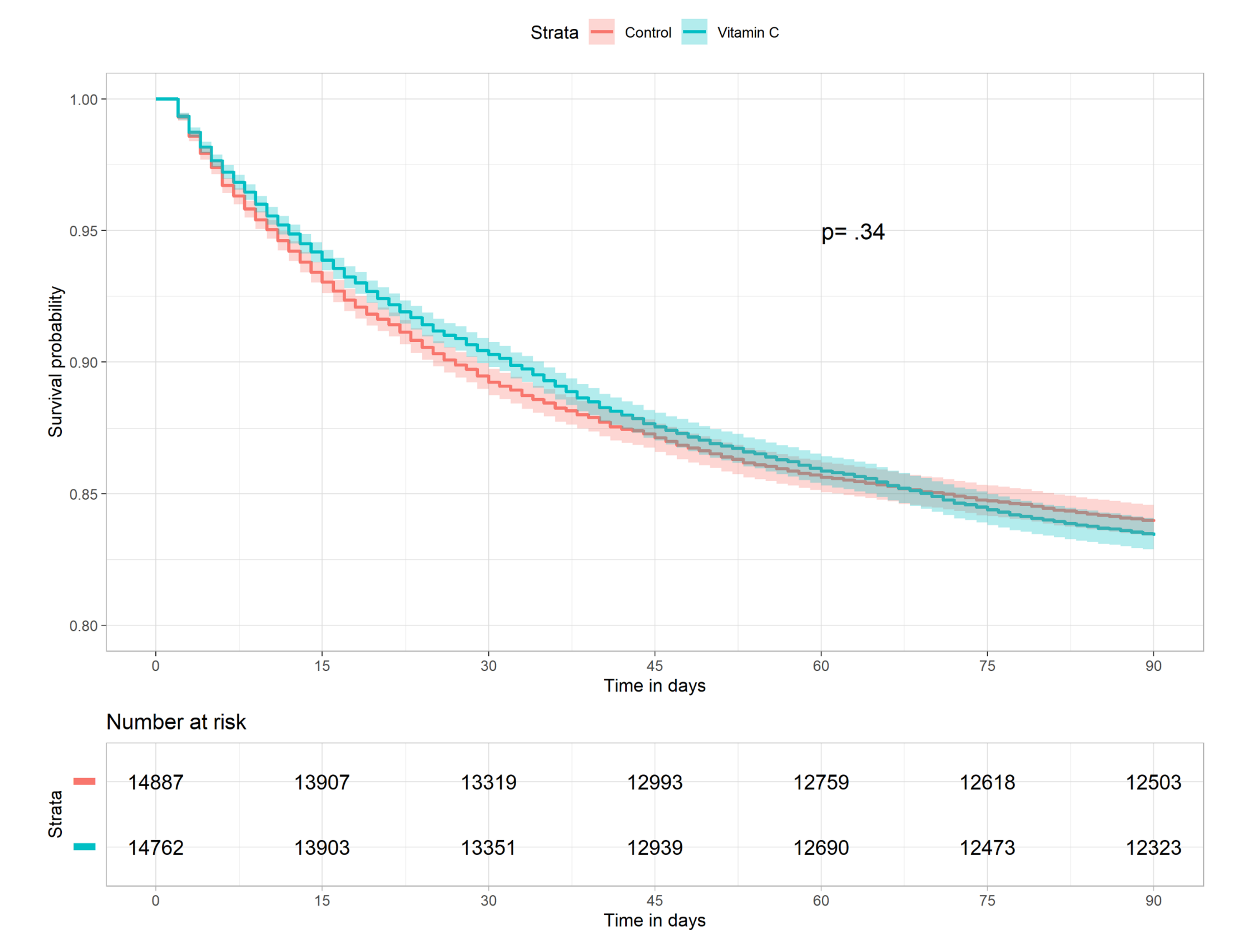


**c** Male


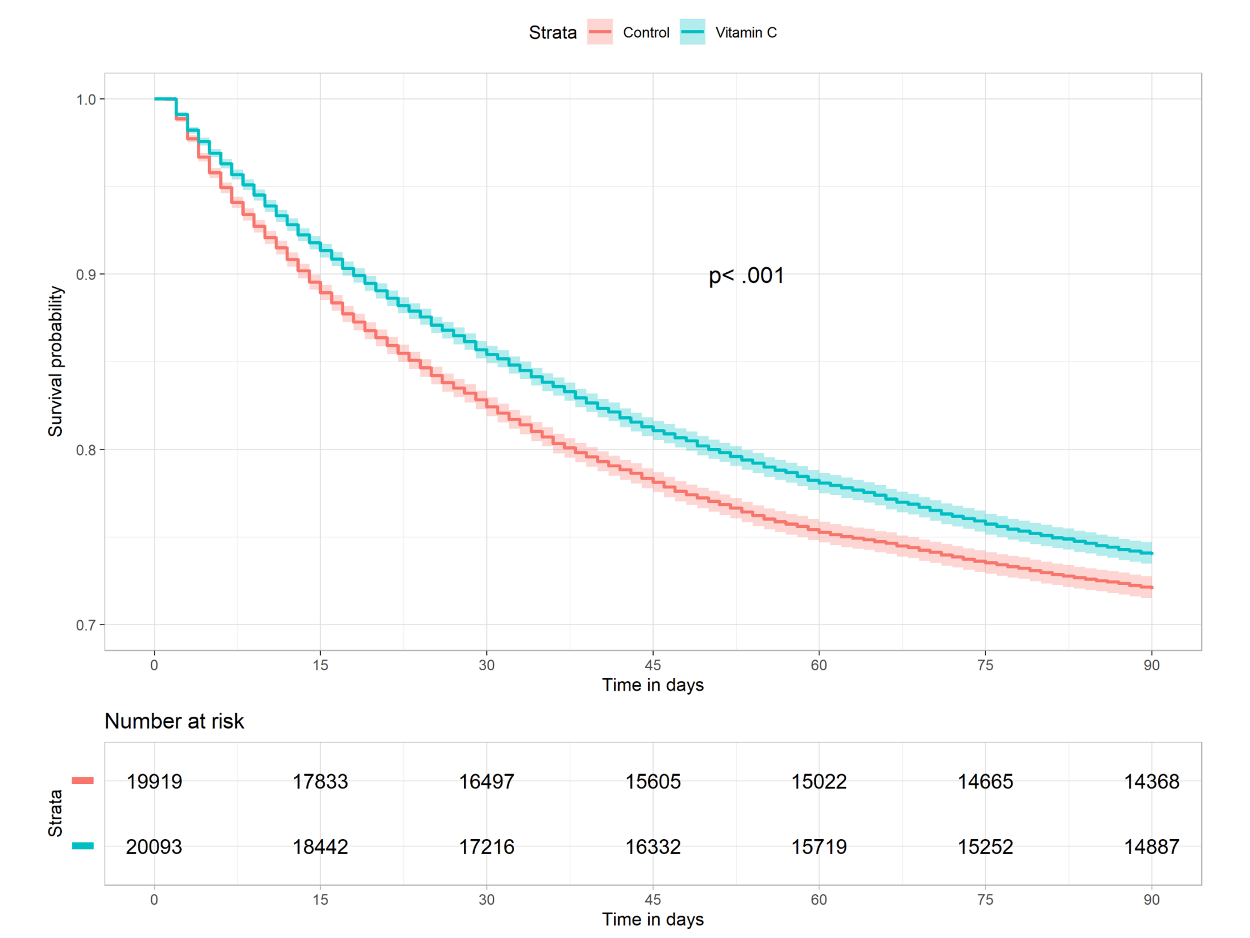


**d** Female


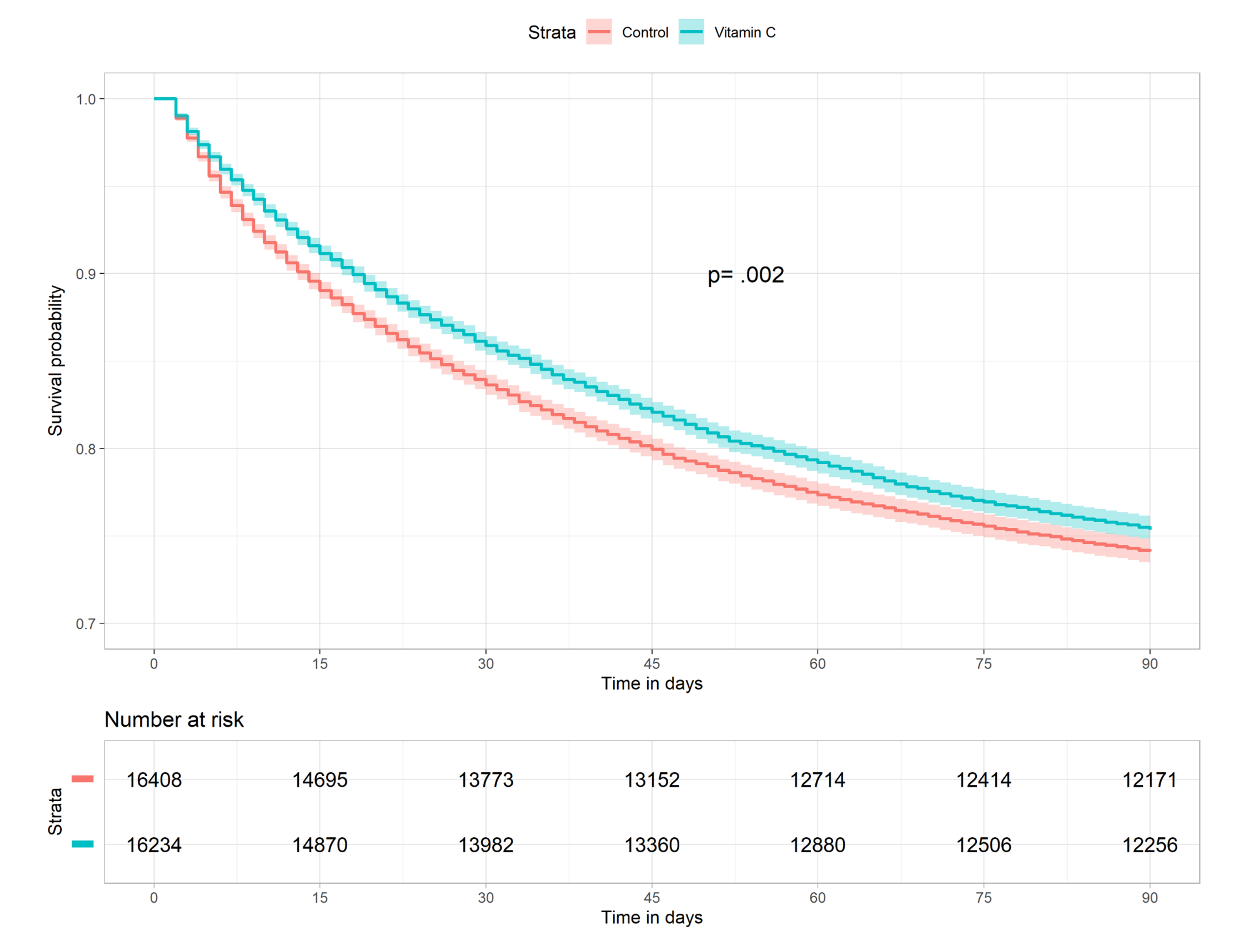


**e** Charlson Comorbidity Index ≥3


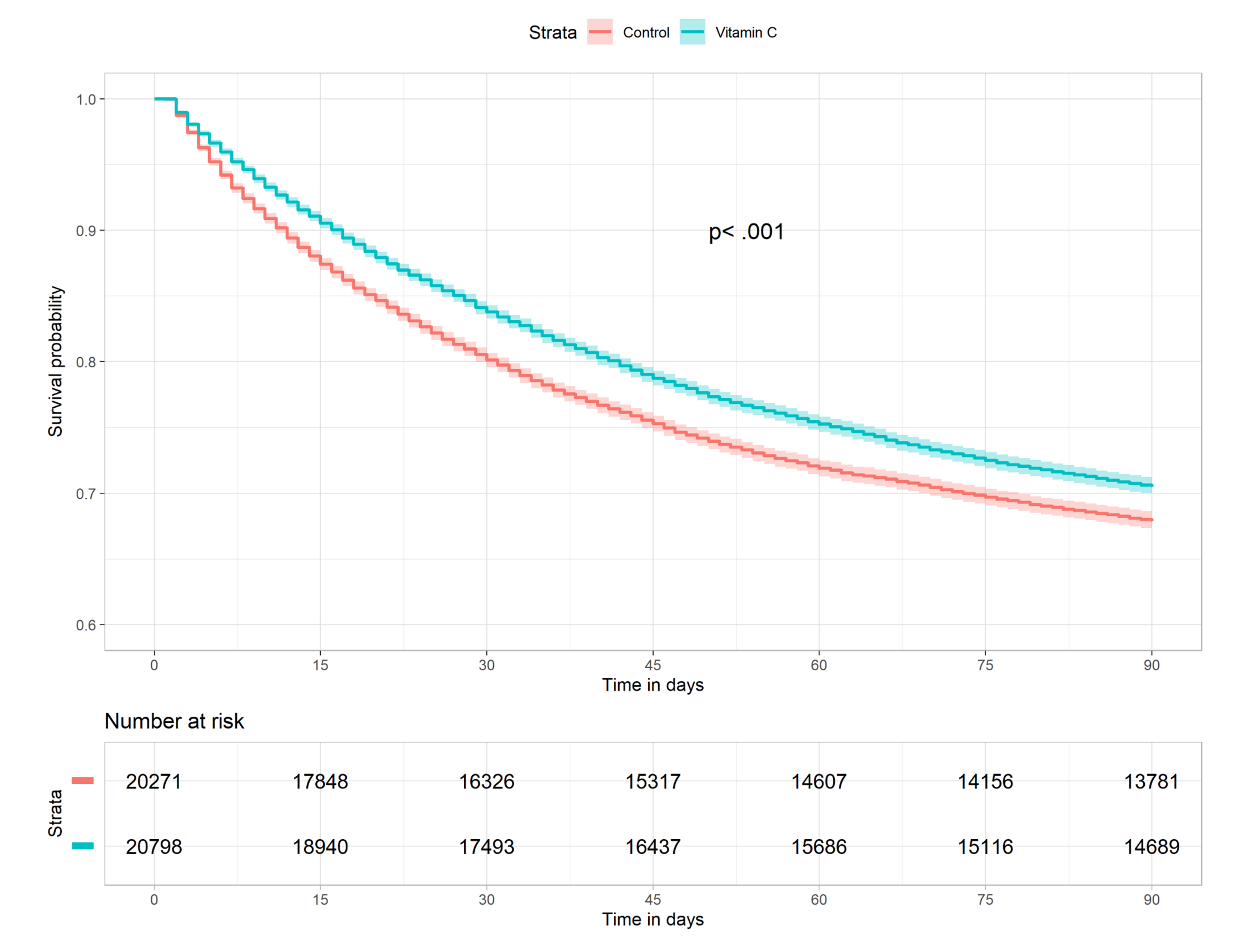


**f** Charlson Comorbidity Index <3


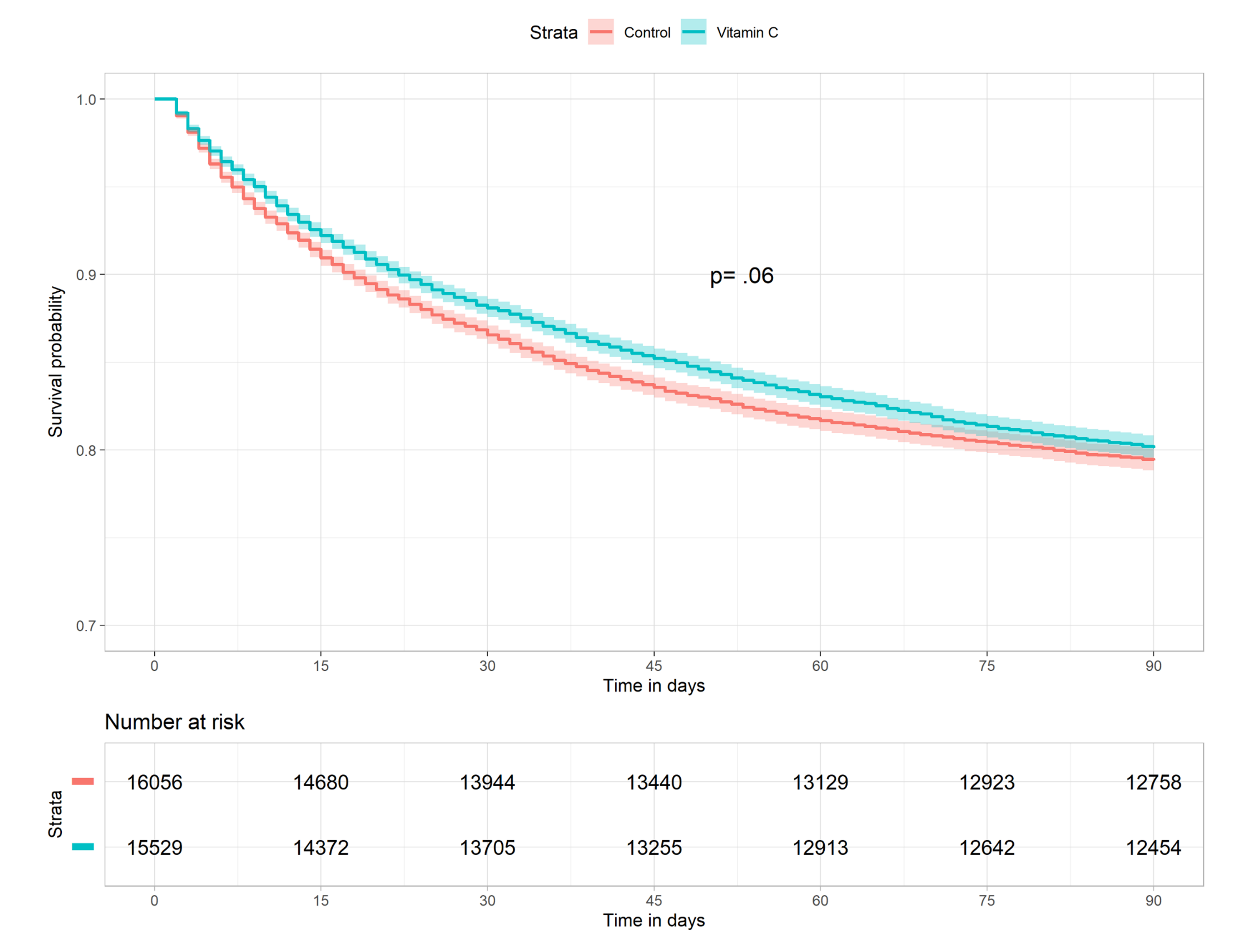


**g** Pneumonia


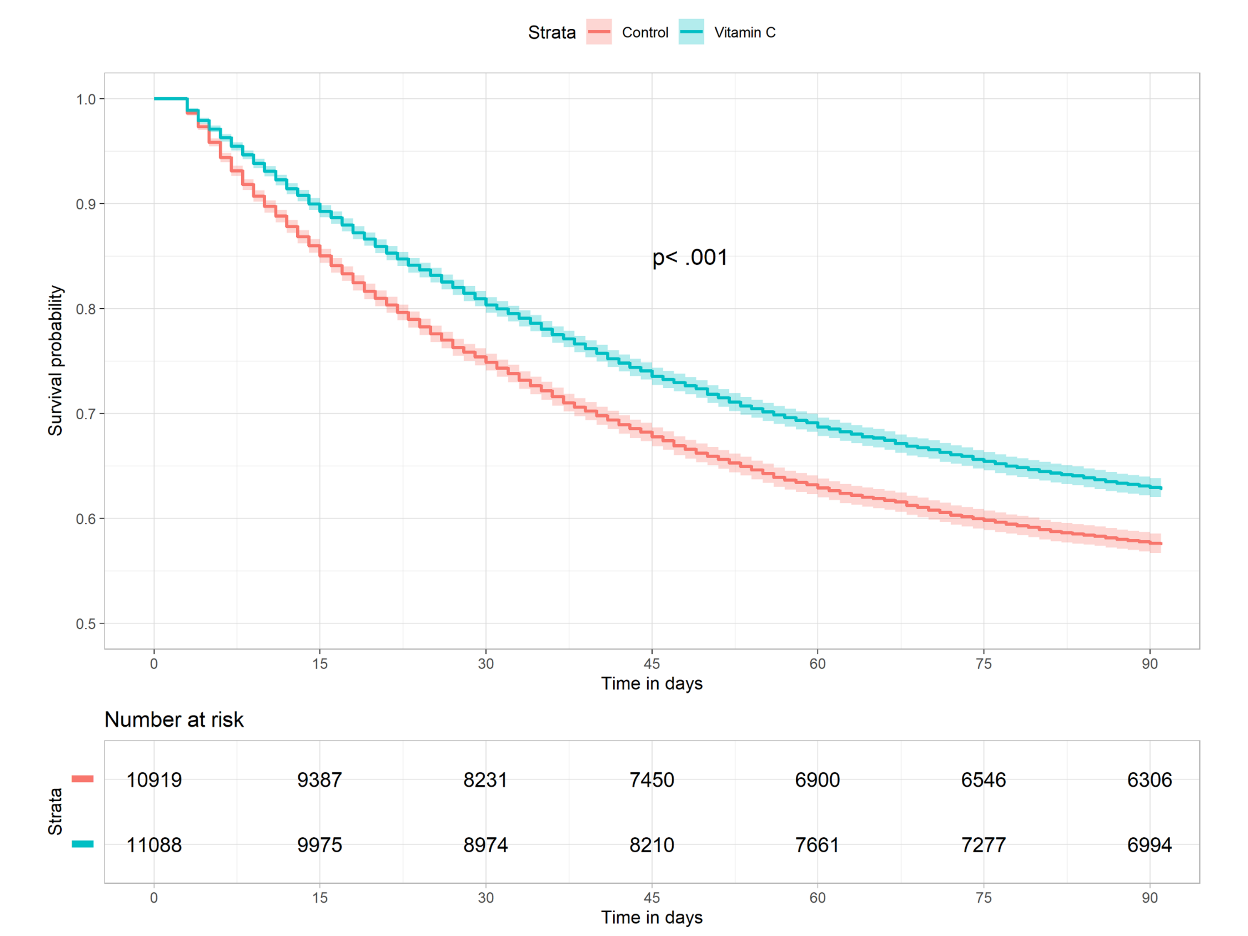


**h** Gastrointestinal


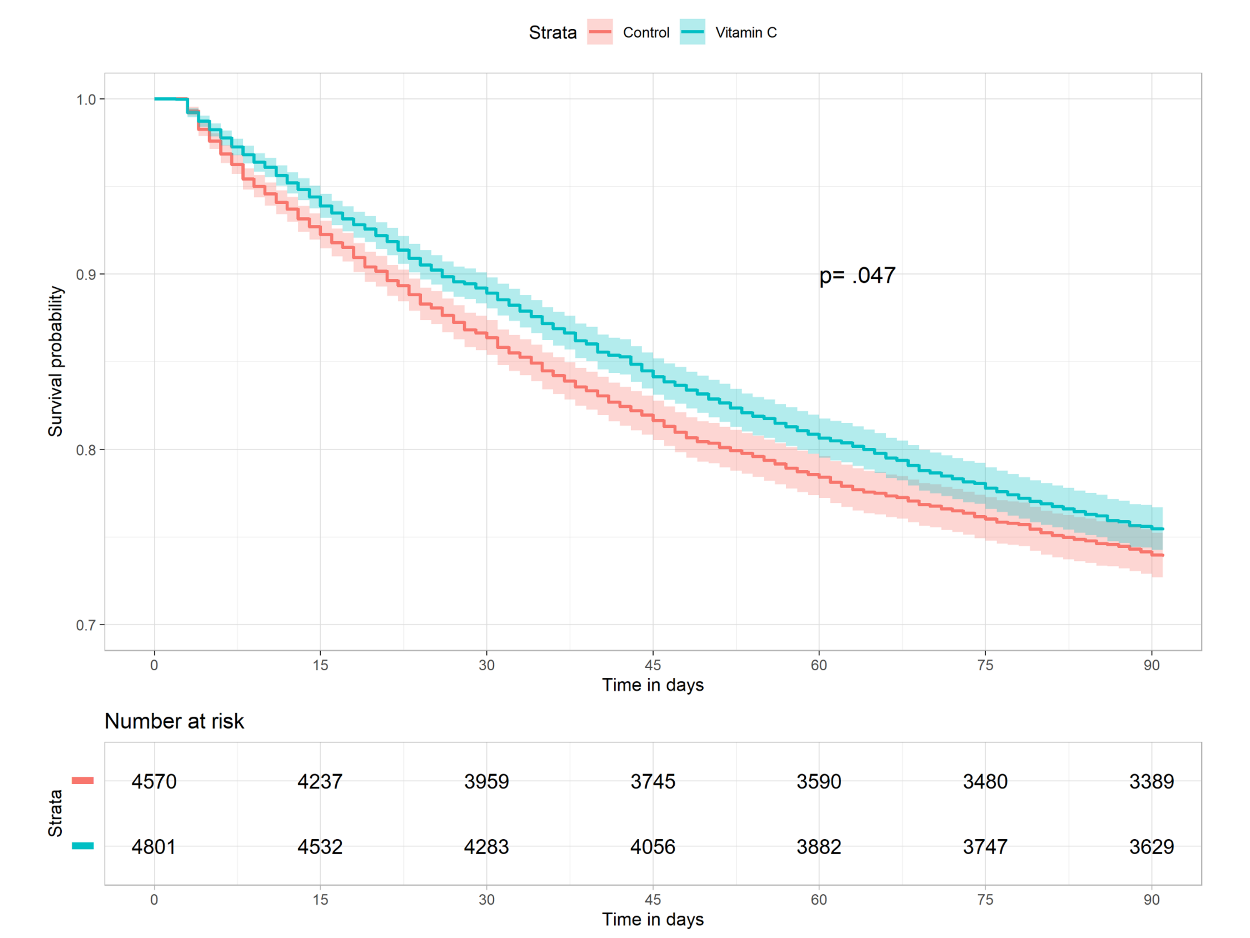


**i** Genitourinary


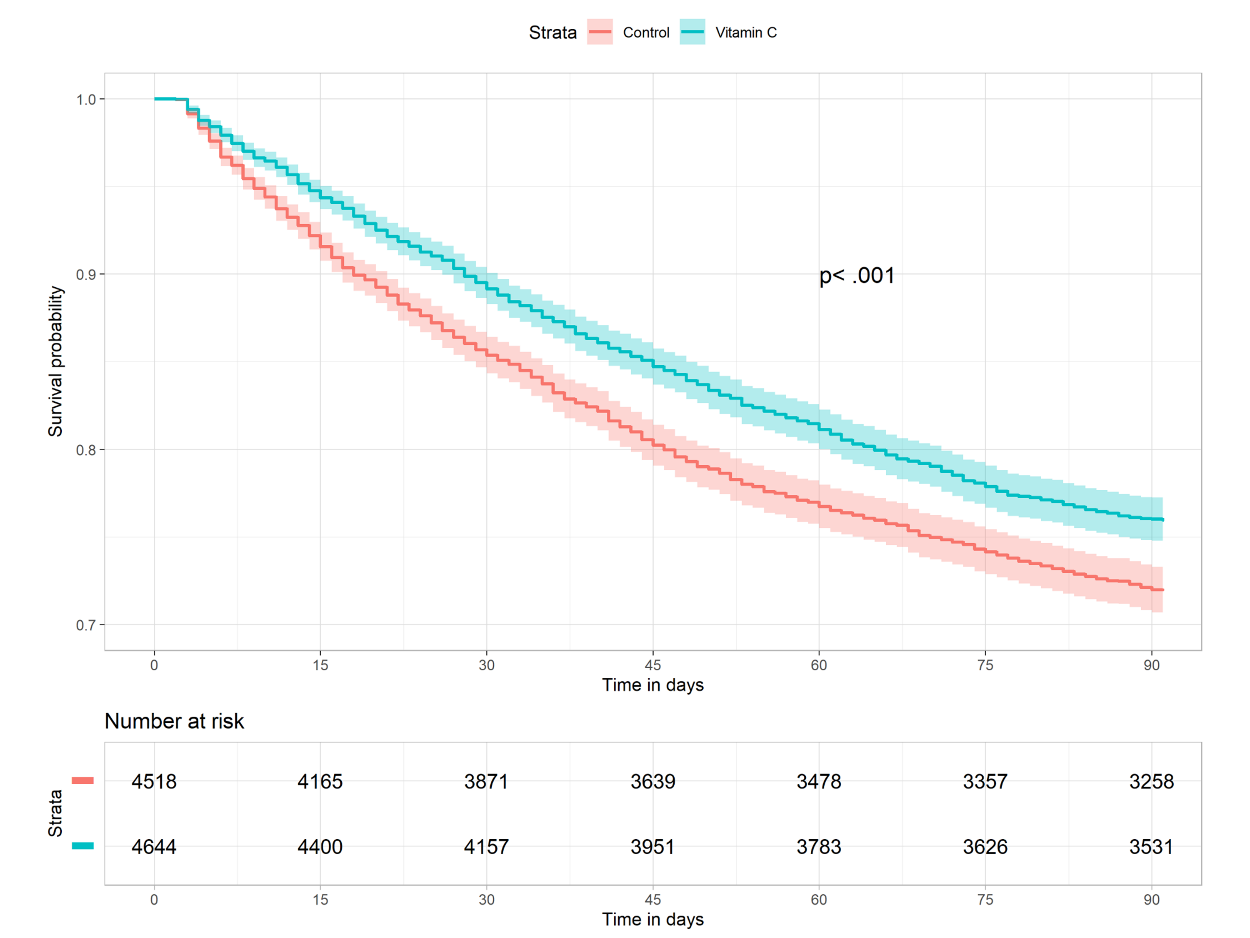


**j** Septic shock


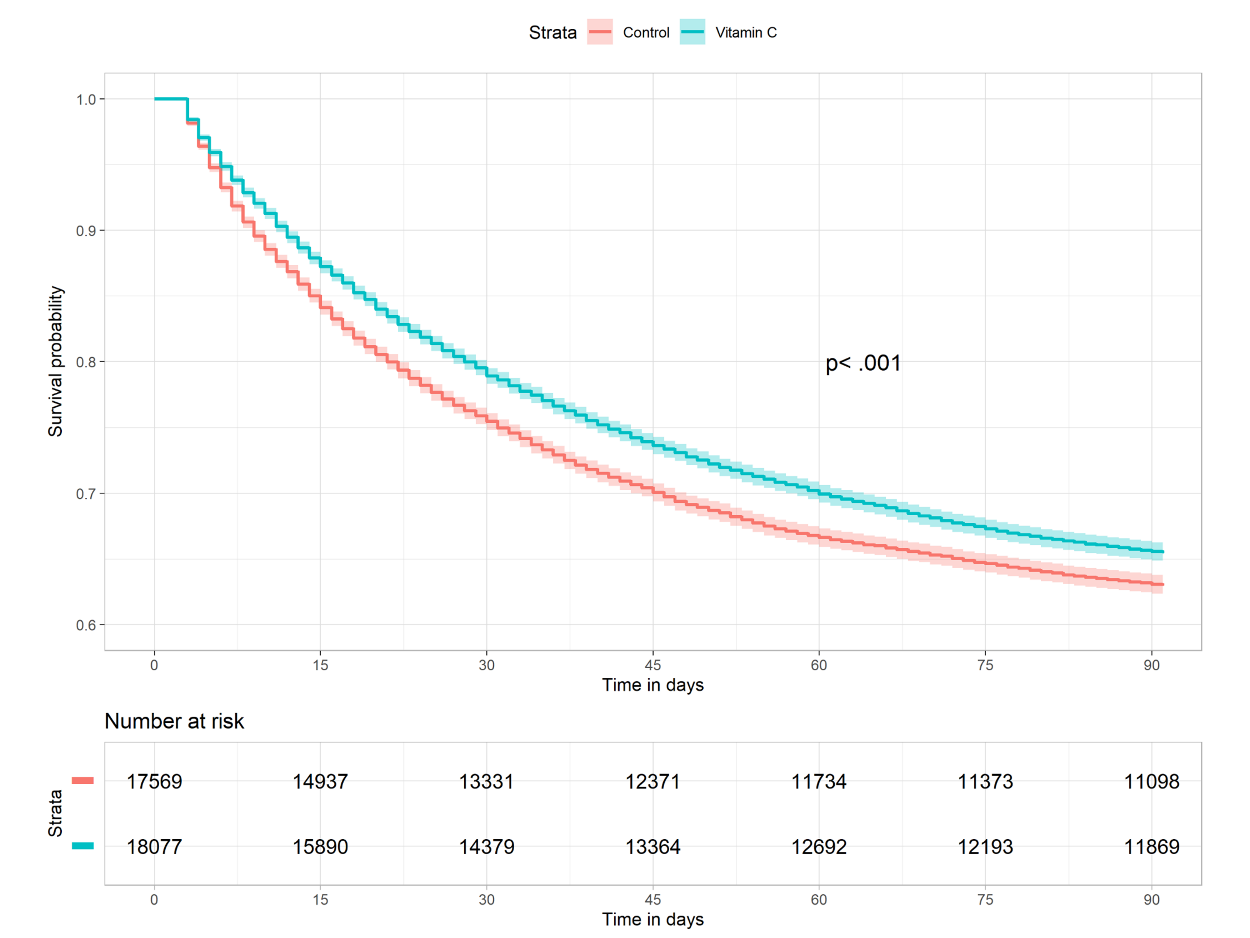


**k** Mechanical ventilation


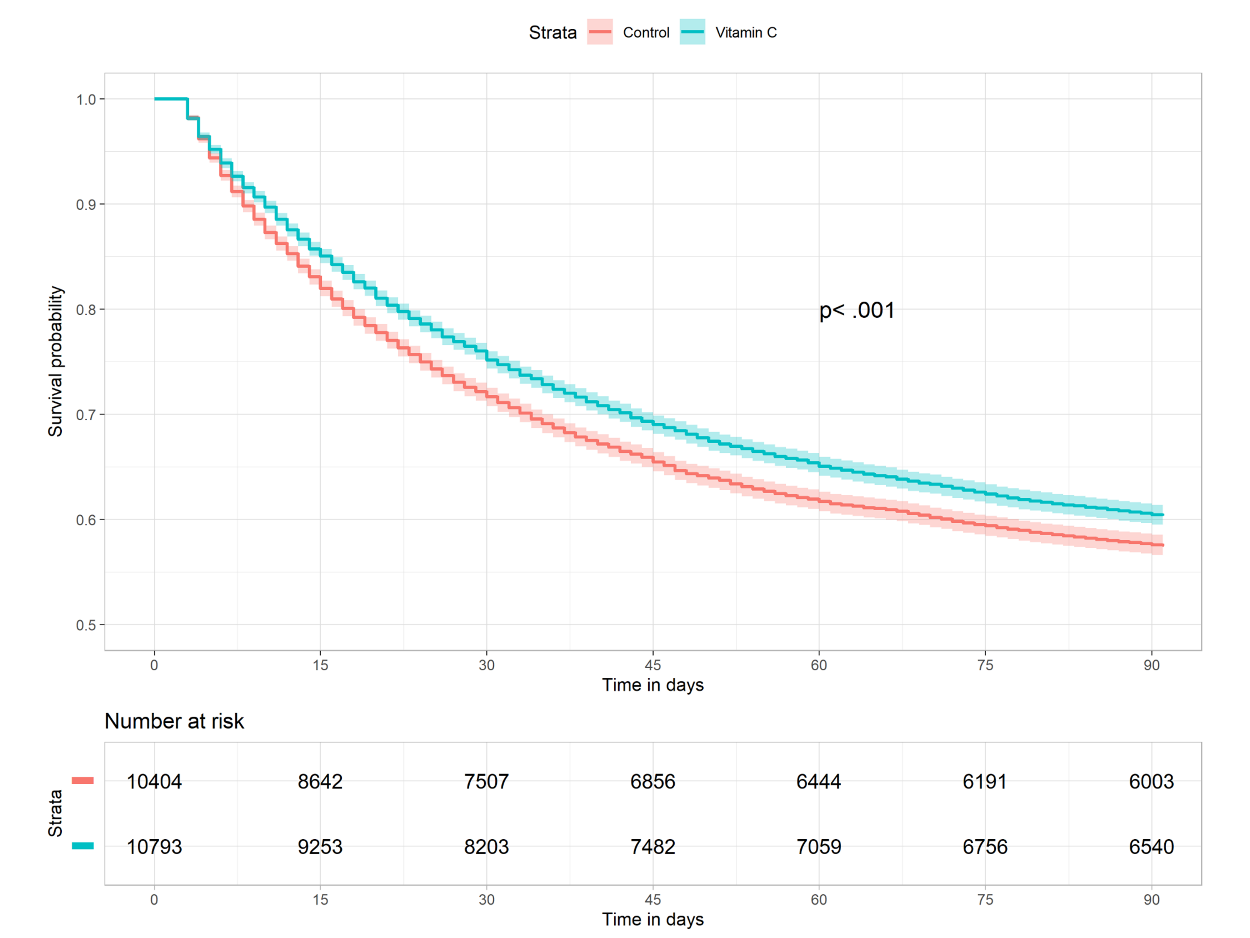


**l** Renal replacement therapy


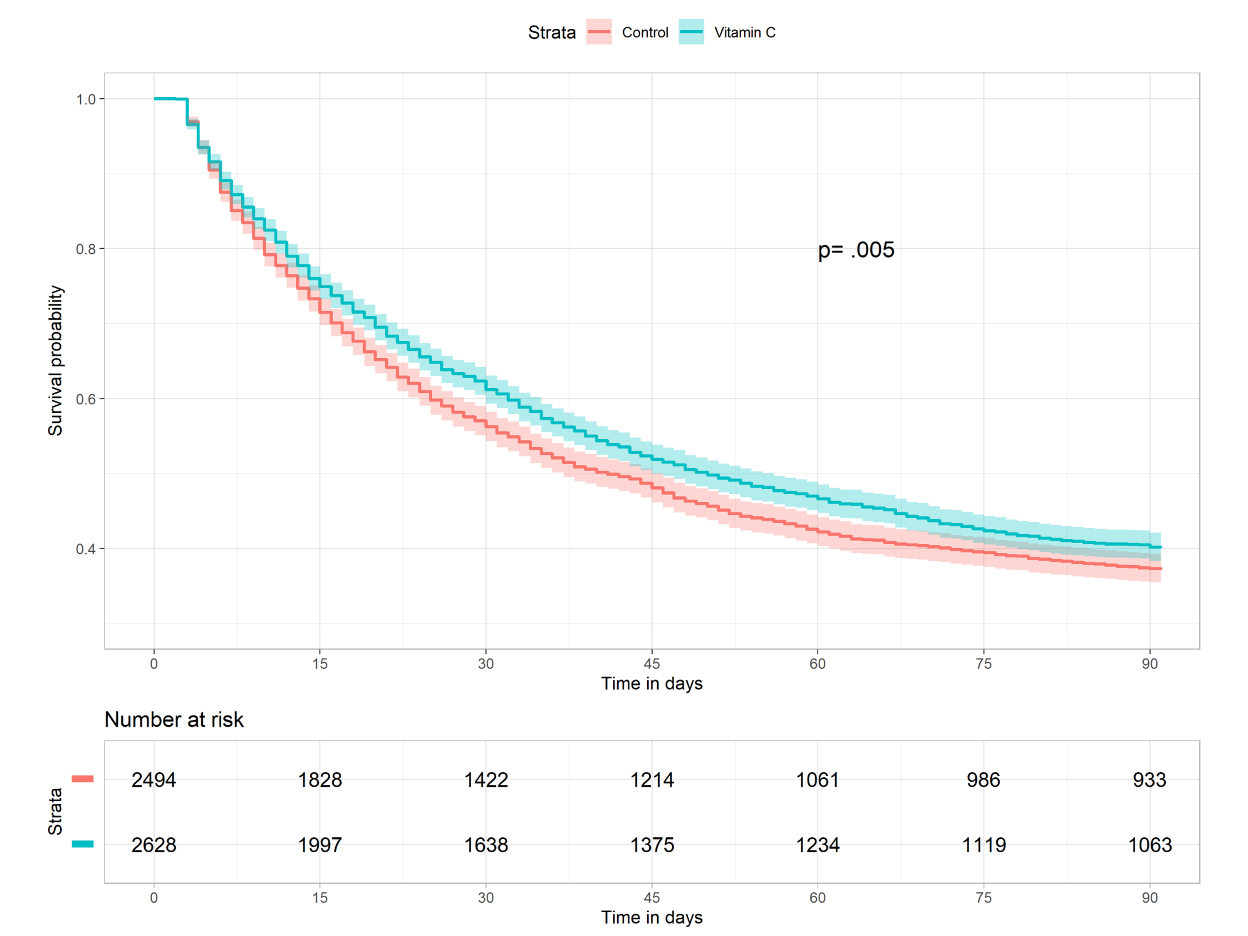


**Fig. S2** Survival from hospital admission to day 90 by sepsis subpopulations among patients who received vitamin C for ≥5 days and matched controls

**a** Age ≥70 years


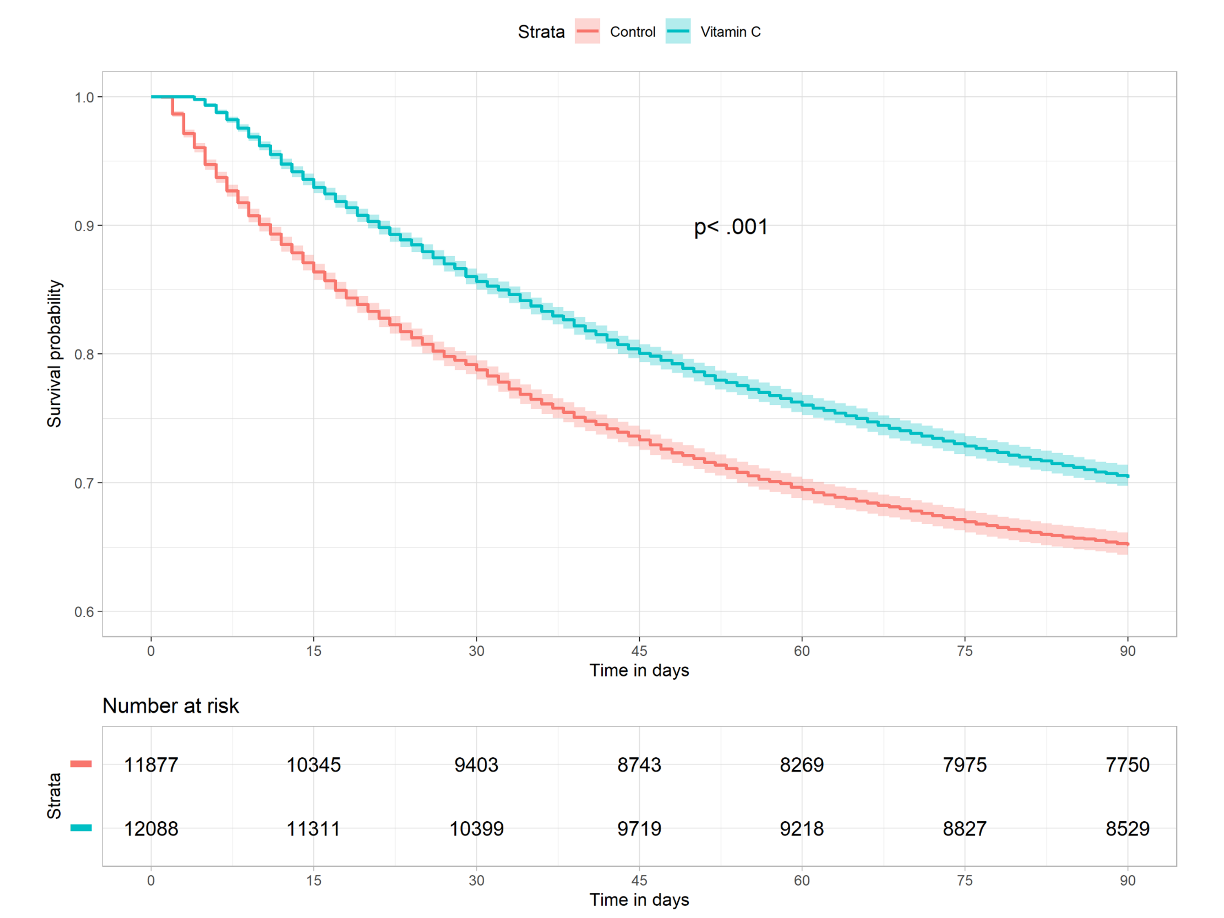


**b** Age <70 years


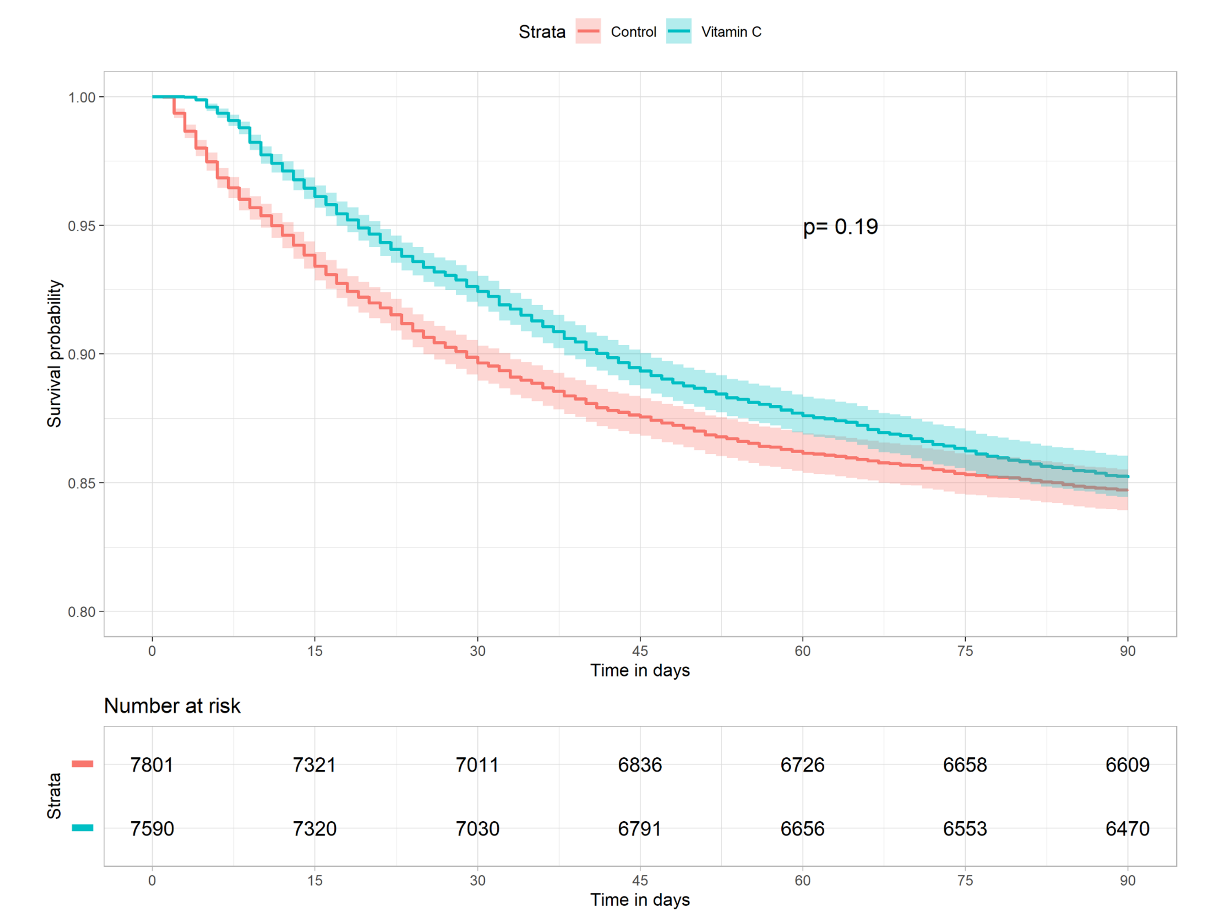


**c** Male


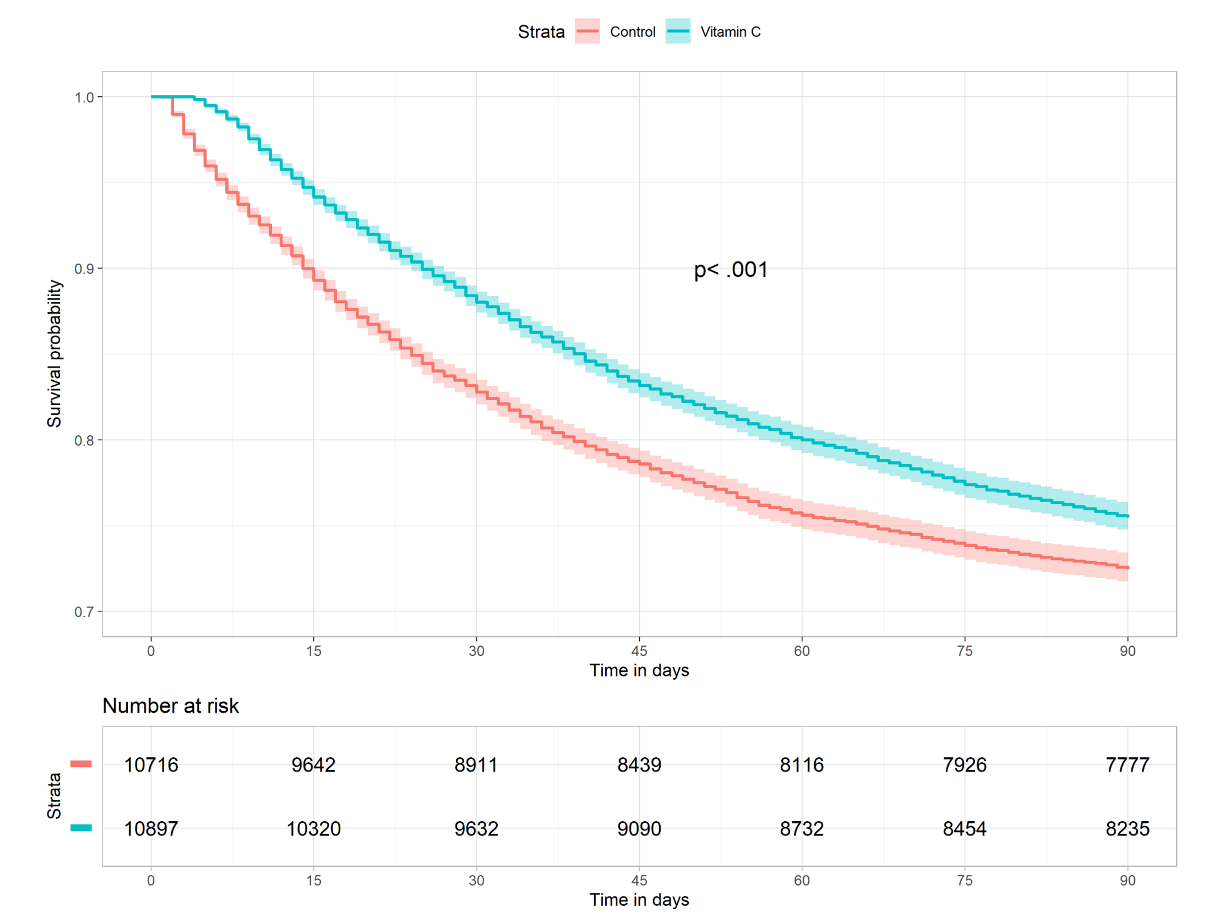


**d** Female


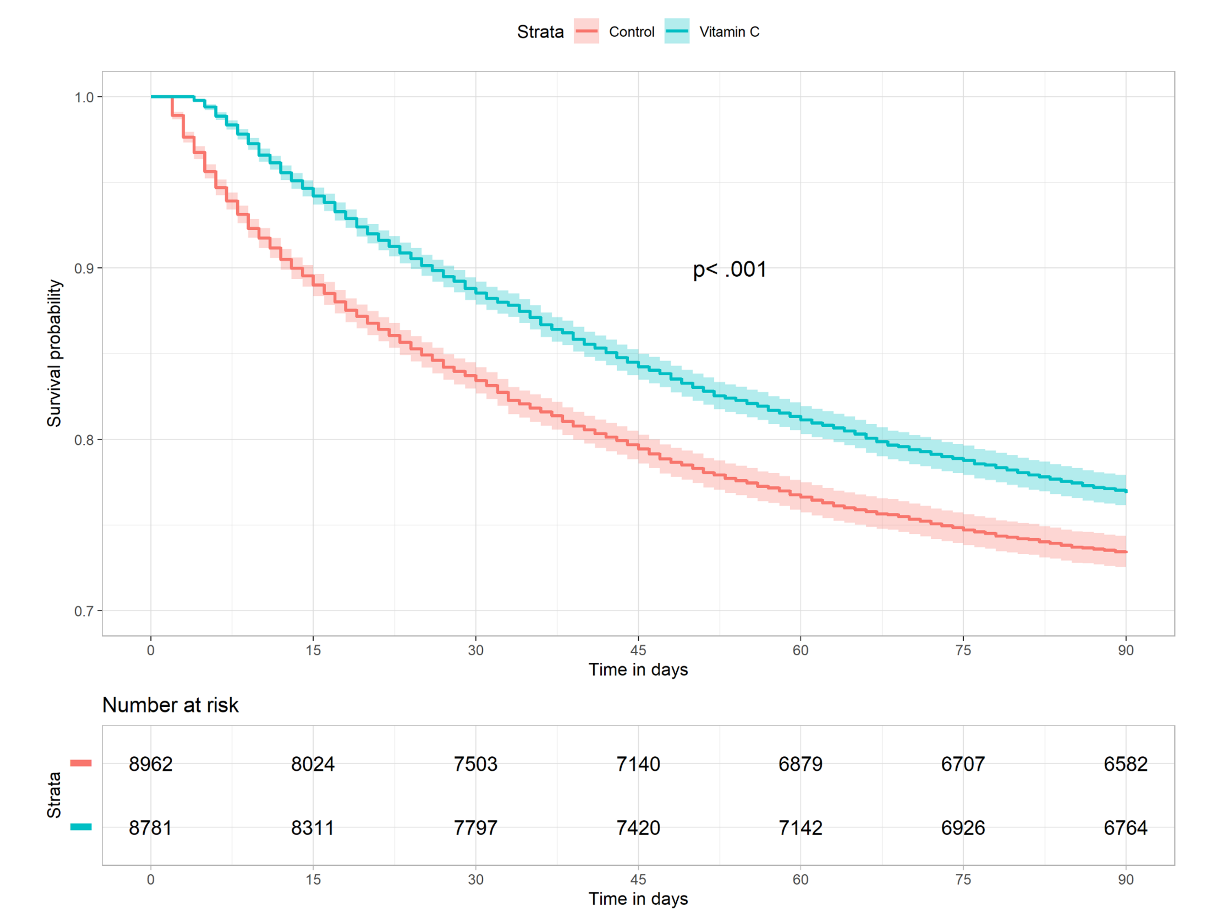


**e** Charlson Comorbidity Index ≥3


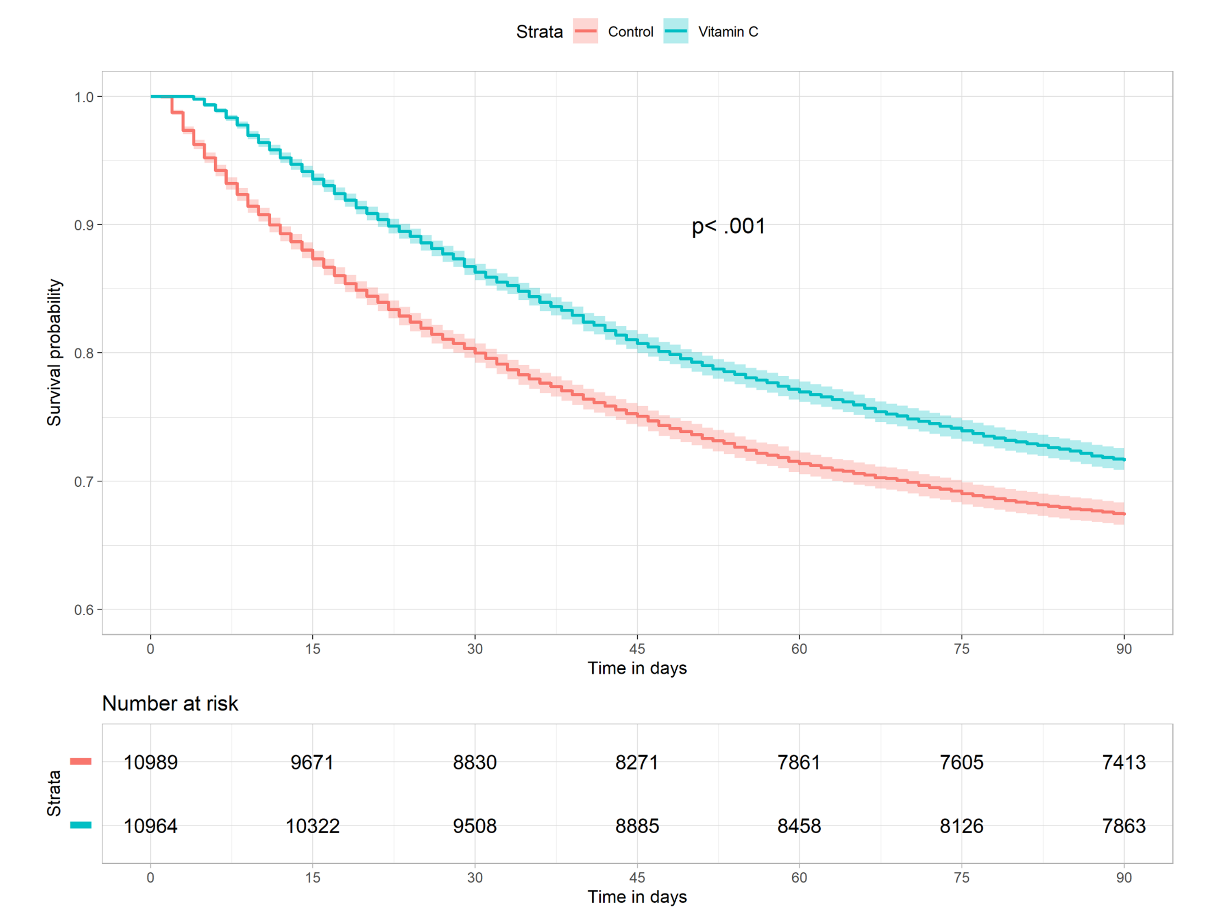


**f** Charlson Comorbidity Index <3


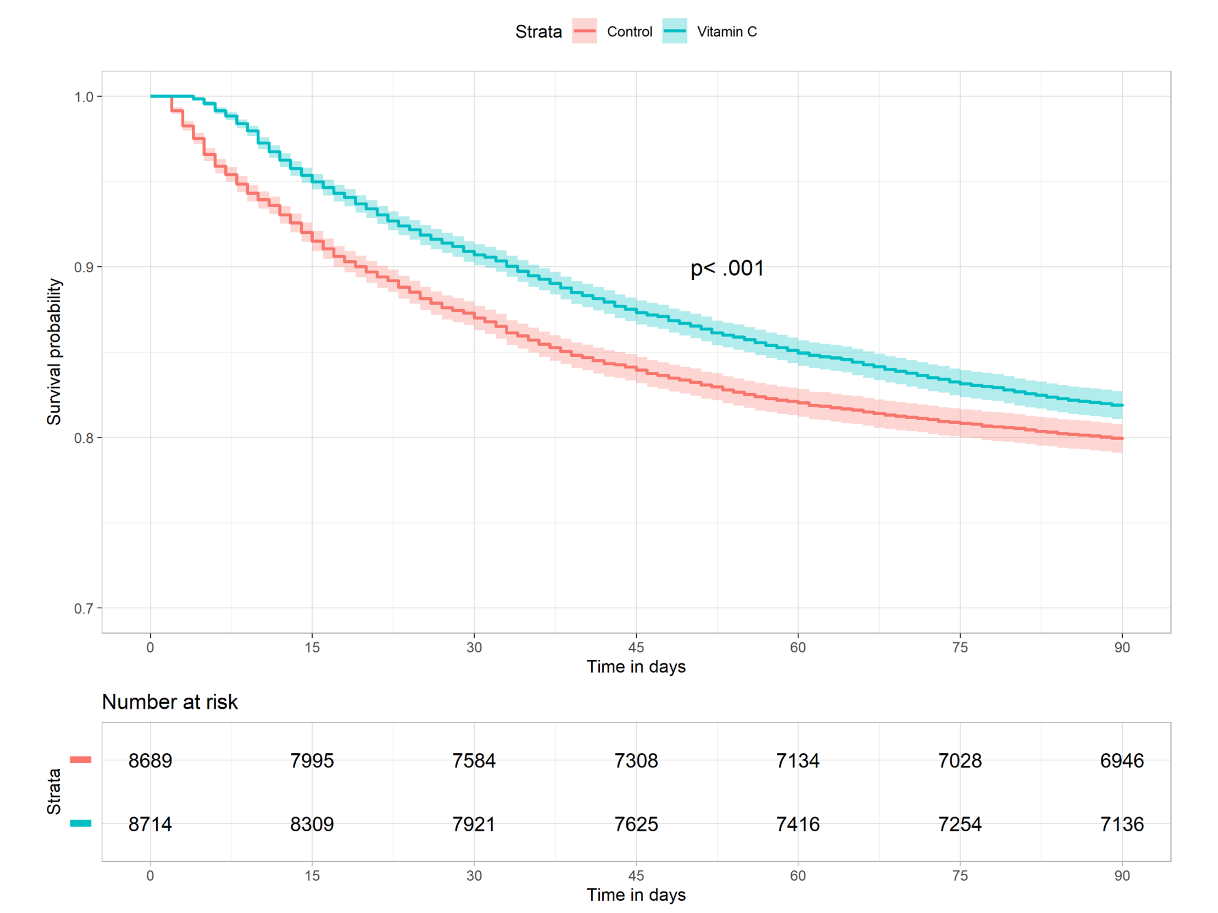


**g** Pneumonia


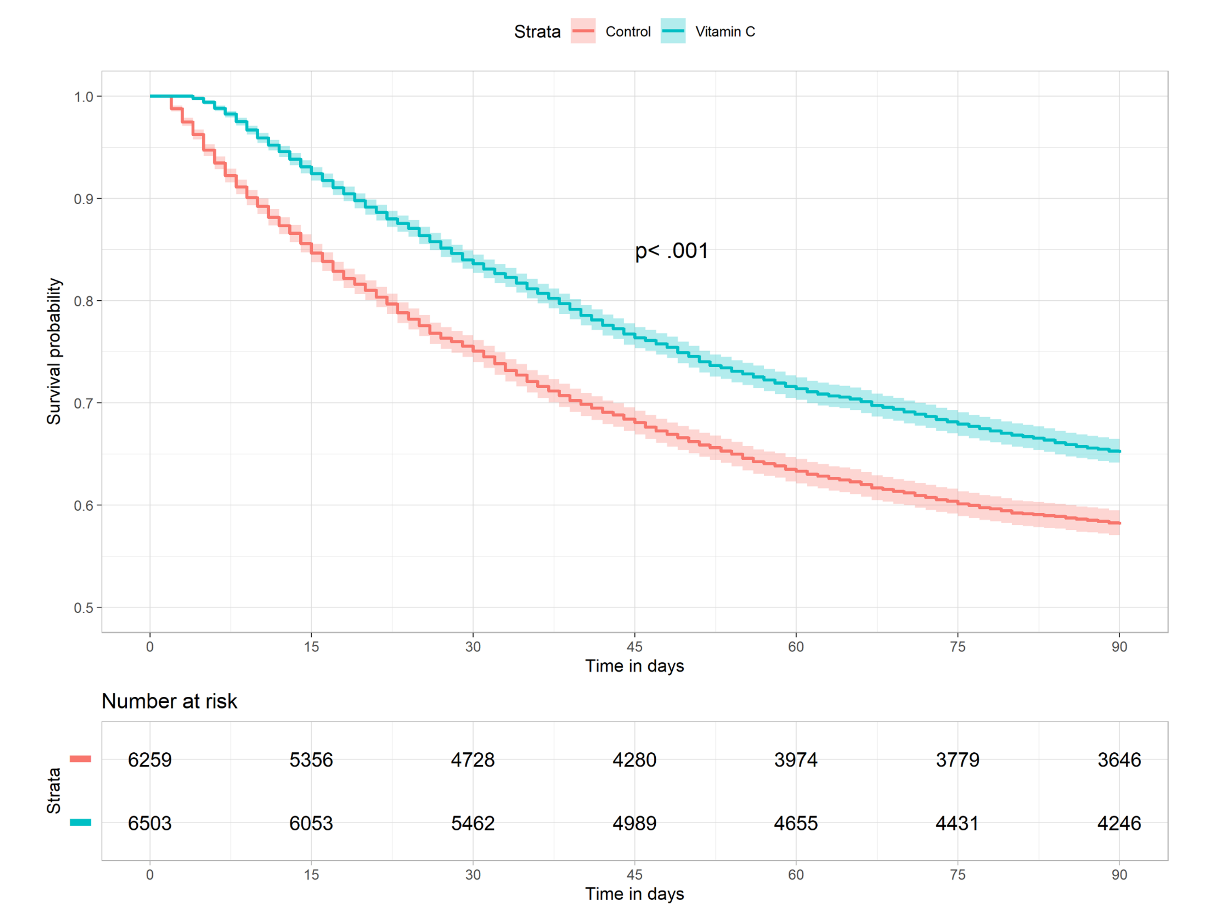


**h** Gastrointestinal


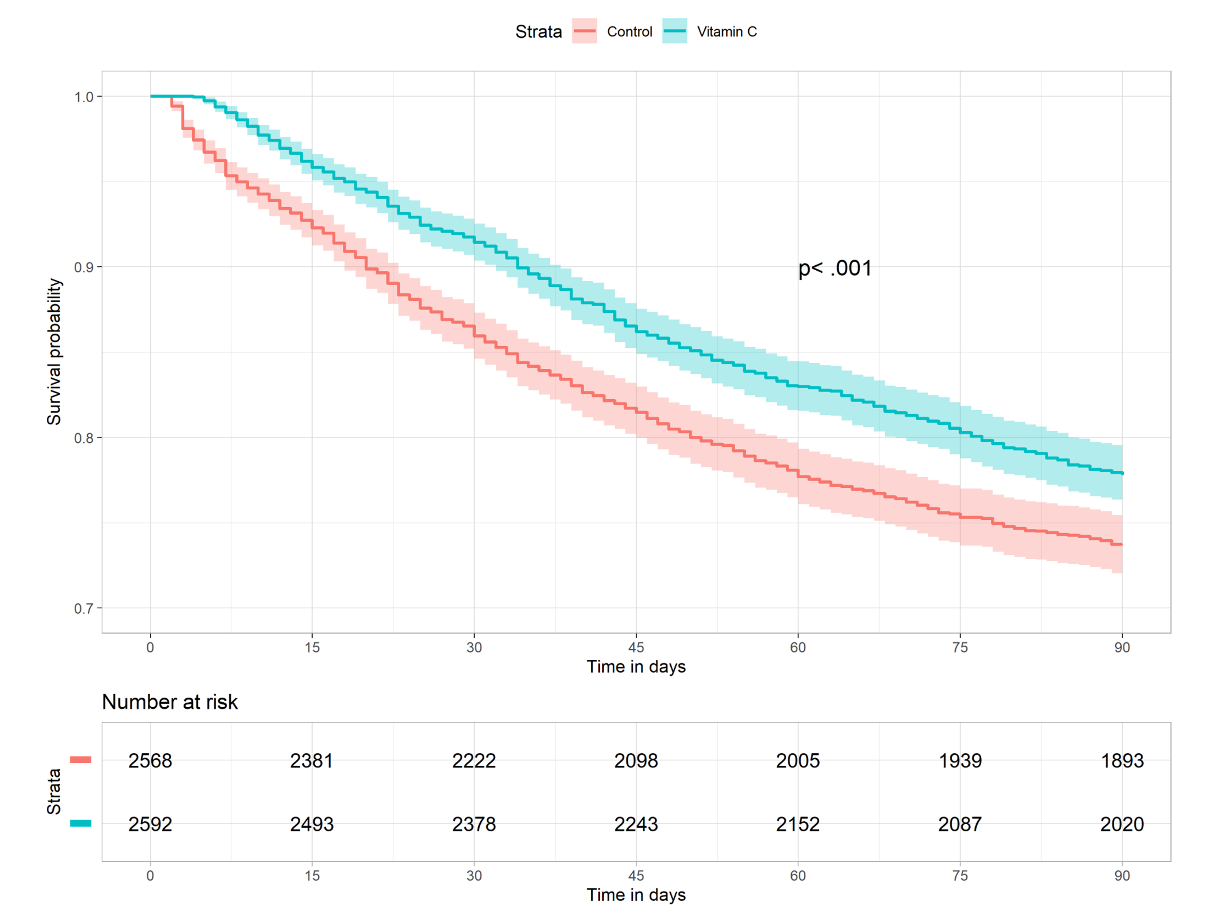


**i** Genitourinary


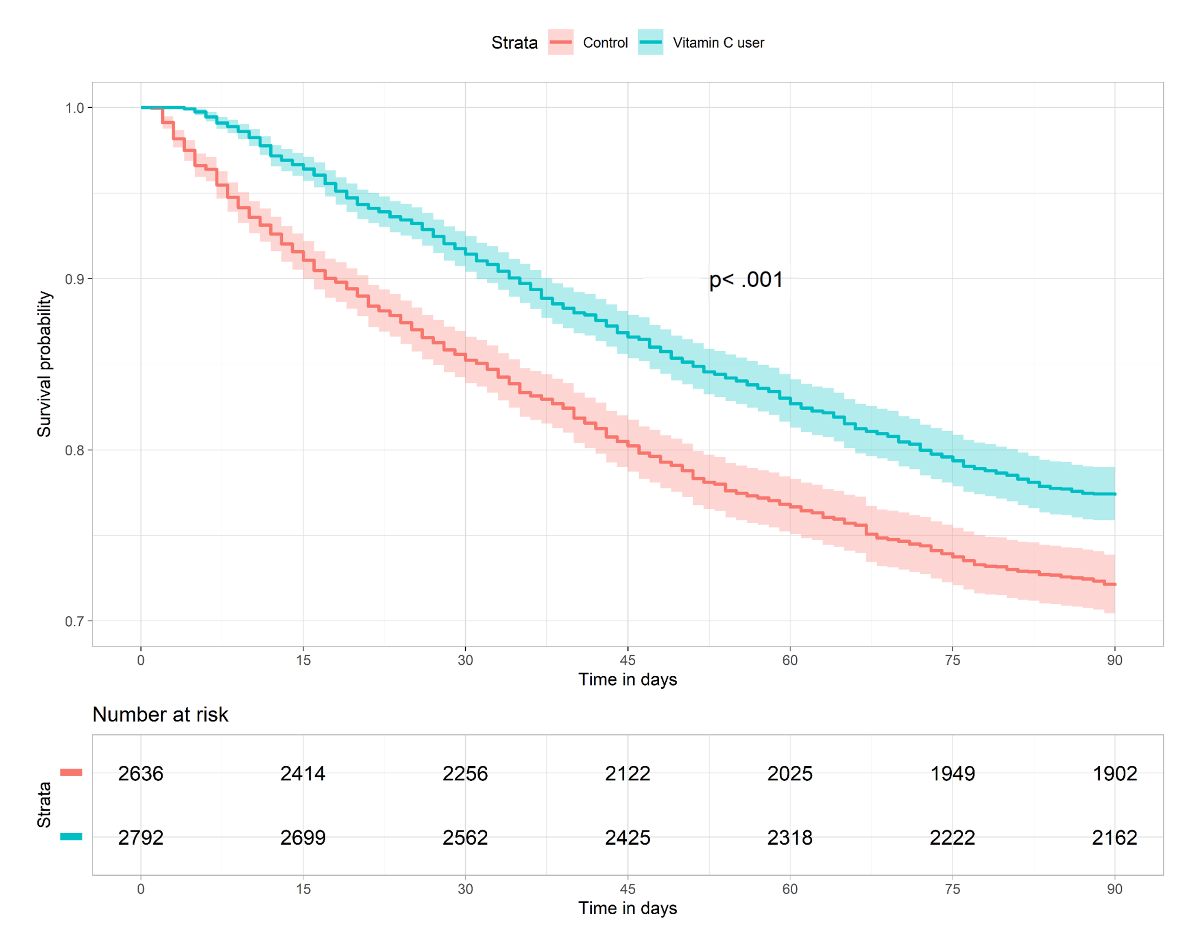


**j** Septic shock


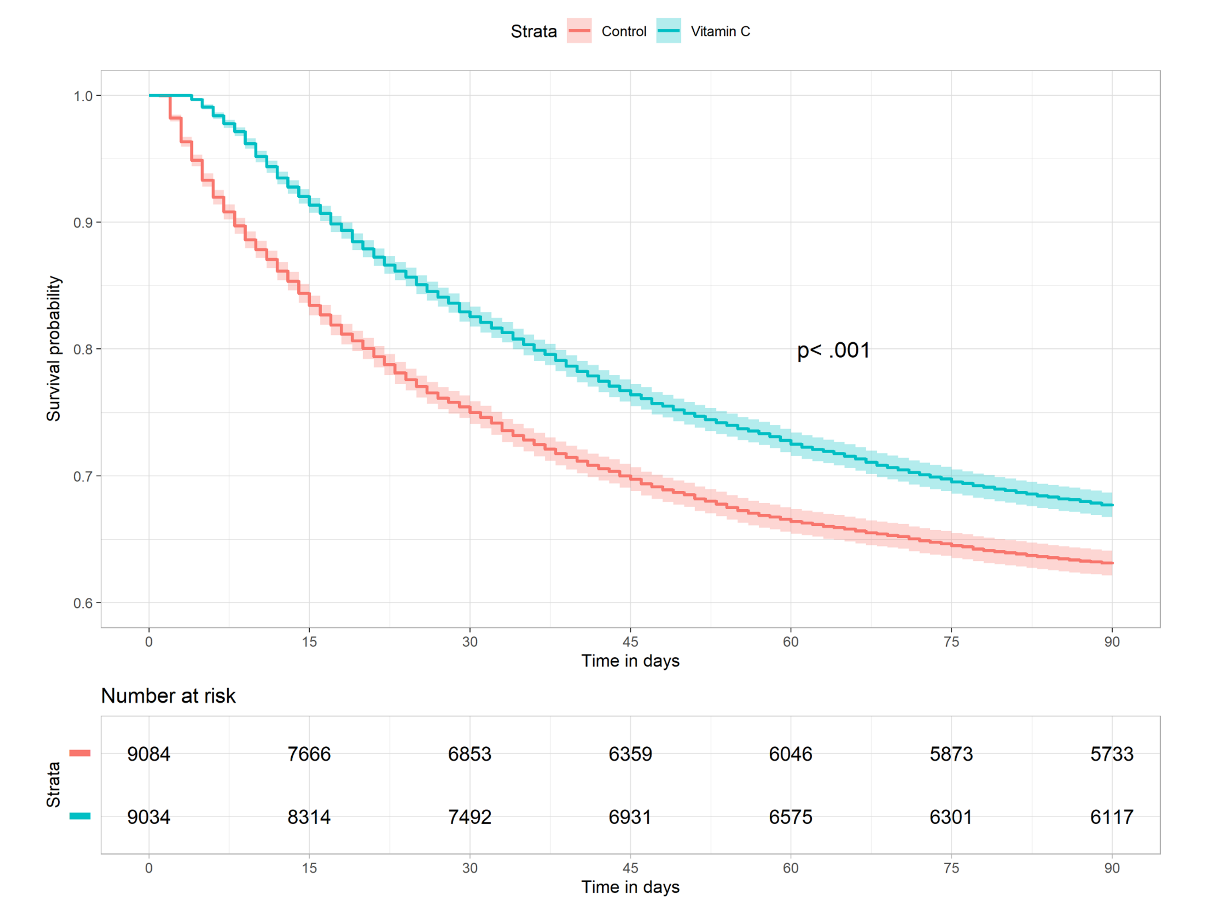


**k** Mechanical ventilation


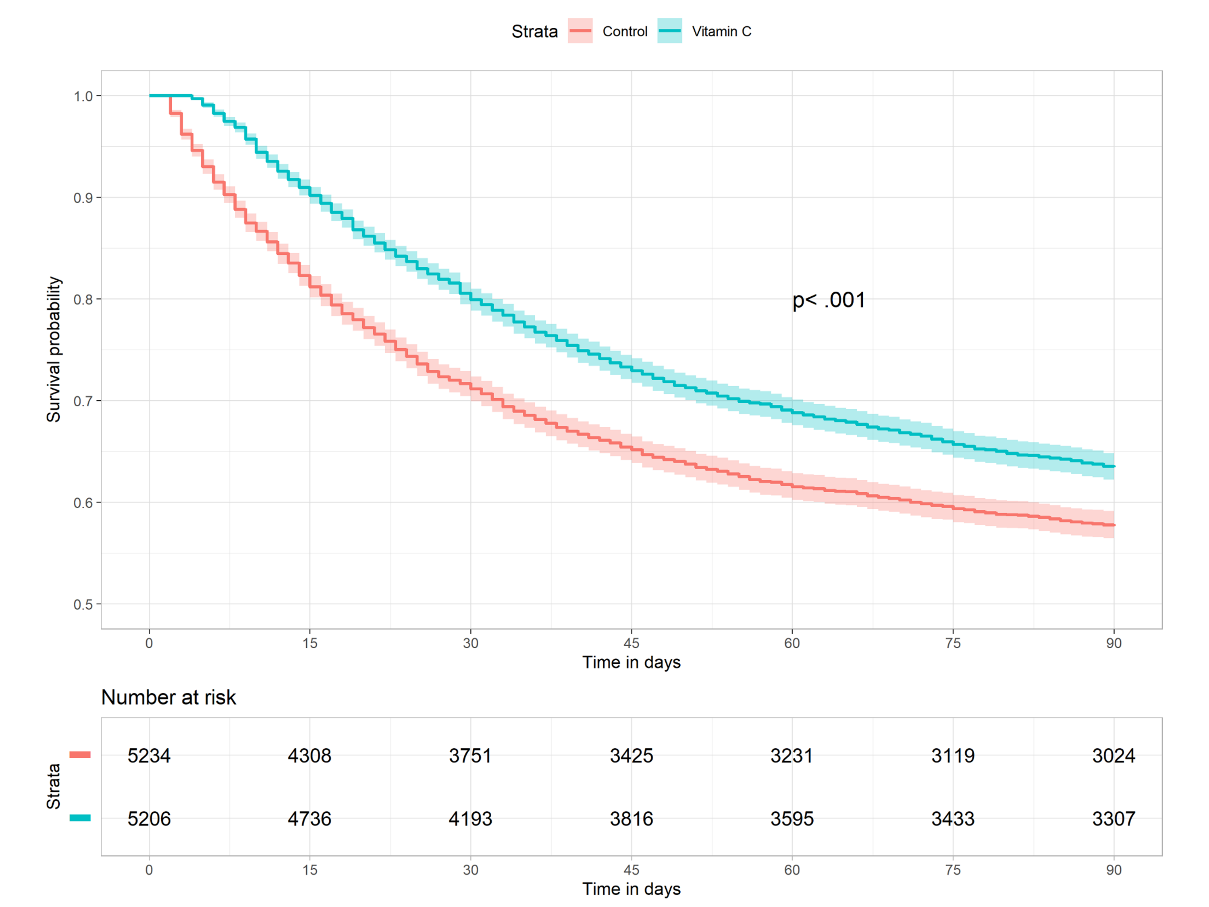


**l** Renal replacement therapy


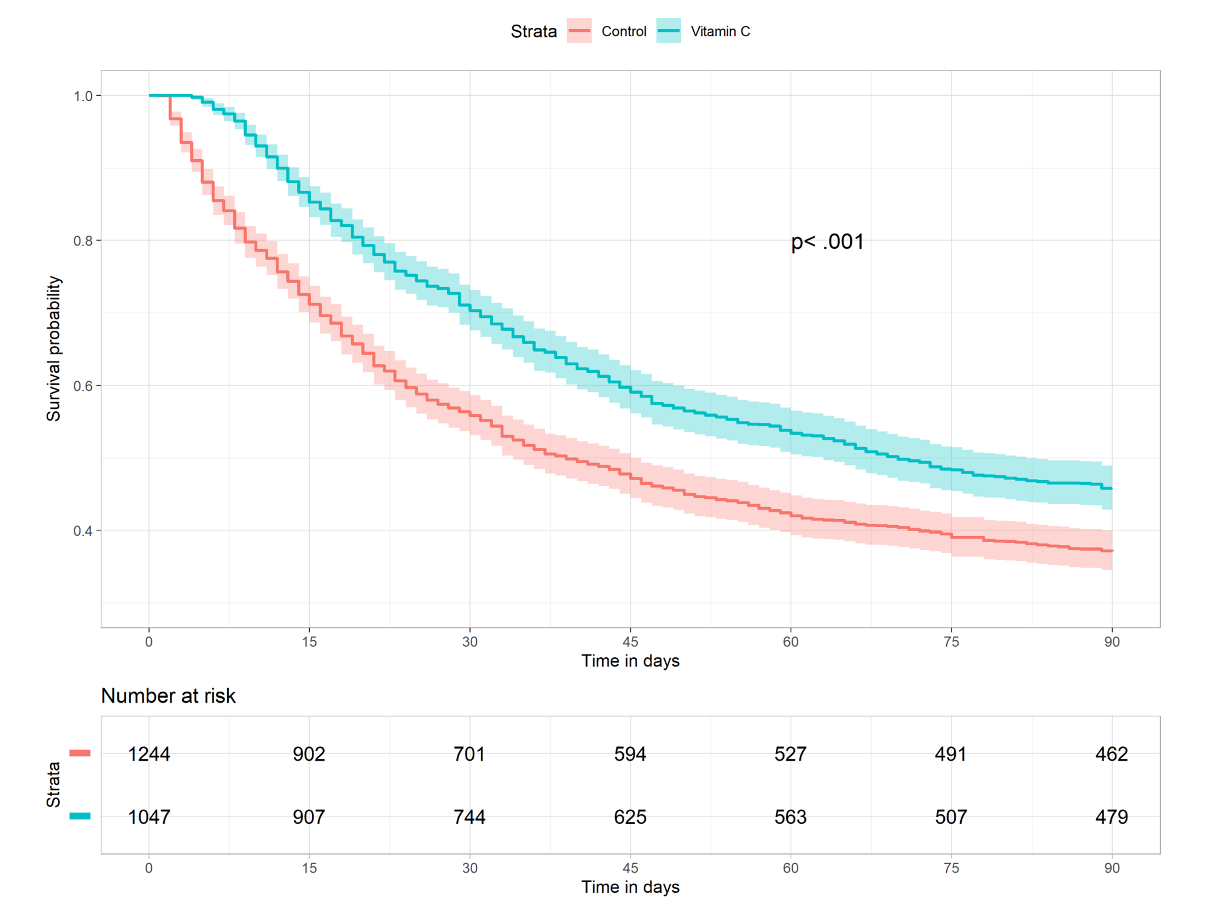


**Fig. S3** Comparison of survival benefit between vitamin C monotherapy and in combination with corticosteroids and/or thiamine in the sepsis subpopulations

**a** Age ≥70 years


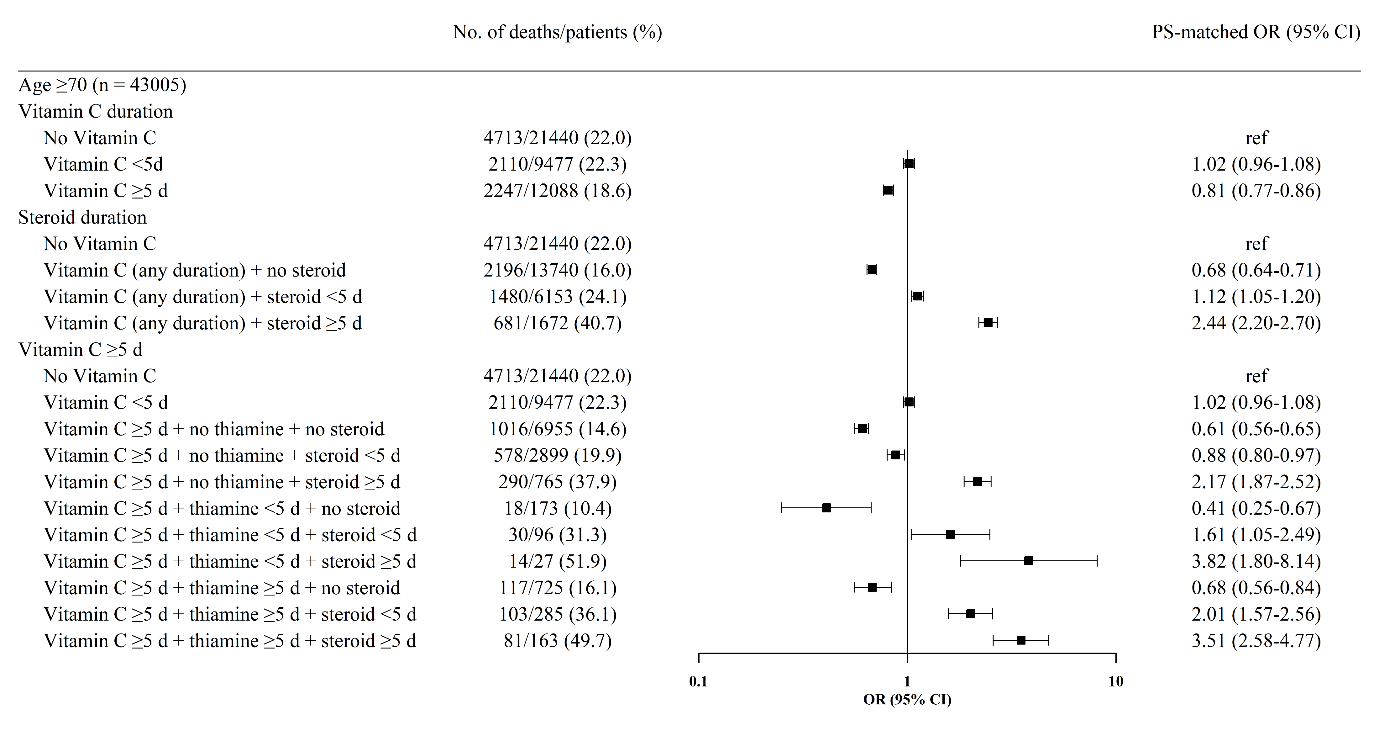


**b** Age <70 years


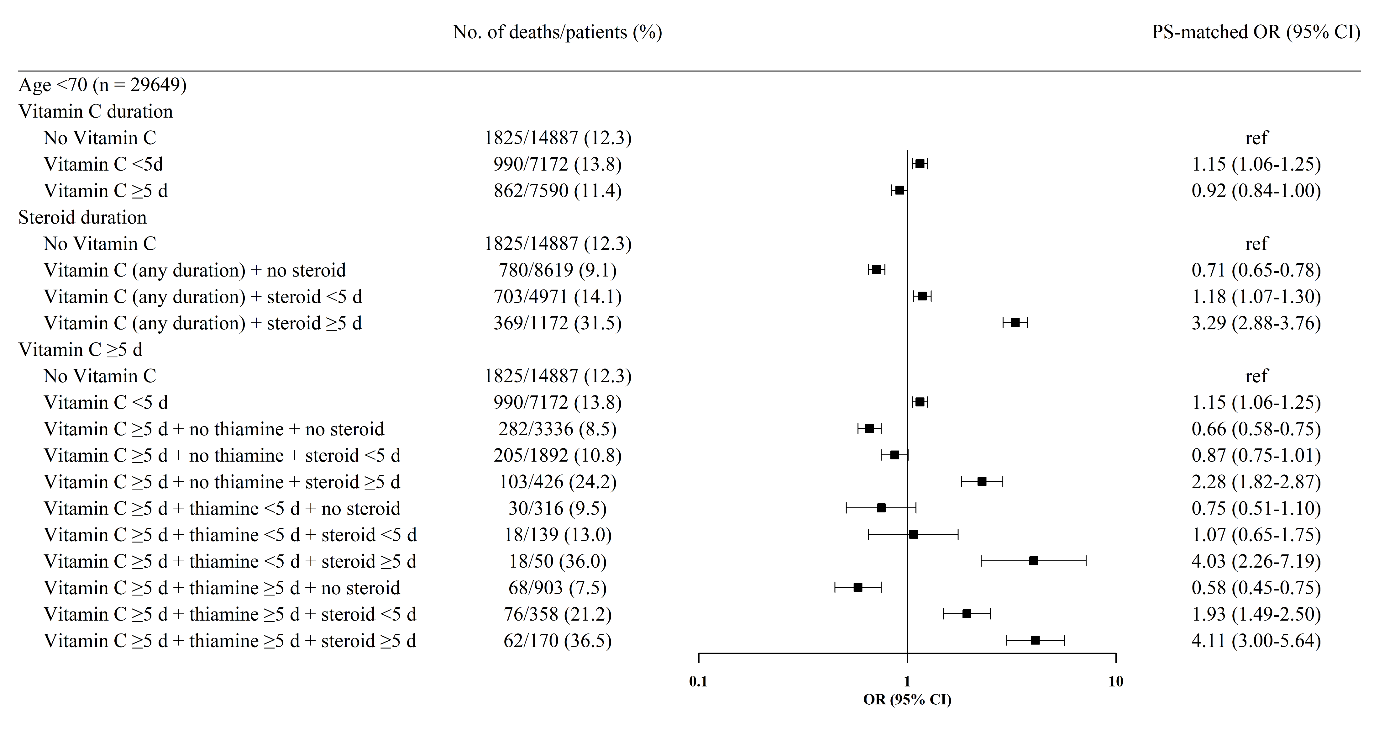


**c** Male


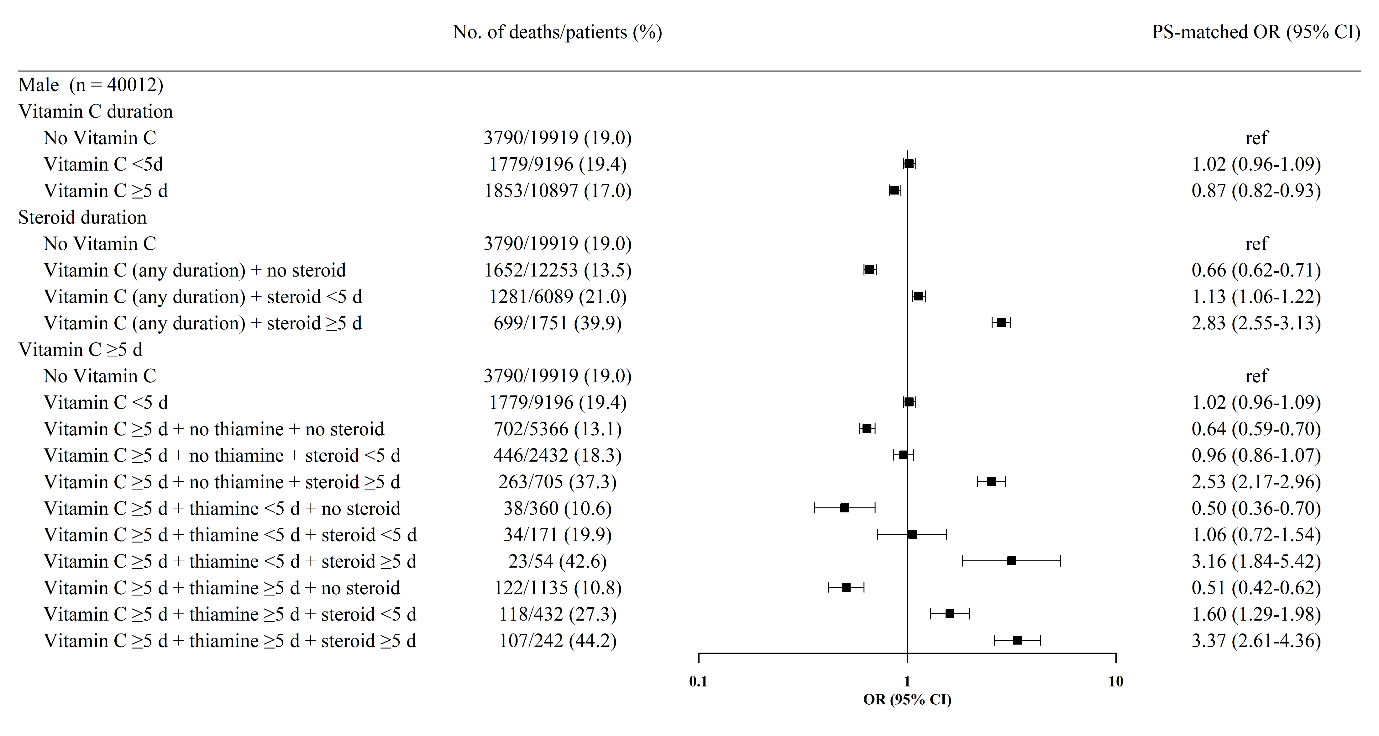


**d** Female


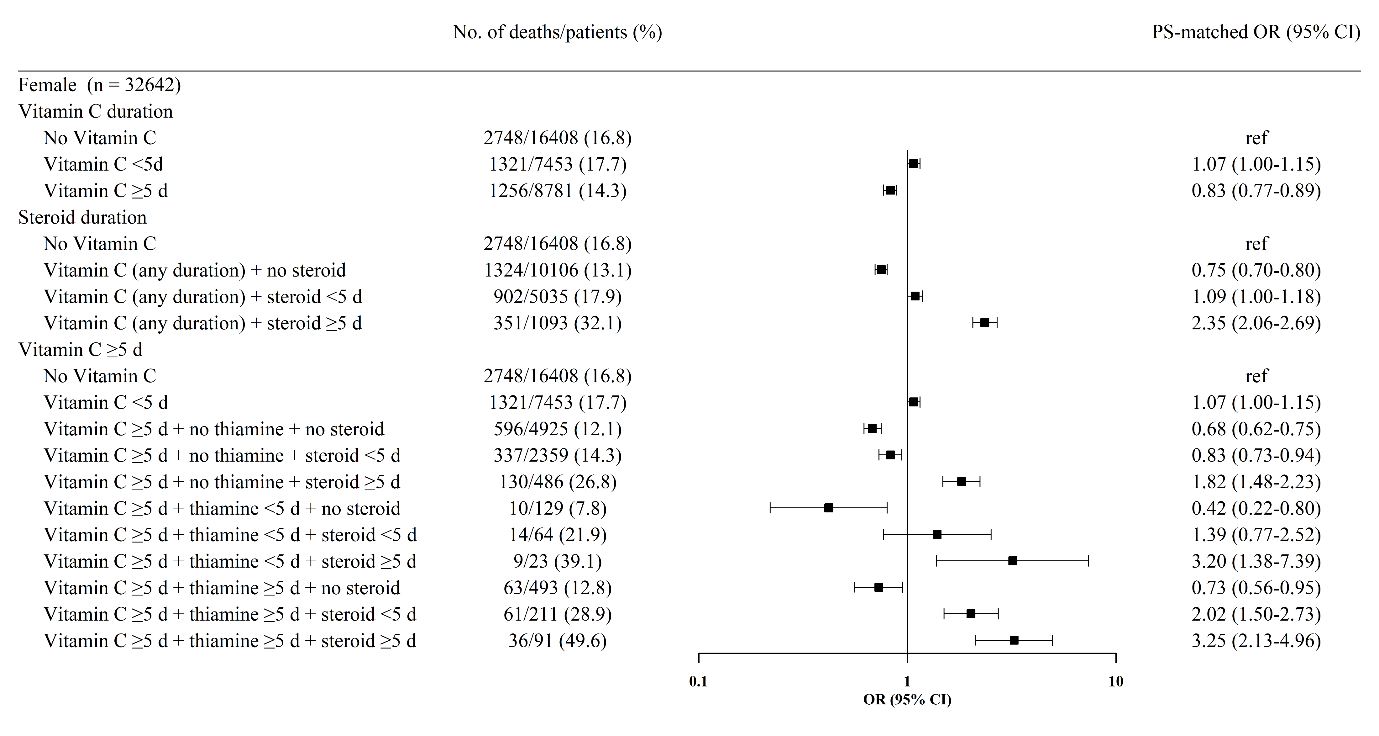


**e** Charlson Comorbidity Index ≥3


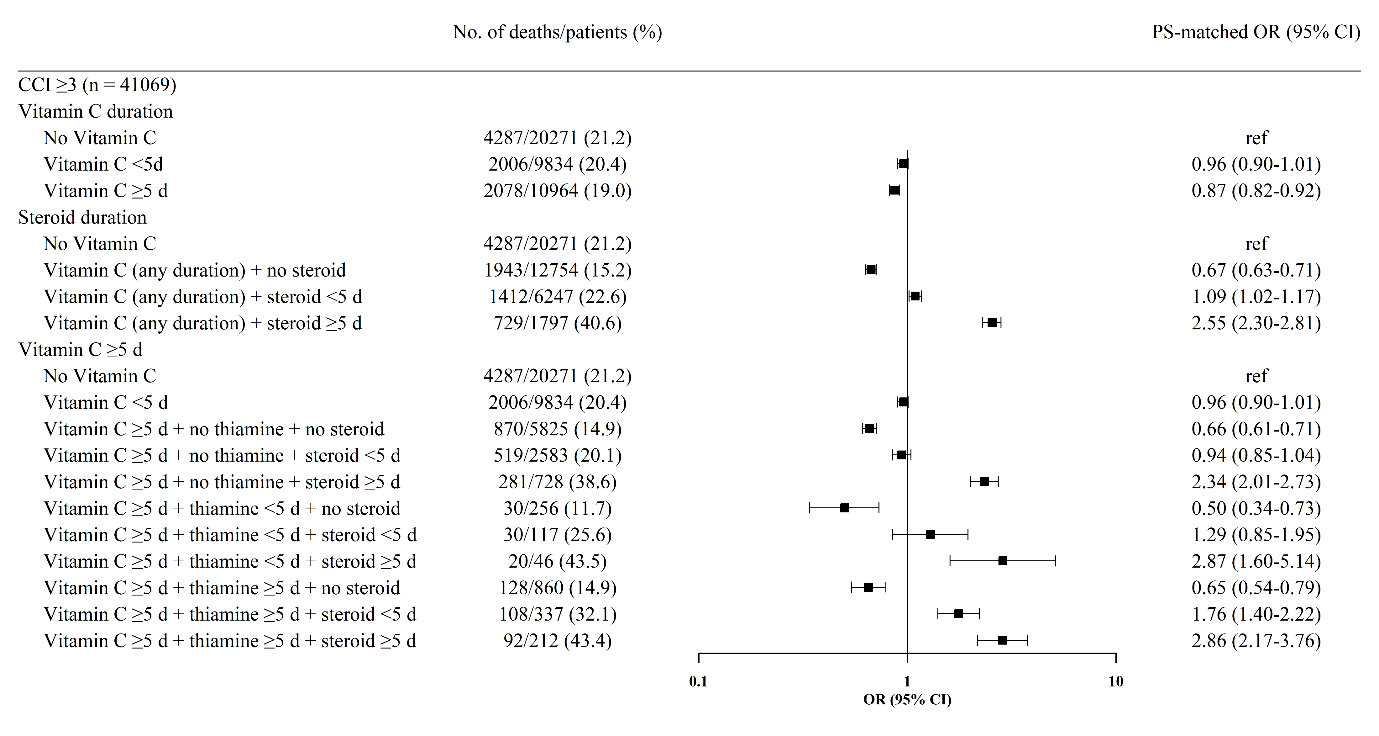


**f** Charlson Comorbidity Index <3


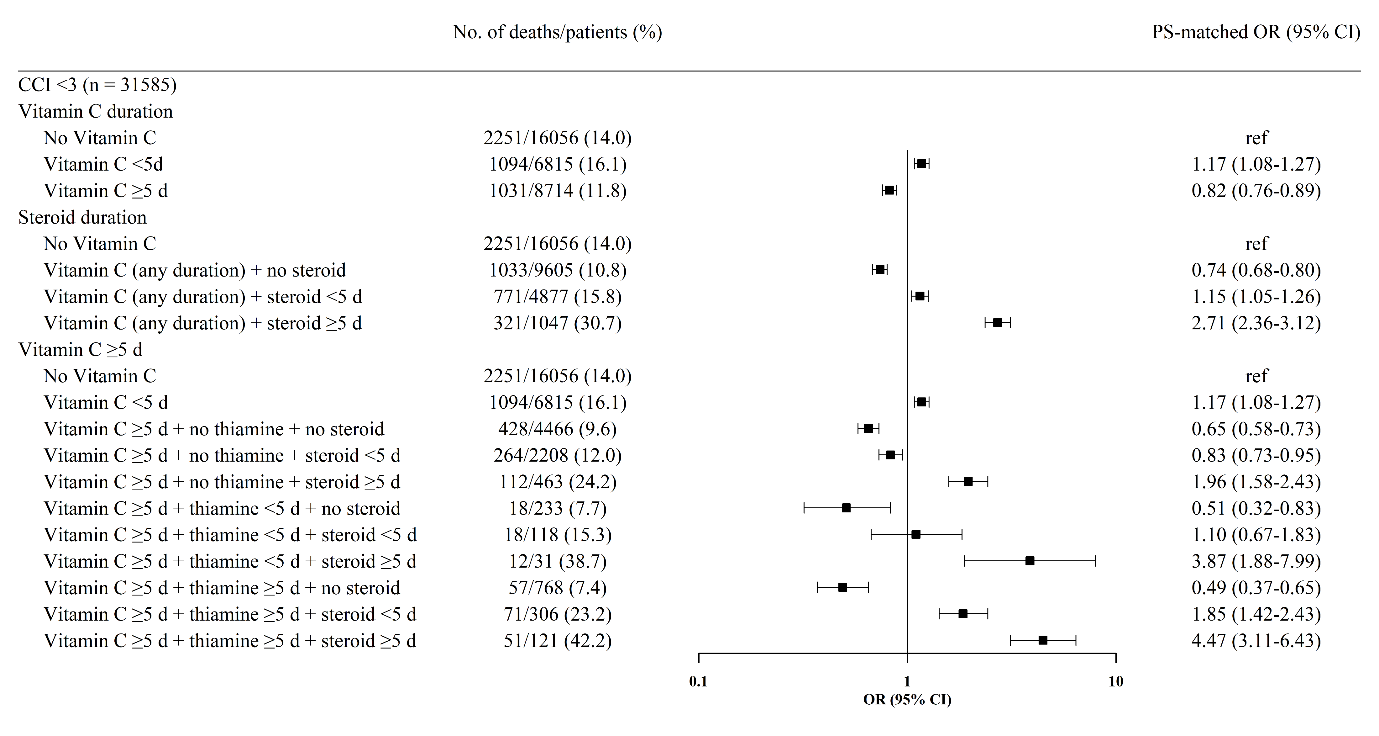


**g** Pneumonia


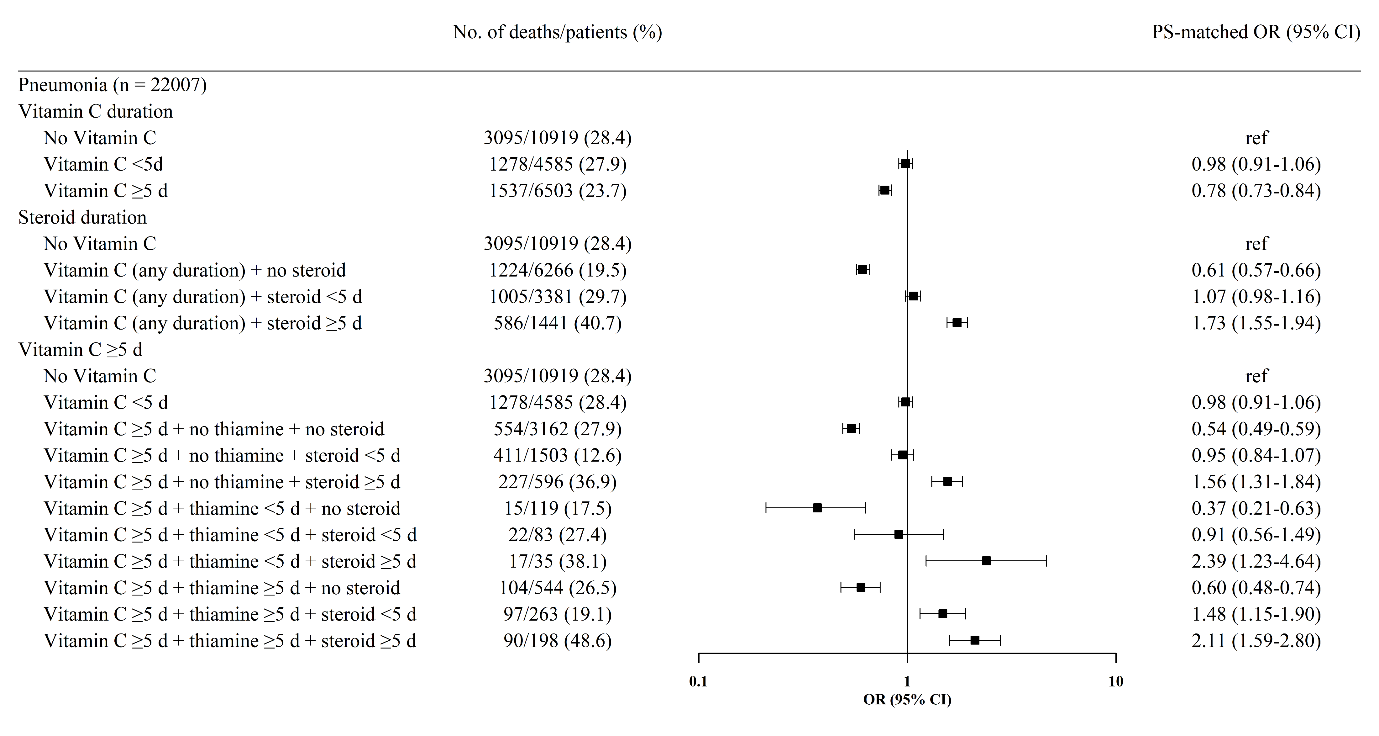


**h** Gastrointestinal


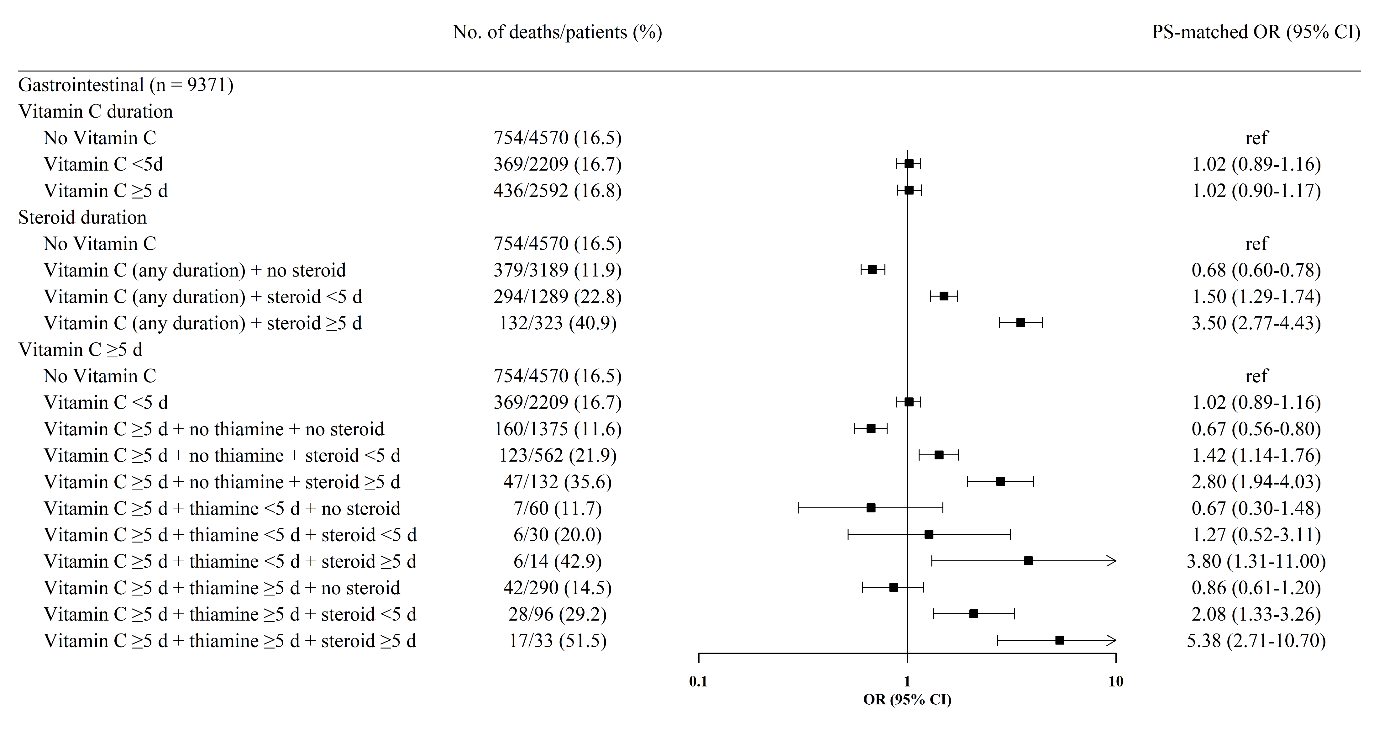


**i** Genitourinary


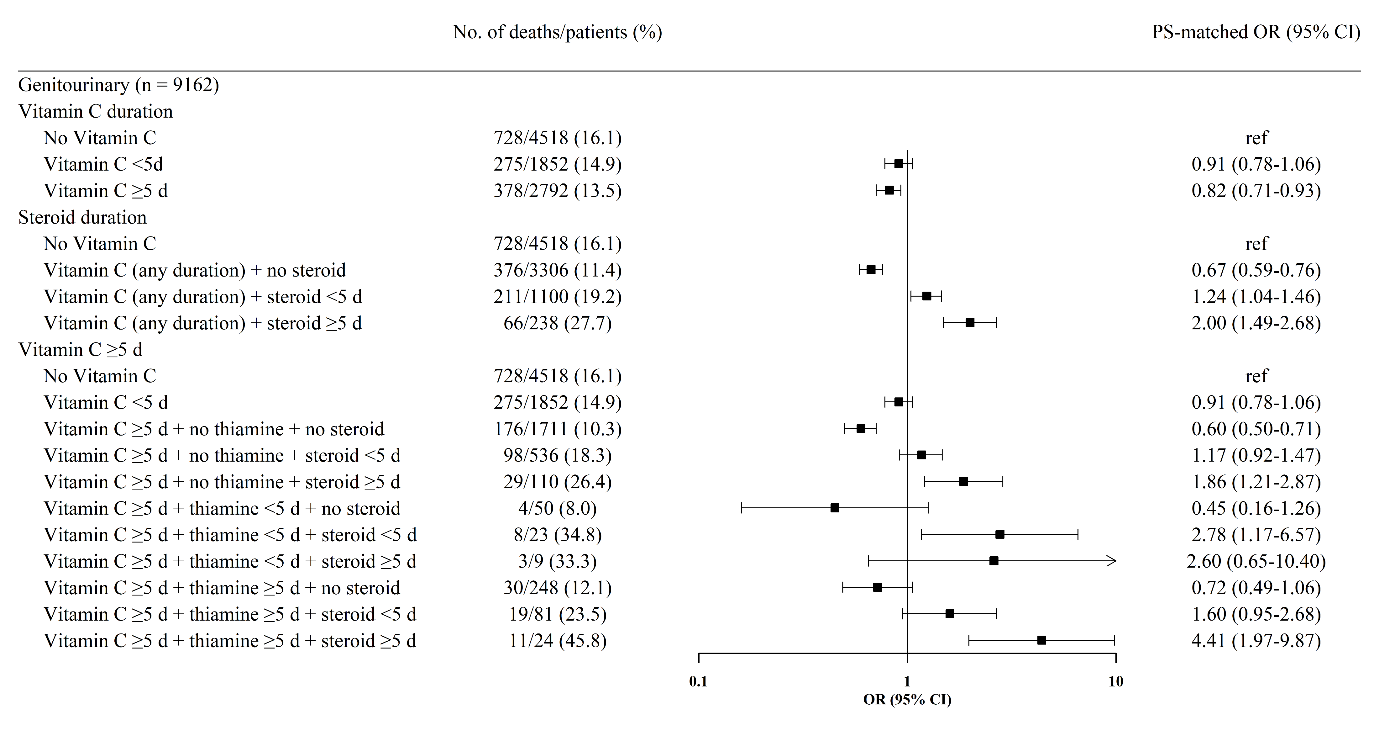


**j** Septic shock


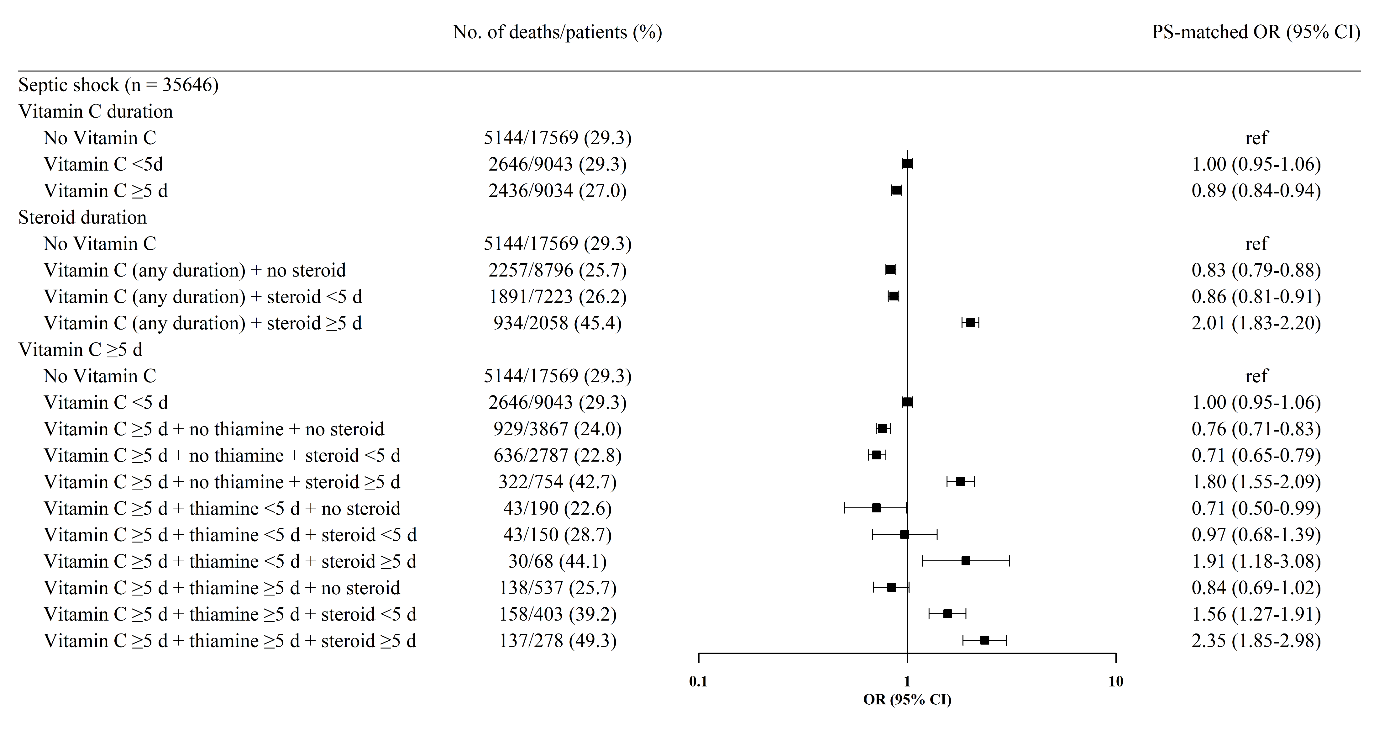


**k** Mechanical ventilation


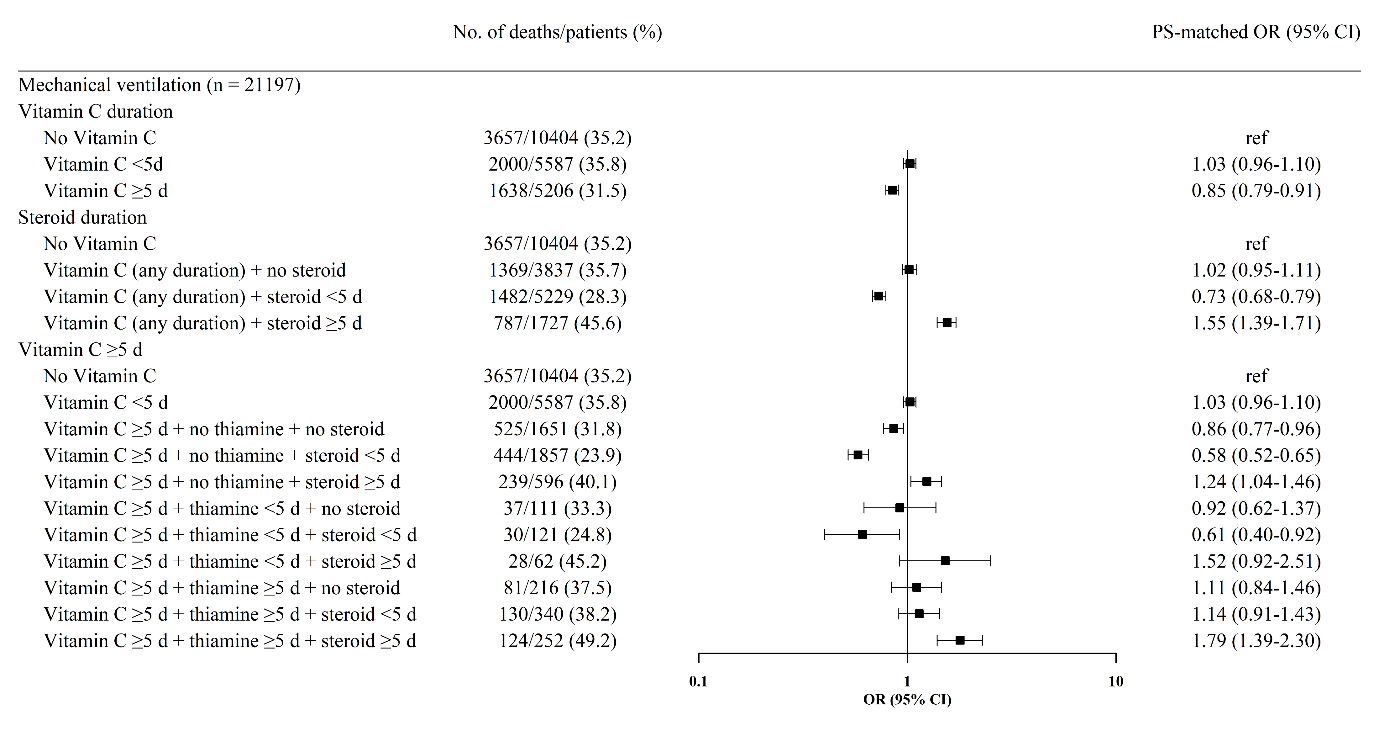


**l** Renal replacement therapy


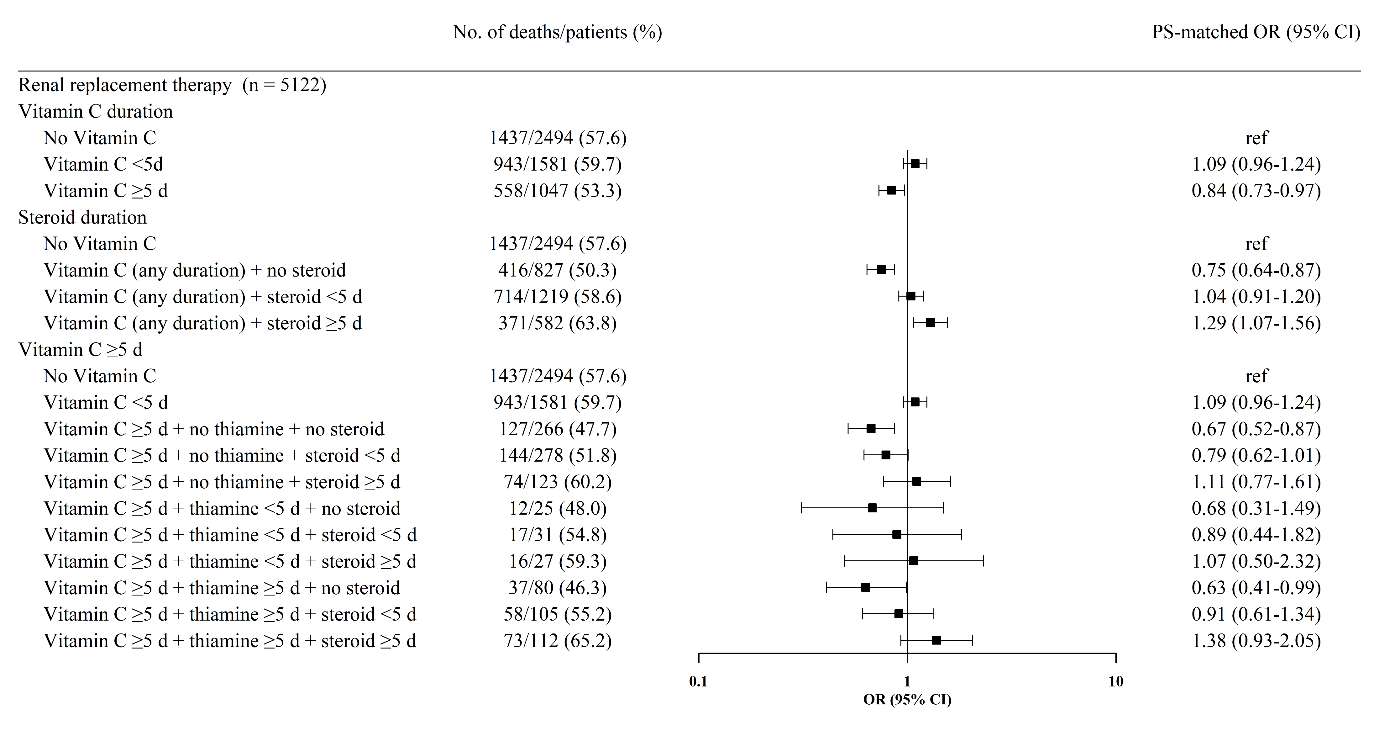


The numbers and percentages of patients who died according to each drug combination are shown. The odds ratios (ORs) and 95% confidence intervals (CIs) are calculated in the propensity score (PS)-matched treatment and control groups
